# Supplementary material for: A conserved ion channel function of STING mediates noncanonical autophagy and cell death
Source: EMBO Rep. 2024 Jan 2;25(2):10. doi: 10.1038/s44319-023-00045-x (PMC10897221; doi:10.1038/s44319-023-00045-x)
Supplement: Supplementary file 1 — Appendix [file 44319_2023_45_MOESM1_ESM.pdf]

A conserved ion channel function of STING mediates non-canonical autophagy and cell death

Jinrui Xun[1,2,*], Zhichao Zhang[3,*], Bo Lv[2,*], Defen Lu[3,*], Haoxiang Yang[2], Guijun Shang[3,4,5,#], Jay Xiaojun Tan[2,6,#]

Appendix

Appendix Figure S1--2

Appendix Figure S2--3

Appendix Figure legends--4

Appendix Figure S1

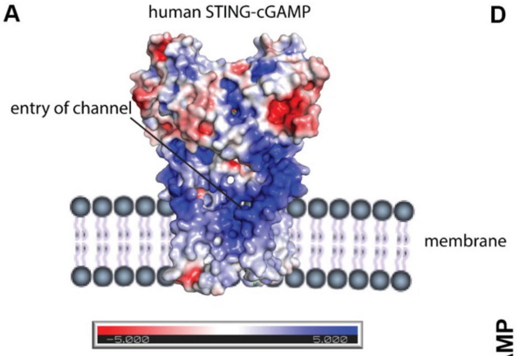

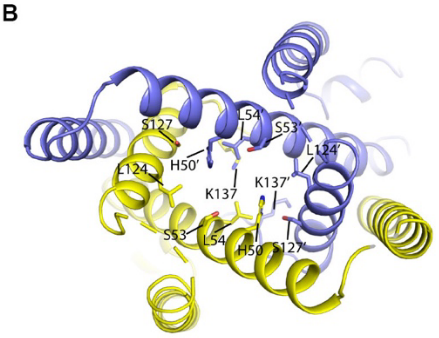

C

	STING pore mutations
1	H50L
2	S53L
3	L54E
4	K137E
5	H50A
6	H50E
7	H50Q
8	H50R
9	H50W
10	H50Y
11	S53M
12	S53W
13	S53Y
14	L54W
15	H50W S53W
16	H50Y S53Y
17	H50W/S53W/L124F
18	H50W/S53W/S127L

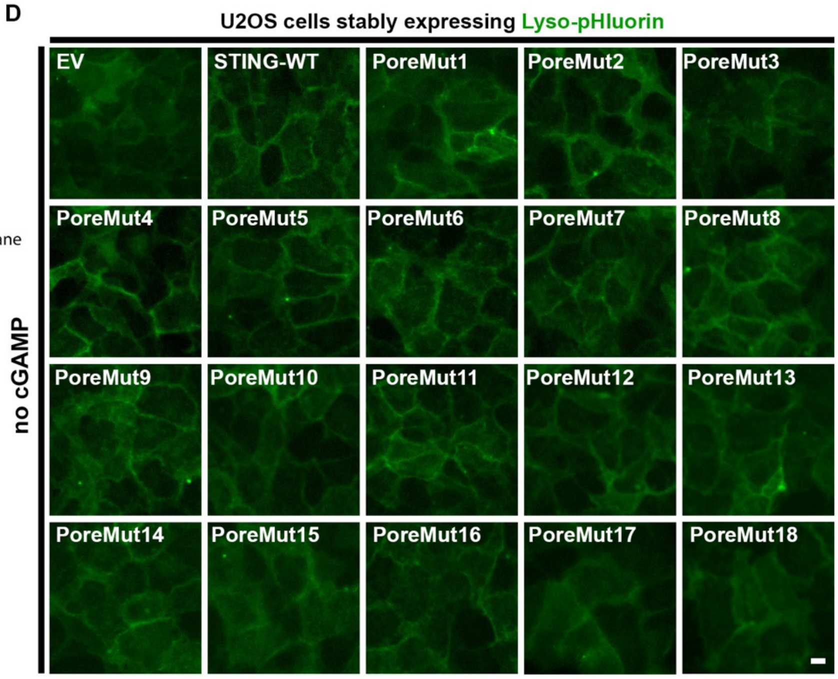

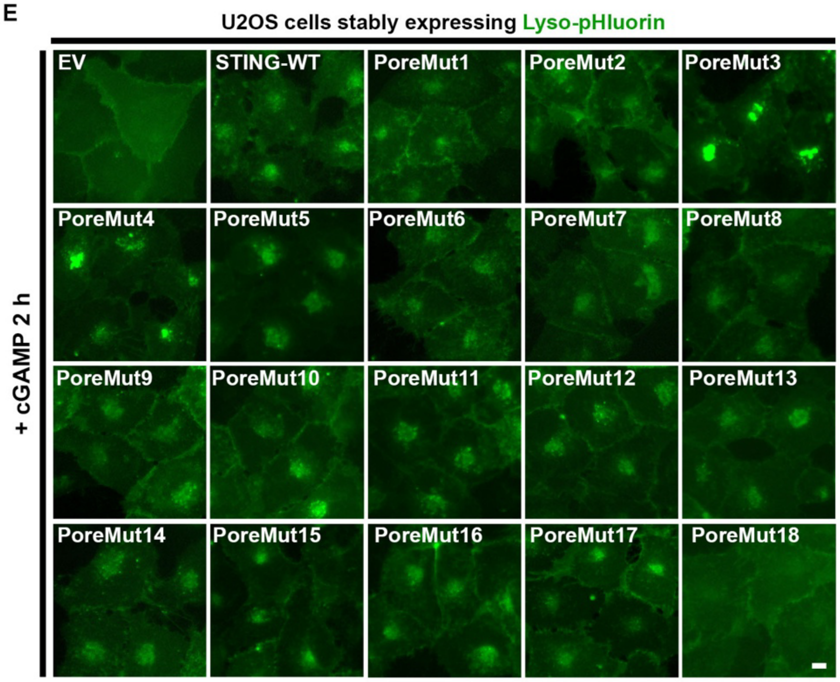

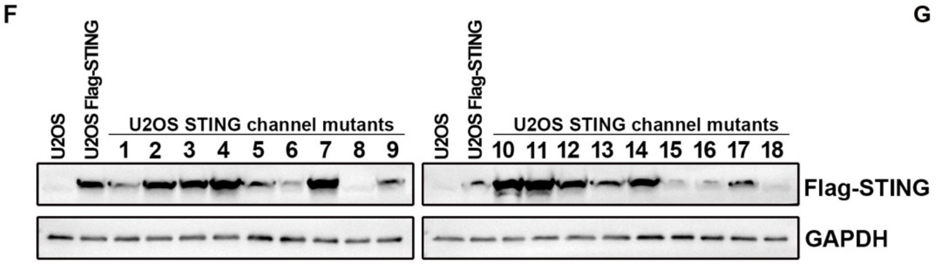

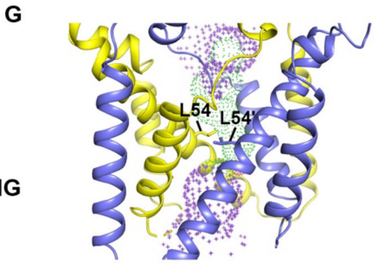

Appendix Figure S2

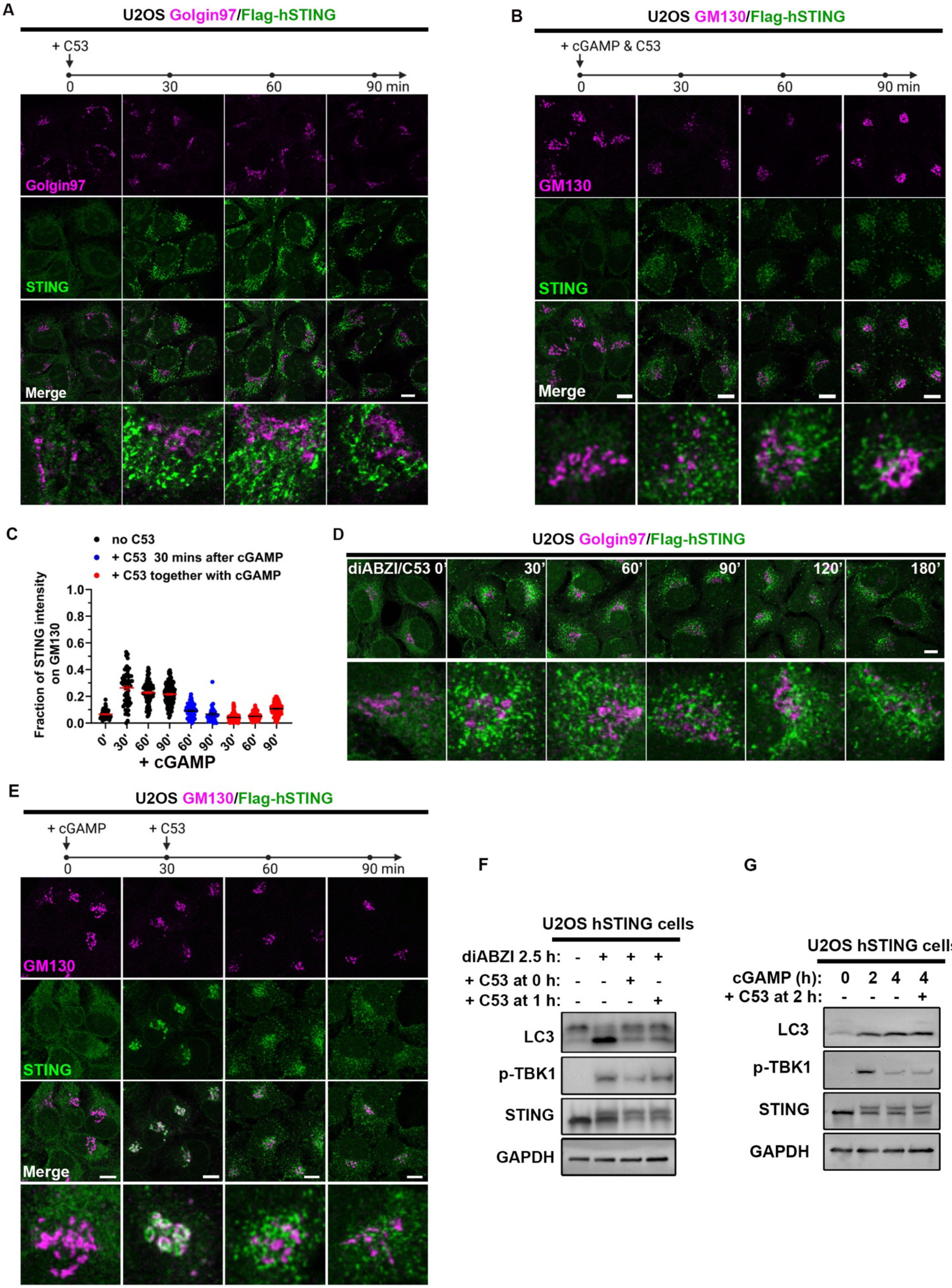

Appendix Figure Legends

Appendix Figure S1. **Testing STING channel mutants.**

A. The side view of human STING (PDB: 8IK3) with the channel entry at the cytosolic side. The electronic potential was shown. Blue and red colors represent the positively and negatively charged regions, respectively.

B. Bottom view of cGAMP bound human STING (PDB: 8IK3). The protomers are shown in cartoon with yellow and blue color, respectively. The residues lining up the pore are labelled.

C. The list of STING pore mutations tested in this study.

D. No basal lyso-pHluorin puncta were observed in U2OS cells infected by lentiviruses to stably express each STING mutants. Bar, 10 μm.

E. Most pore mutants of STING still triggered lyso-pHluorin puncta after cGAMP stimulation except Mutants #8 and #18 which were not expressed as shown in panel (F). Note that Mutant #3 (L54E) induced brighter puncta than wild-type or other mutants of STING. Bar, 10 μm.

F. Western blot analysis of the protein levels of each pore mutants of STING in U2OS cells.

G. The localization of L54 around the pore in the structure of cGAMP-bound human STING (PDB: 8IK3). The pore radii (spheres) were calculated using HOLE.

Appendix Figure S2. **Compound C53 can be used as a STING channel blocker when used after STING traffics to the Golgi or post-Golgi vesicles.**

A. Compound C53, which binds to the transmembrane pore of STING, induces STING puncta that do not colocalize with the Golgi. Monoclonal U2OS Flag-hSTING cells were stimulated with C53 alone and then fixed at indicated time points for immunostaining of STING and the trans-Golgi marker Golin97. Bar, 10 μm.

B. Compound C53 fully blocks cGAMP-induced trafficking of Flag-STING from the ER to the Golgi in U2OS cells. Monoclonal U2OS Flag-hSTING cells were stimulated with cGAMP + C53 for indicated time periods, followed by fixation and immunostaining of STING and the cis-Golgi marker GM130. See quantification in (C). Bar, 10 μm.

C. Quantification of the colocalization between STING and GM130. Mean ± SEM; n = 49, 58, 109, 147, 58, 27, 115, 96, and 93 random cells from left to right.

D. C53 fully blocks diABZI-induced trafficking of Flag-STING from the ER to the Golgi in U2OS cells. Monoclonal U2OS Flag-hSTING cells were stimulated with diABZI + C53 for indicated time periods, followed by fixation and immunostaining of STING and the trans-Golgi marker Golgin97.

E. C53 addition 30 min after cGAMP allows STING trafficking to the Golgi and post-Golgi vesicles. Monoclonal U2OS cells stably expressing hSTING were stimulated as indicated and then fixed for the immunostaining of STING and GM130. See quantification in (C). Bar, 10 μm.

F. C53 addition 1 hour after diABZI fully blocks STING-dependent LC3 lipidation. Monoclonal U2OS Flag-hSTING cells were stimulated as indicated and whole cell lysates were harvested for western blot 2.5 hours after diABZI treatment.

G. C53 addition 2 hours after cGAMP stimulation failed to block STING-dependent LC3 lipidation. The same cells in (E) were treated as indicated and harvested for western blot.
