# Supplementary figures and images for: A conserved ion channel function of STING mediates noncanonical autophagy and cell death
Source: EMBO Rep. 2024 Jan 2;25(2):10. doi: 10.1038/s44319-023-00045-x (PMC10897221; doi:10.1038/s44319-023-00045-x)

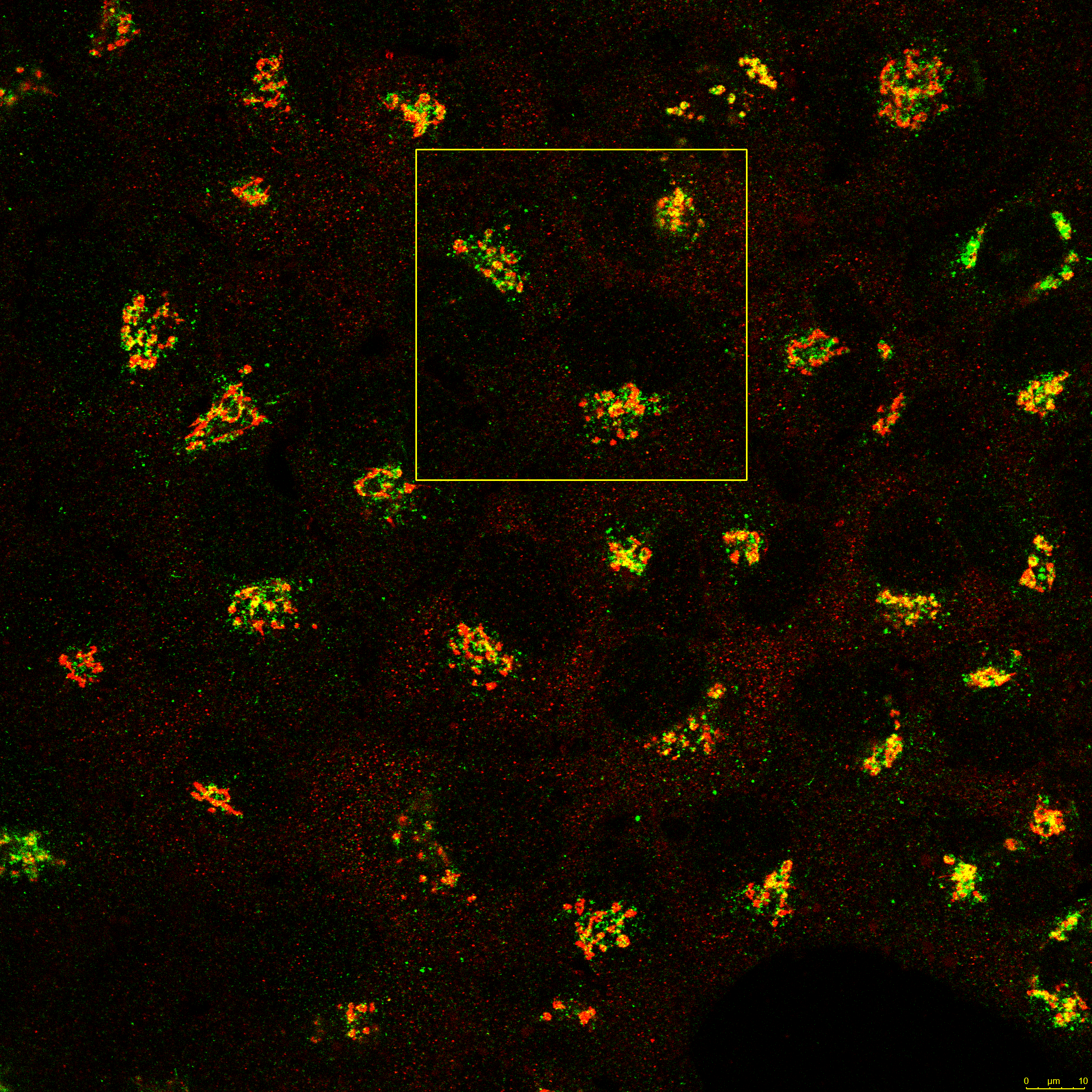

Supplement: Supplementary file 2 — Source Data Fig. 2 [file 44319_2023_45_MOESM2_ESM.zip › Fig 2/Fig 2A/F2A3 GM130-594, FST-488_cGAMP 60'.png]

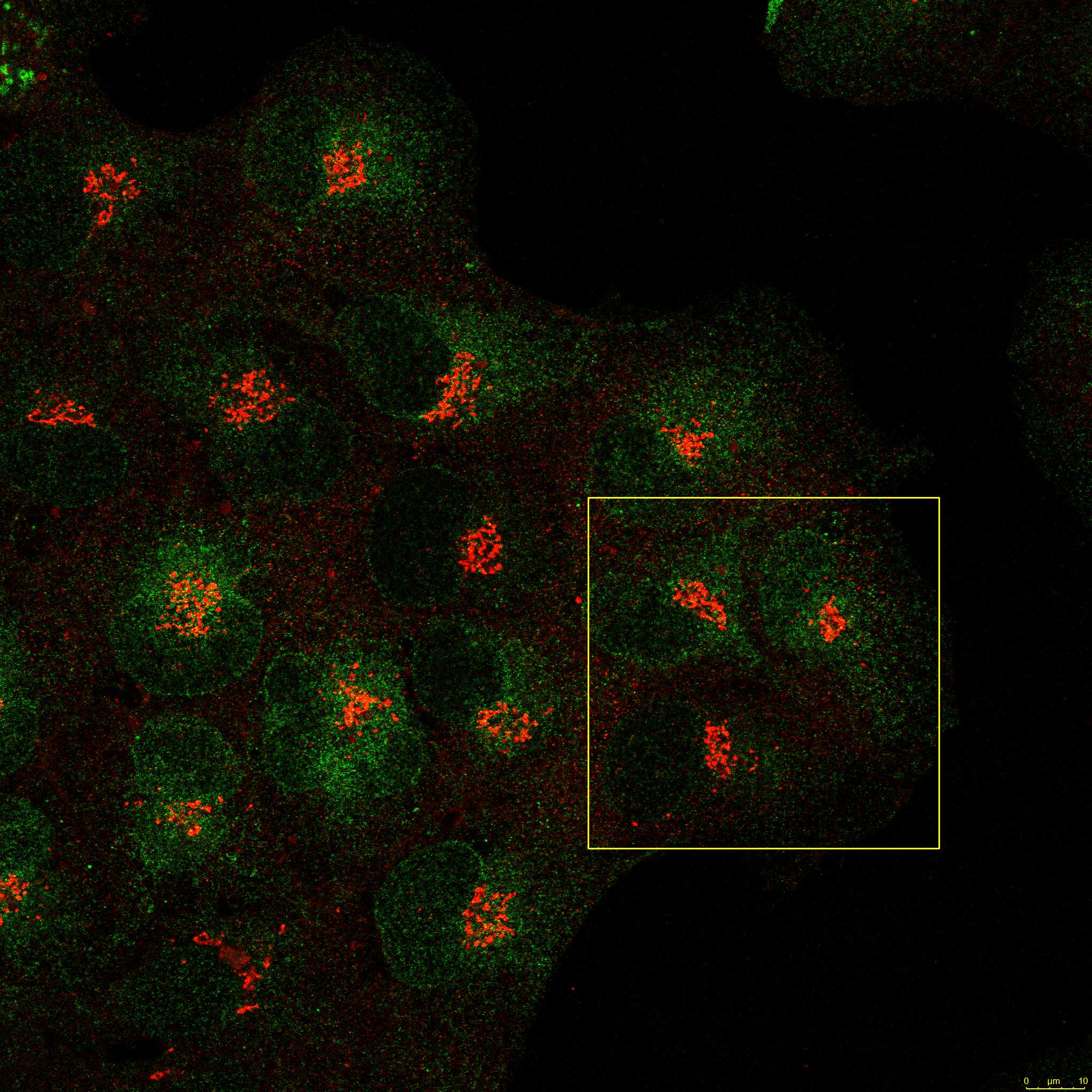

Supplement: Supplementary file 2 — Source Data Fig. 2 [file 44319_2023_45_MOESM2_ESM.zip › Fig 2/Fig 2A/F2A1 GM130-594, FST-488_cGAMP 0'.png]

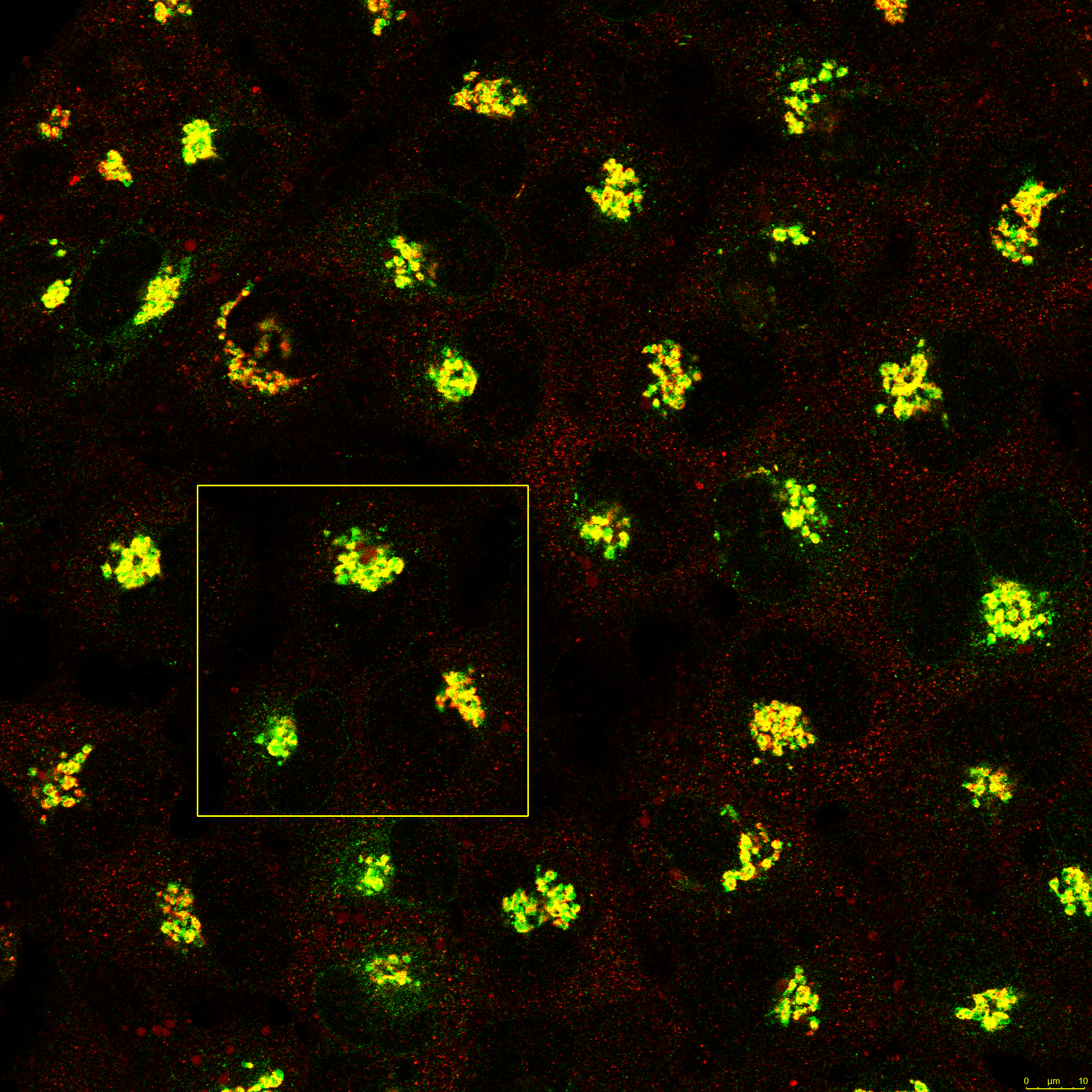

Supplement: Supplementary file 2 — Source Data Fig. 2 [file 44319_2023_45_MOESM2_ESM.zip › Fig 2/Fig 2A/F2A2 GM130-594, FST-488_cGAMP 30'.png]

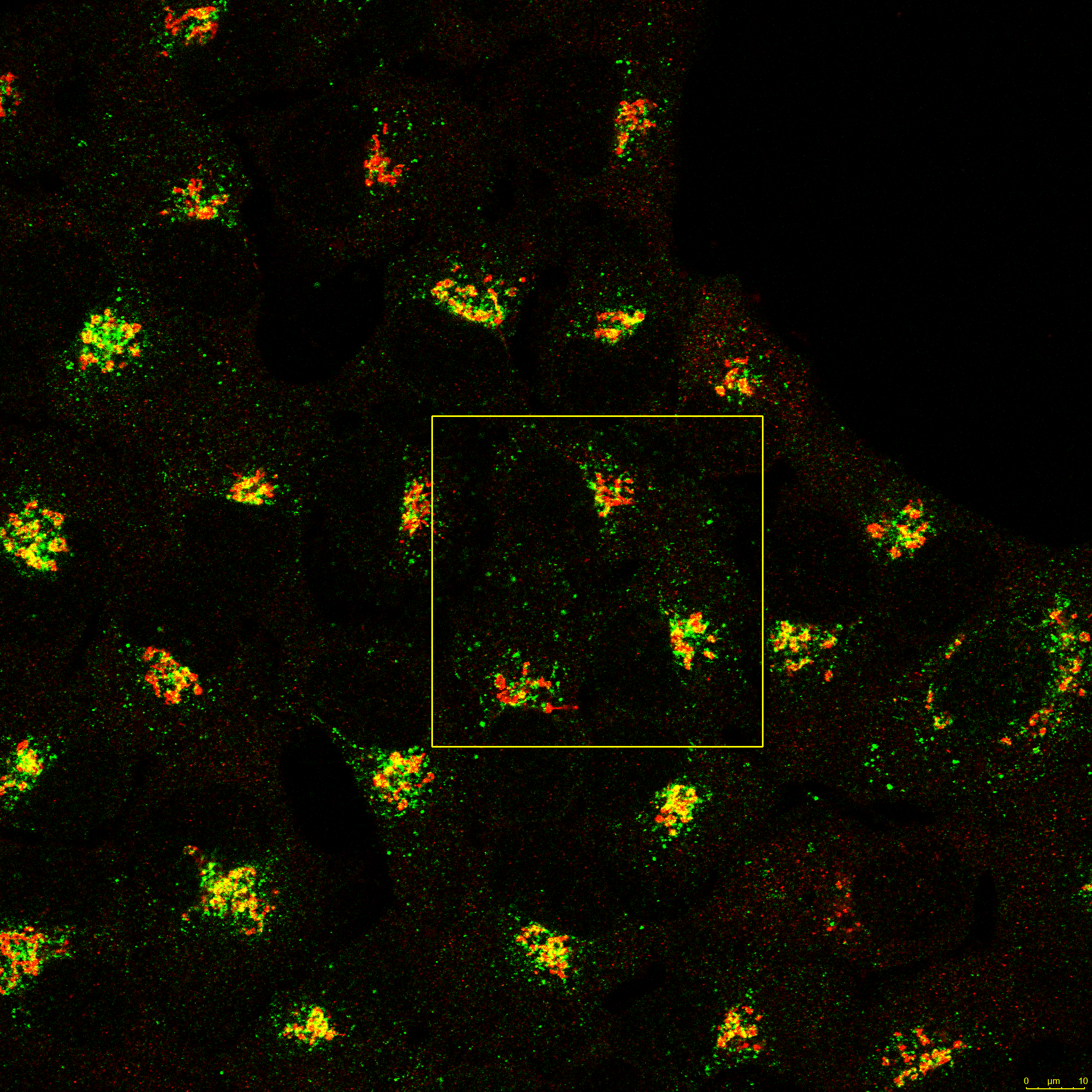

Supplement: Supplementary file 2 — Source Data Fig. 2 [file 44319_2023_45_MOESM2_ESM.zip › Fig 2/Fig 2A/F2A4 GM130-594, FST-488_cGAMP 90'.png]

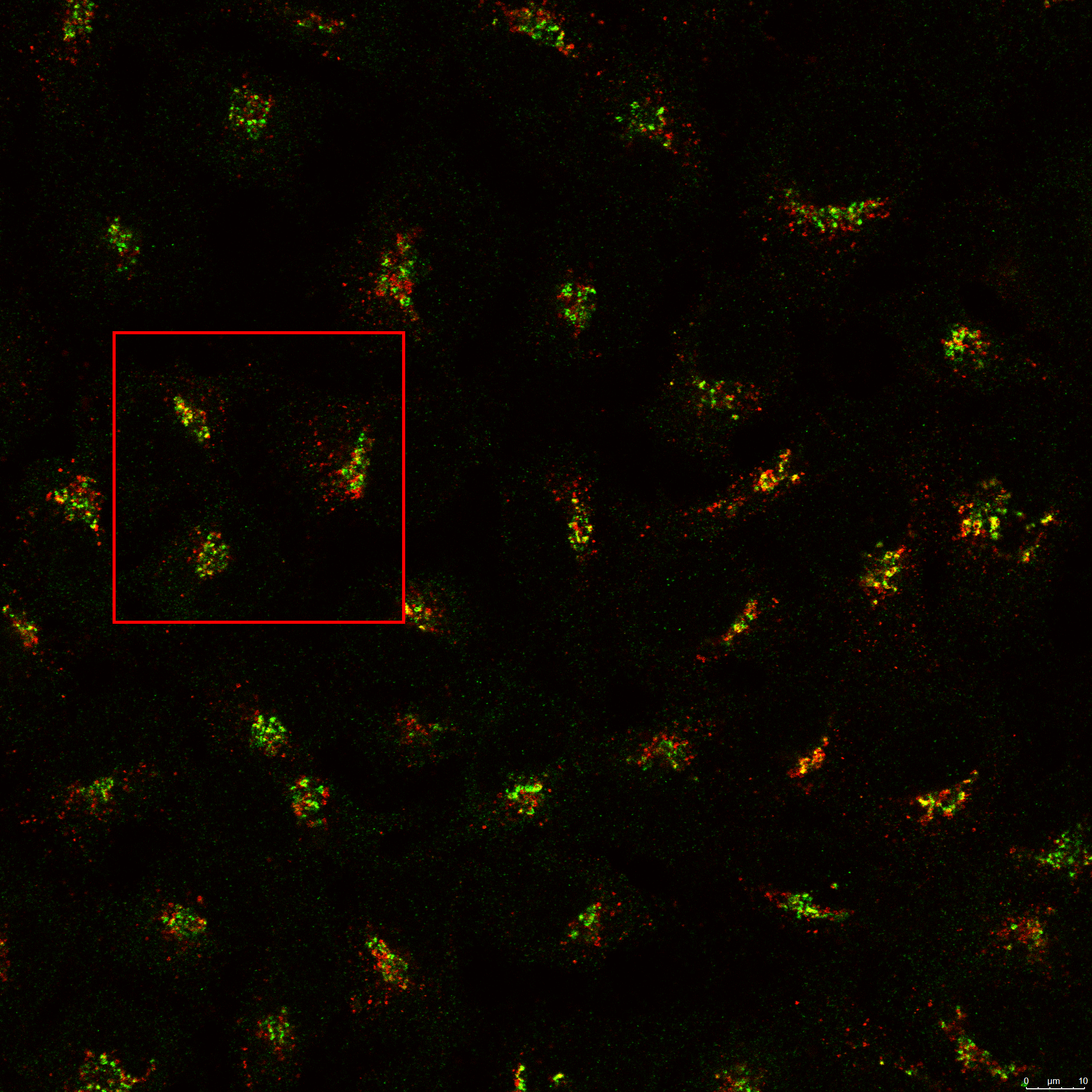

Supplement: Supplementary file 2 — Source Data Fig. 2 [file 44319_2023_45_MOESM2_ESM.zip › Fig 2/Fig 2G/F2G-2 TGN46-594, Golgin97-488_cGAMP 30'.tif]

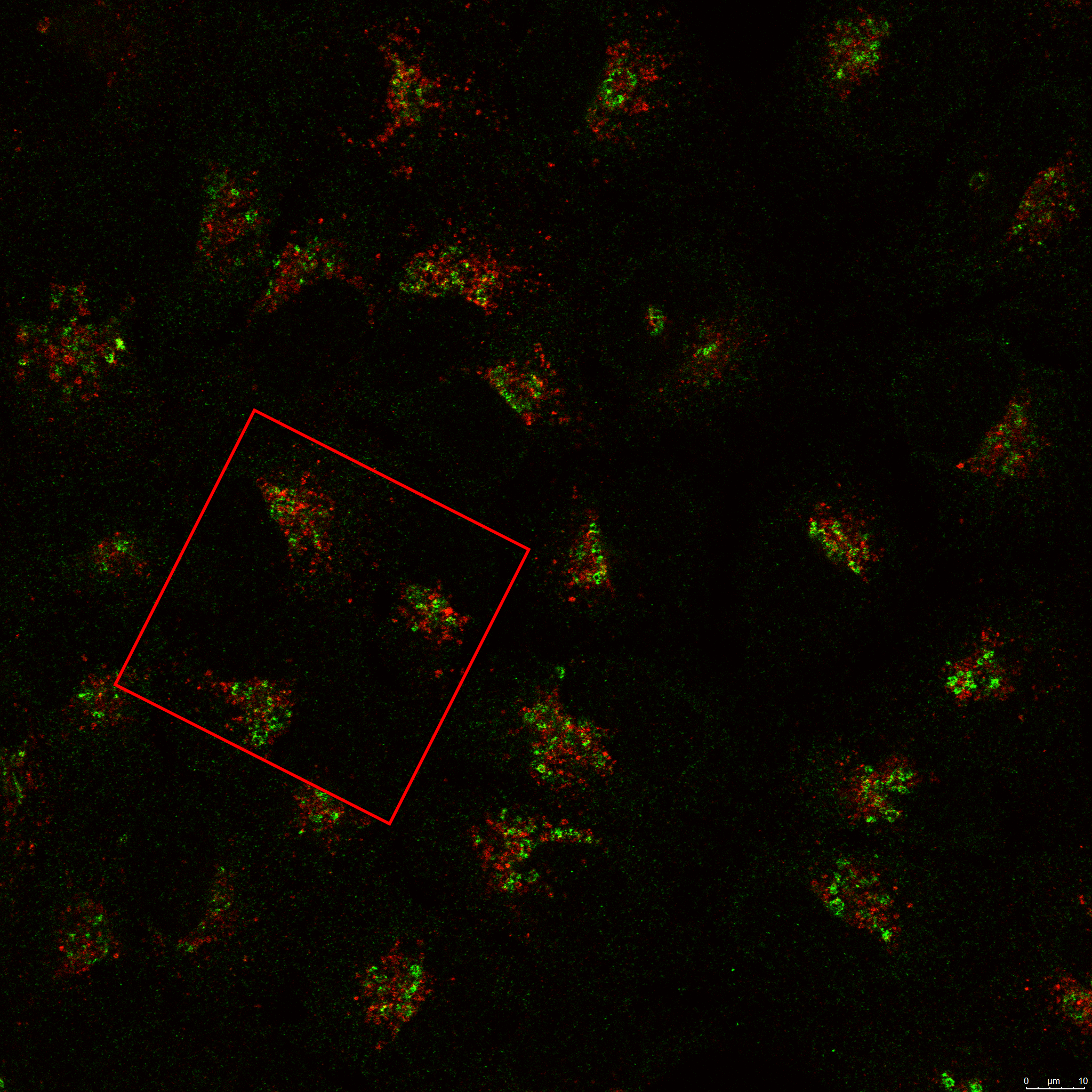

Supplement: Supplementary file 2 — Source Data Fig. 2 [file 44319_2023_45_MOESM2_ESM.zip › Fig 2/Fig 2G/F2G-3 TGN46-594, Golgin97-488_cGAMP 60'.tif]

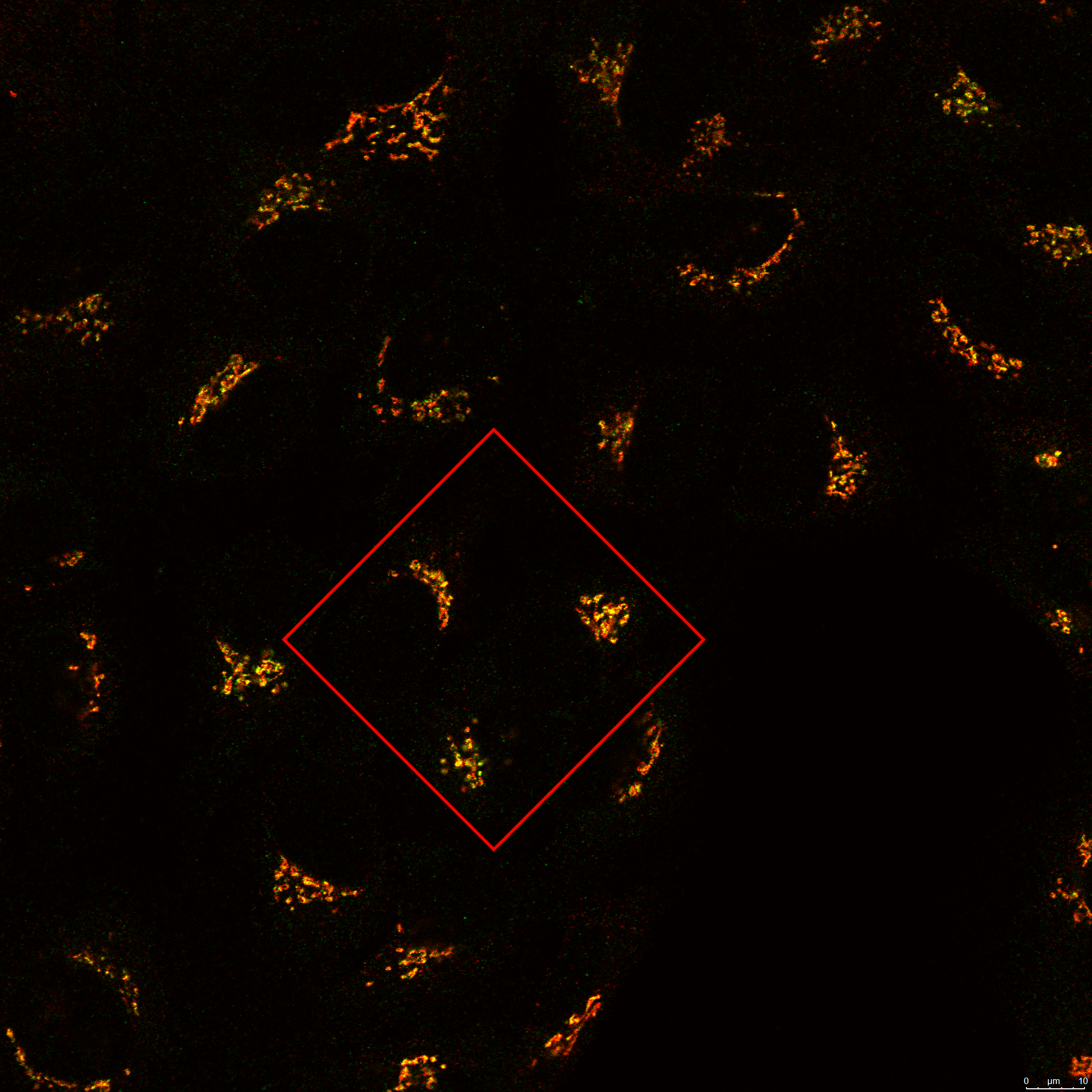

Supplement: Supplementary file 2 — Source Data Fig. 2 [file 44319_2023_45_MOESM2_ESM.zip › Fig 2/Fig 2G/F2G-1 TGN46-594, Golgin97-488_cGAMP 0'.tif]

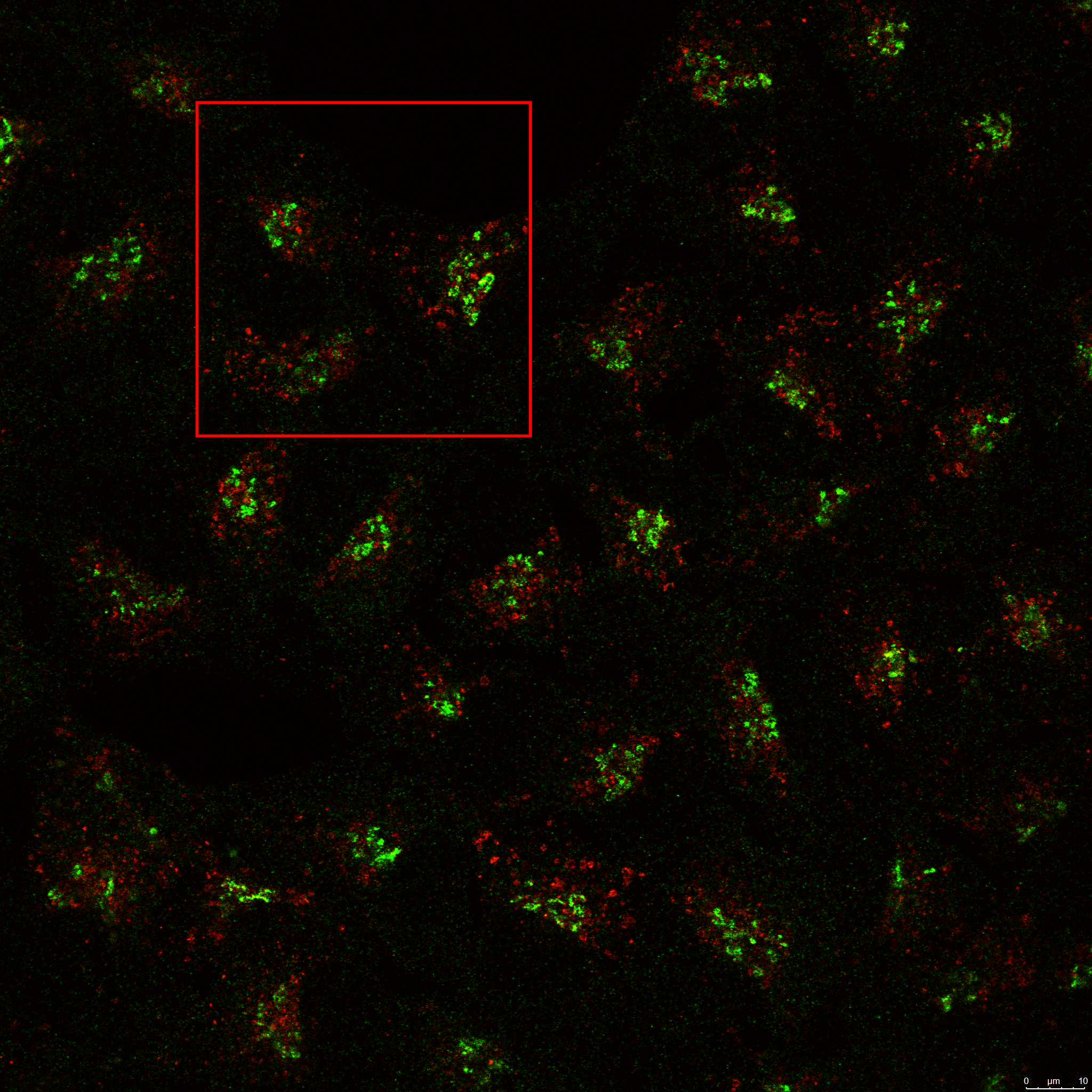

Supplement: Supplementary file 2 — Source Data Fig. 2 [file 44319_2023_45_MOESM2_ESM.zip › Fig 2/Fig 2G/F2G-4 TGN46-594, Golgin97-488_cGAMP 90'.tif]

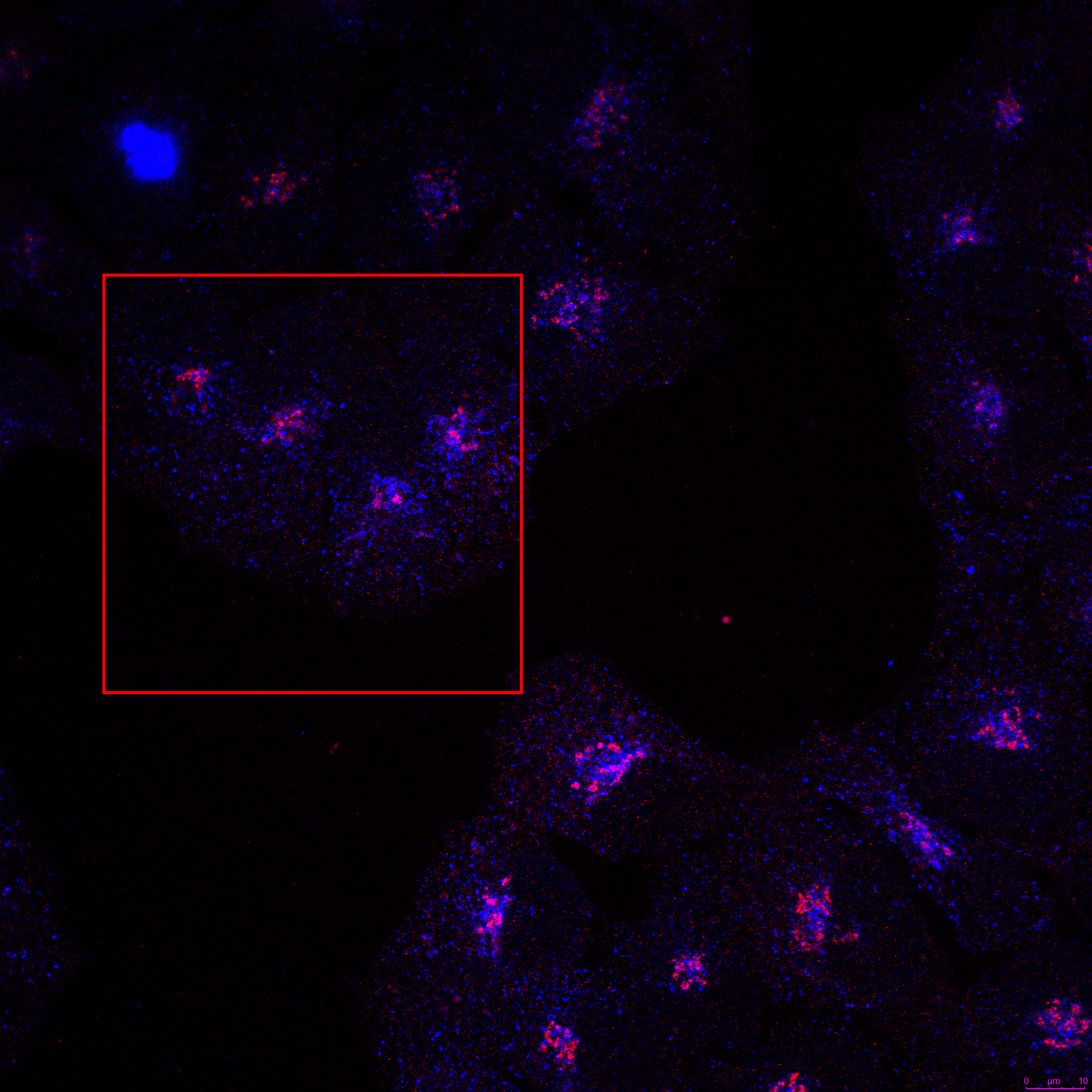

Supplement: Supplementary file 2 — Source Data Fig. 2 [file 44319_2023_45_MOESM2_ESM.zip › Fig 2/Fig 2D/F2D4 U2OS FST-blue golgin97-594_cGAMP-90min.tif]

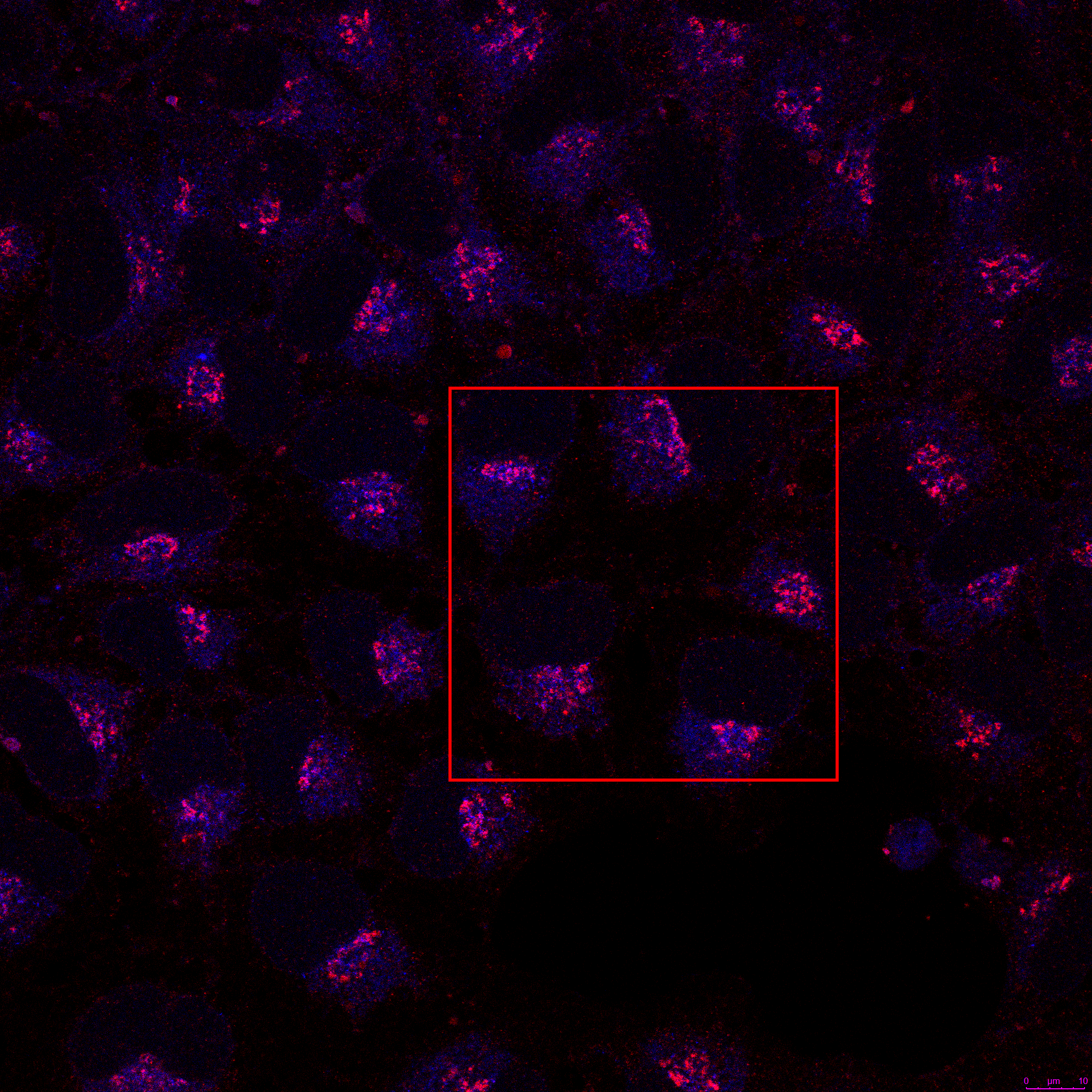

Supplement: Supplementary file 2 — Source Data Fig. 2 [file 44319_2023_45_MOESM2_ESM.zip › Fig 2/Fig 2D/F2D3 U2OS FST-blue golgin97-594_cGAMP-60min.tif]

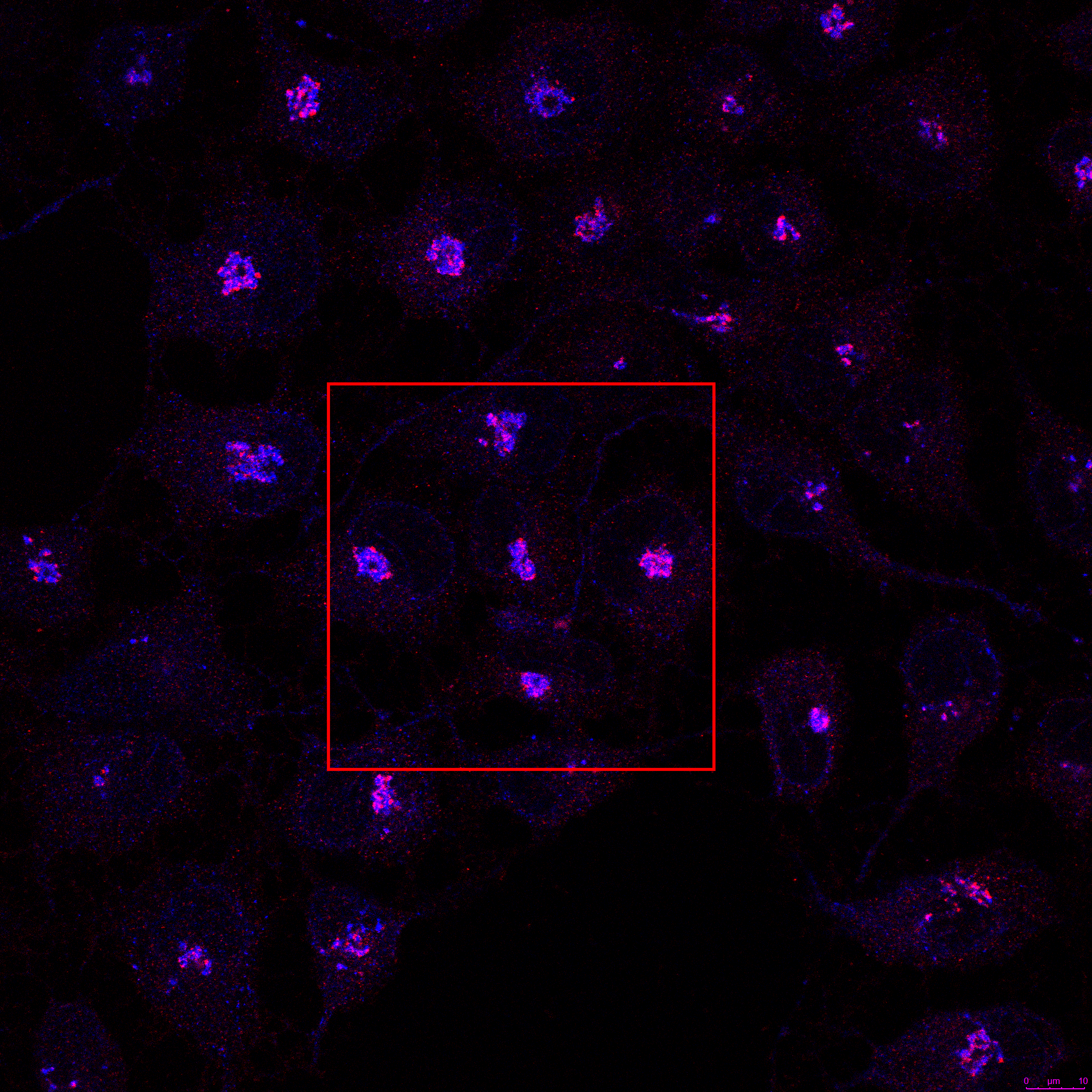

Supplement: Supplementary file 2 — Source Data Fig. 2 [file 44319_2023_45_MOESM2_ESM.zip › Fig 2/Fig 2D/F2D2 U2OS FST-blue golgin97-594_cGAMP-30min.tif]

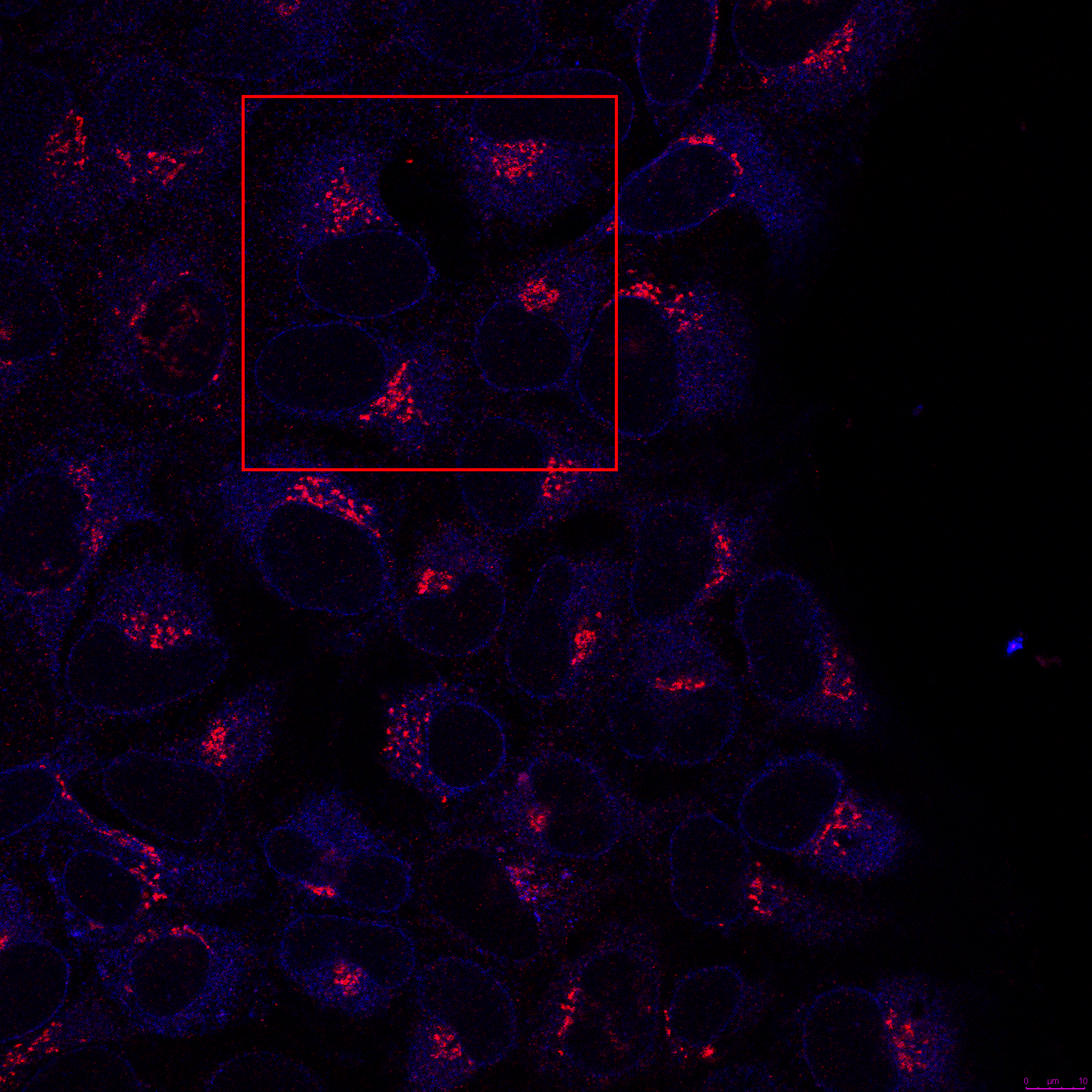

Supplement: Supplementary file 2 — Source Data Fig. 2 [file 44319_2023_45_MOESM2_ESM.zip › Fig 2/Fig 2D/F2D1 U2OS FST-blue golgin97-594_cGAMP-0min.tif]

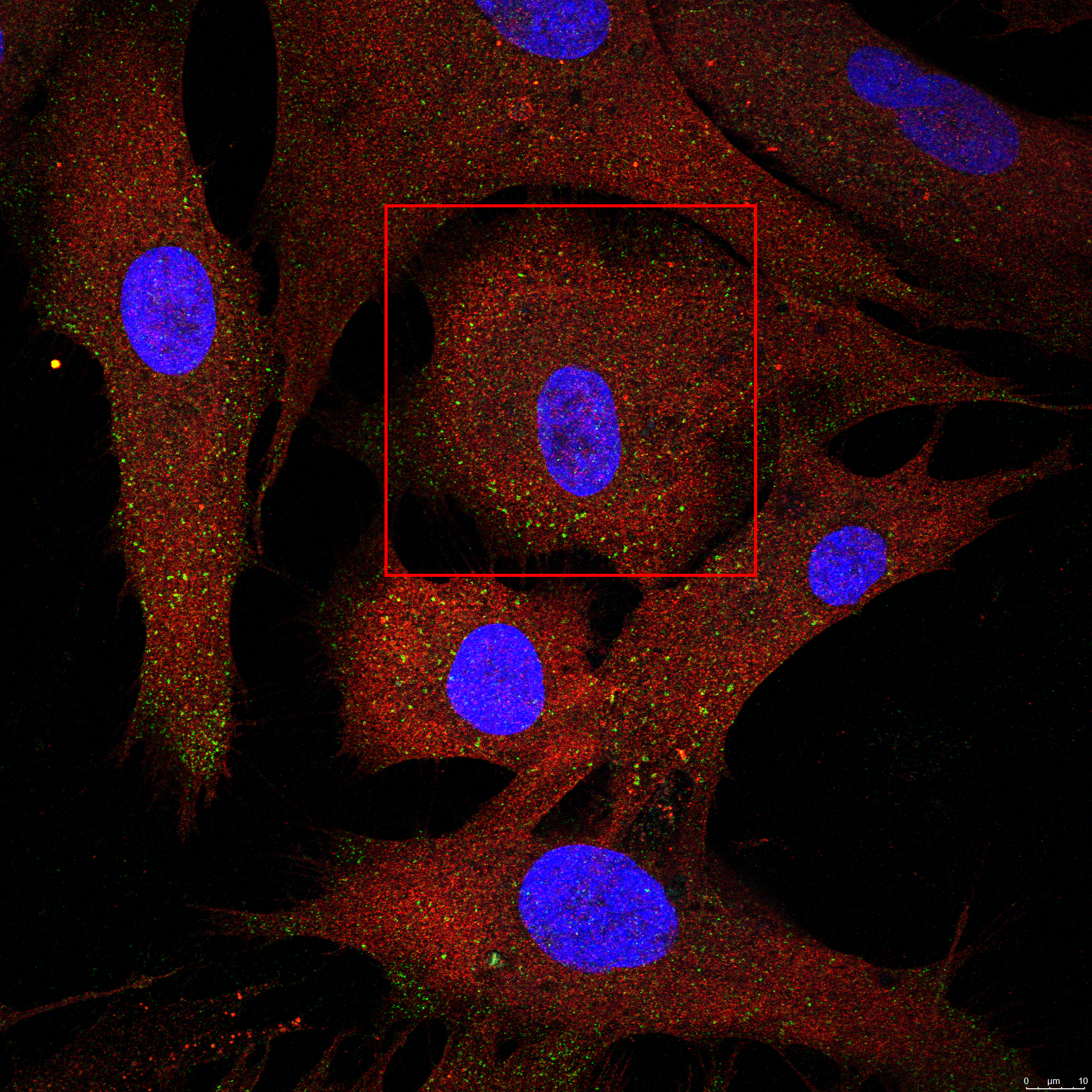

Supplement: Supplementary file 3 — Source Data Fig. 3 [file 44319_2023_45_MOESM3_ESM.zip › Fig 3/Fig 3D/F3D2 BJ LC3-R594, RAB5-M488_cGAMP 30'.tif]

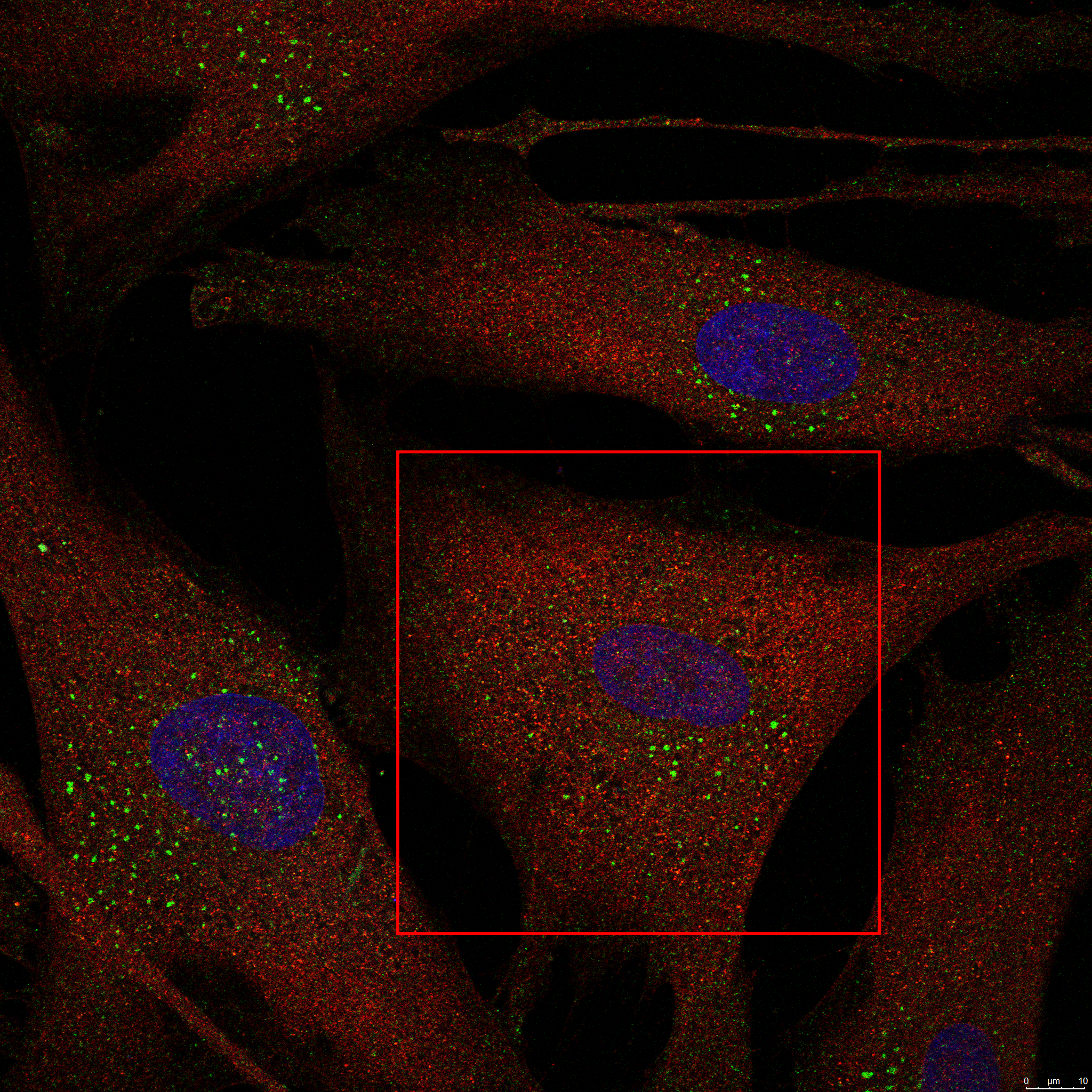

Supplement: Supplementary file 3 — Source Data Fig. 3 [file 44319_2023_45_MOESM3_ESM.zip › Fig 3/Fig 3D/F3D1 BJ LC3-R594, RAB5-M488_cGAMP 0'.tif]

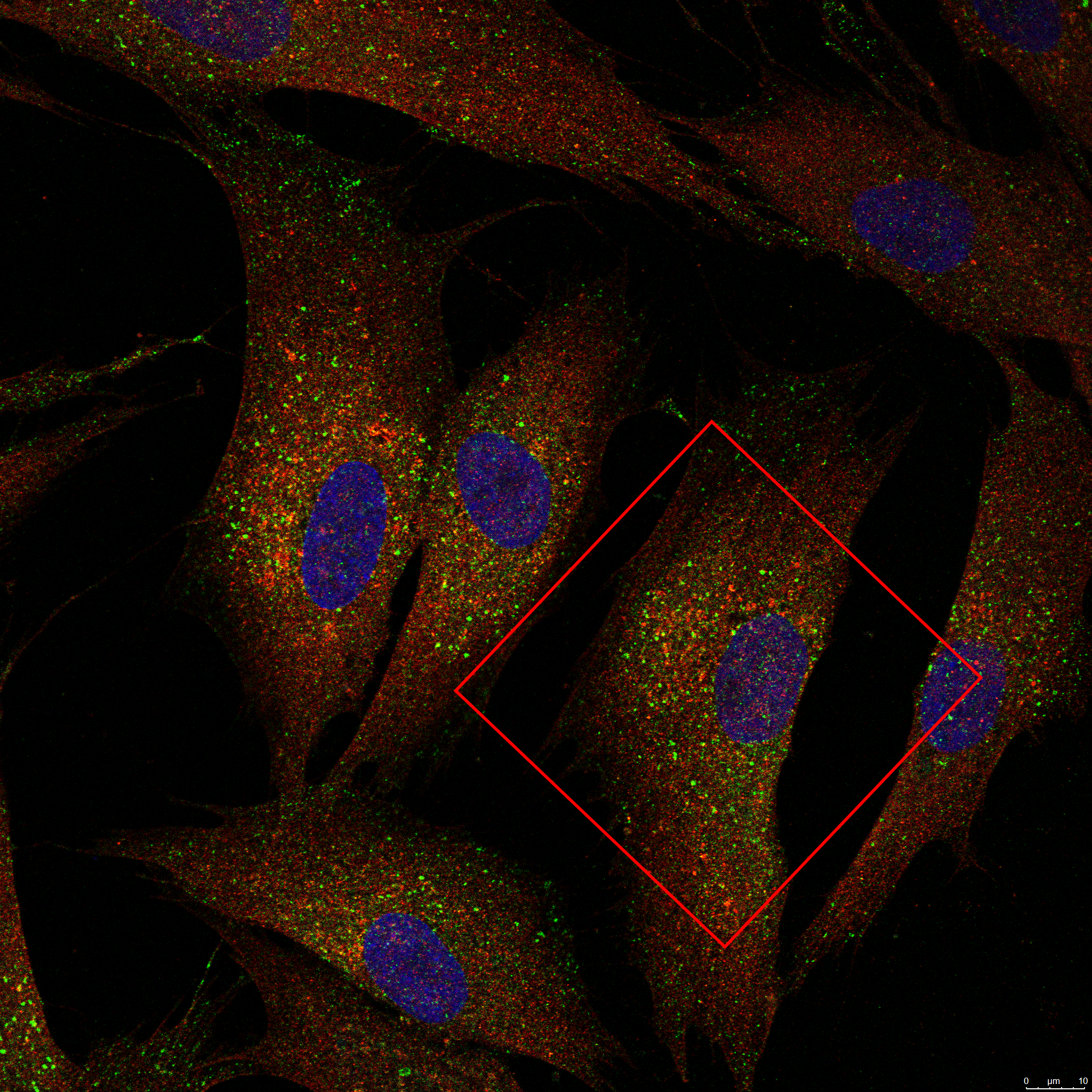

Supplement: Supplementary file 3 — Source Data Fig. 3 [file 44319_2023_45_MOESM3_ESM.zip › Fig 3/Fig 3D/F3D3 BJ LC3-R594, RAB5-M488_cGAMP 60'.tif]

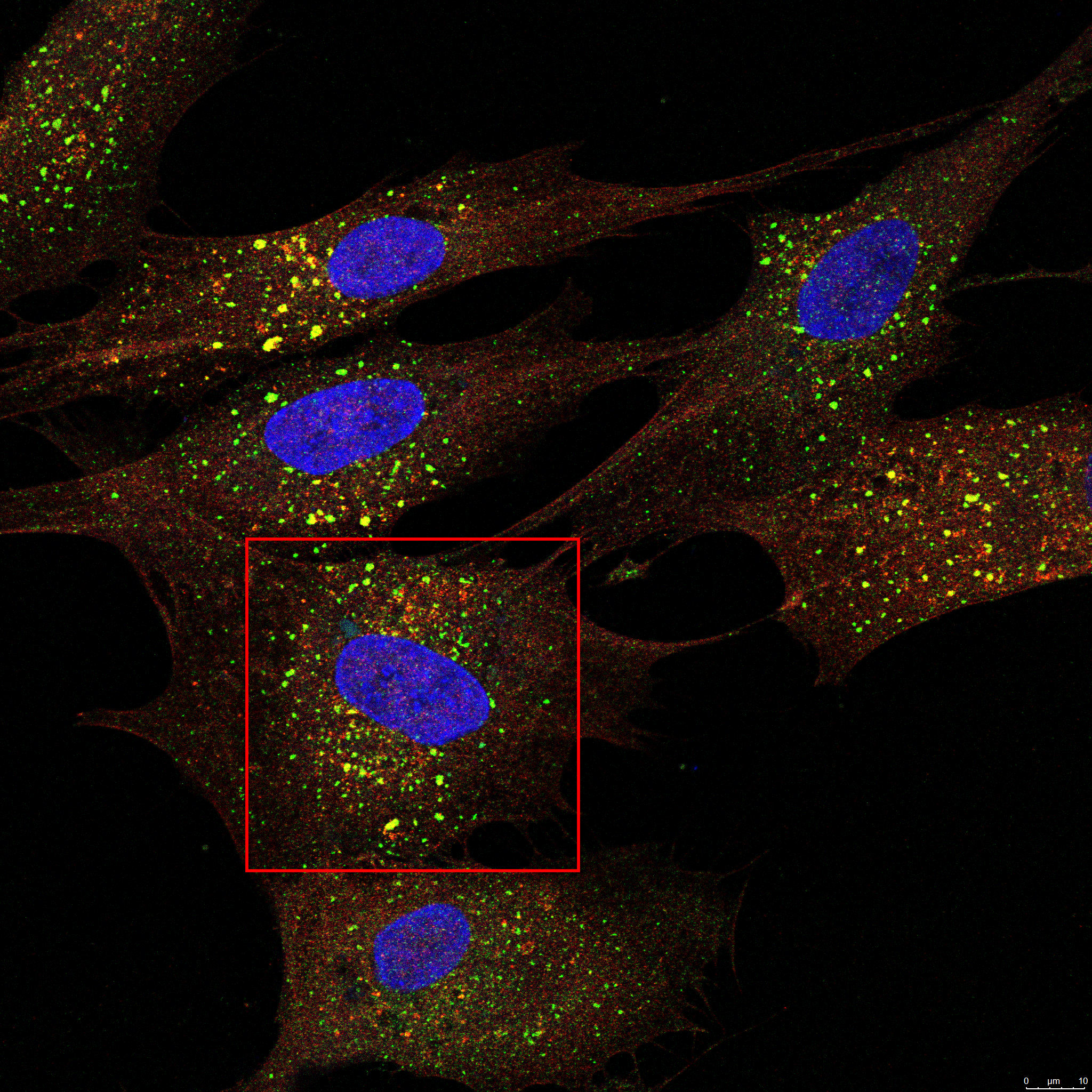

Supplement: Supplementary file 3 — Source Data Fig. 3 [file 44319_2023_45_MOESM3_ESM.zip › Fig 3/Fig 3D/F3D4 BJ LC3-R594, RAB5-M488_cGAMP 90'.tif]

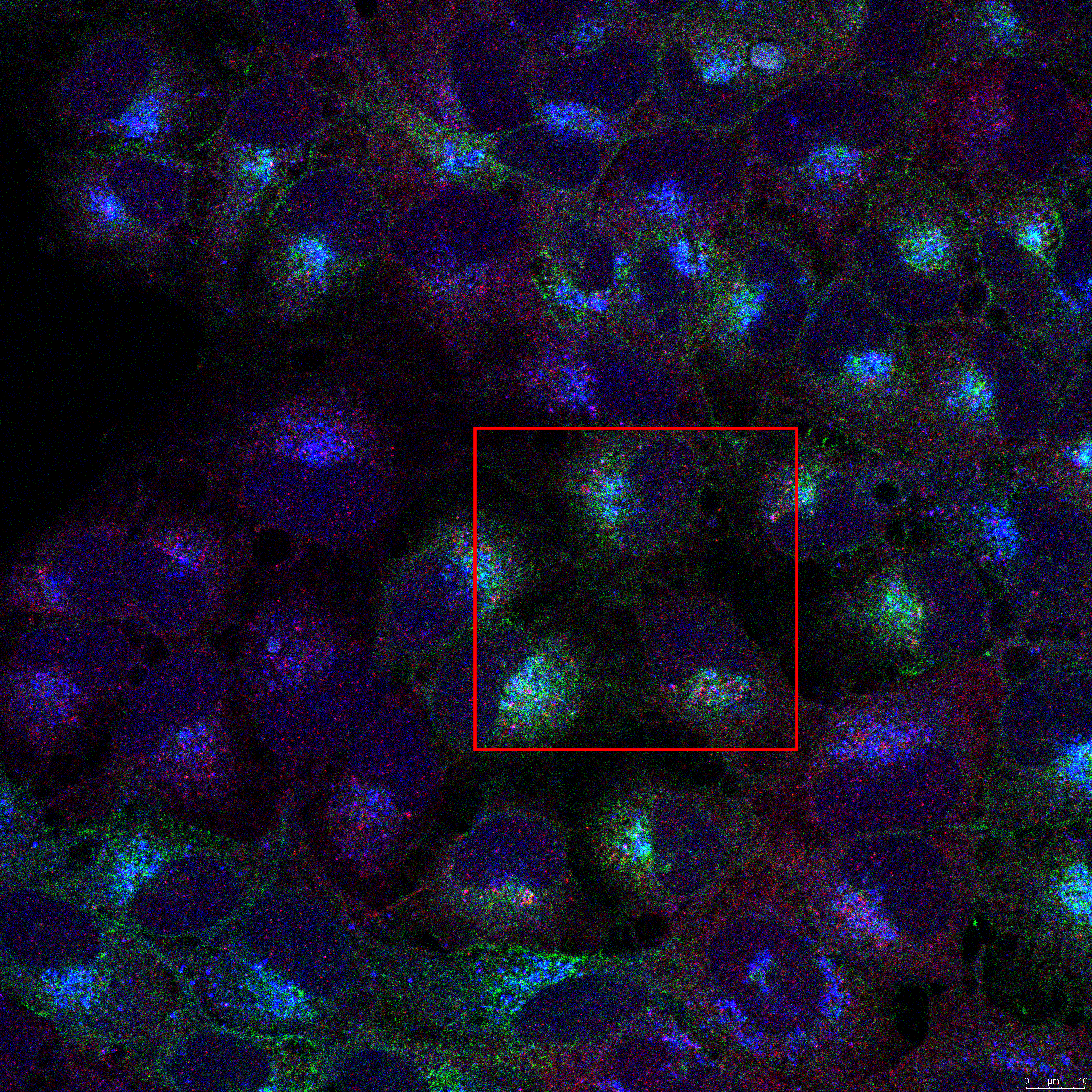

Supplement: Supplementary file 3 — Source Data Fig. 3 [file 44319_2023_45_MOESM3_ESM.zip › Fig 3/Fig 3I/F3I2 U2OS FST-blue, E-RAB11, LC3-R594_cGAMP 60'.tif]

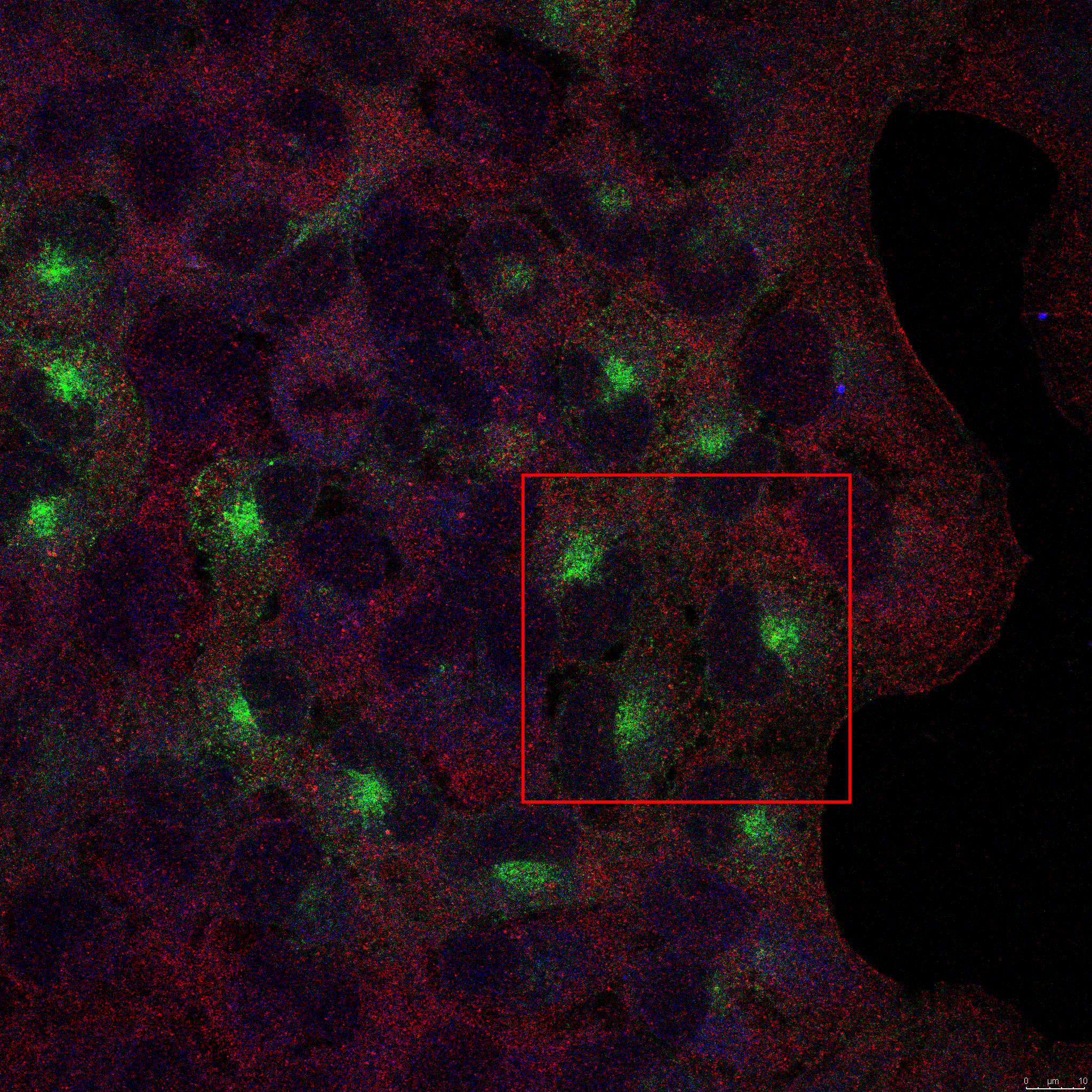

Supplement: Supplementary file 3 — Source Data Fig. 3 [file 44319_2023_45_MOESM3_ESM.zip › Fig 3/Fig 3I/F3I1 U2OS FST-Blue, E-RAB11, LC3-R594_cGAMP 0'.tif]

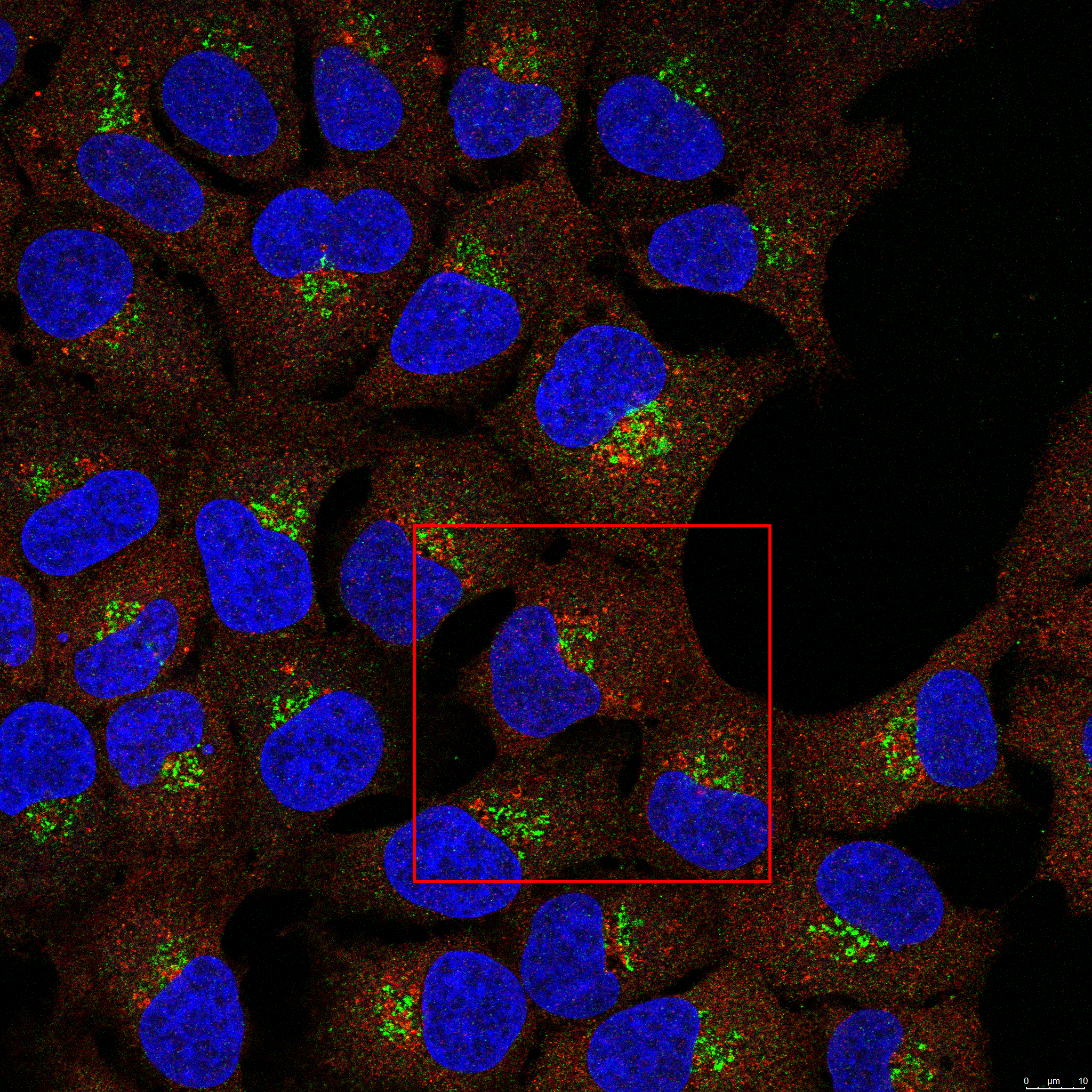

Supplement: Supplementary file 3 — Source Data Fig. 3 [file 44319_2023_45_MOESM3_ESM.zip › Fig 3/Fig 3A/F3A4.tif]

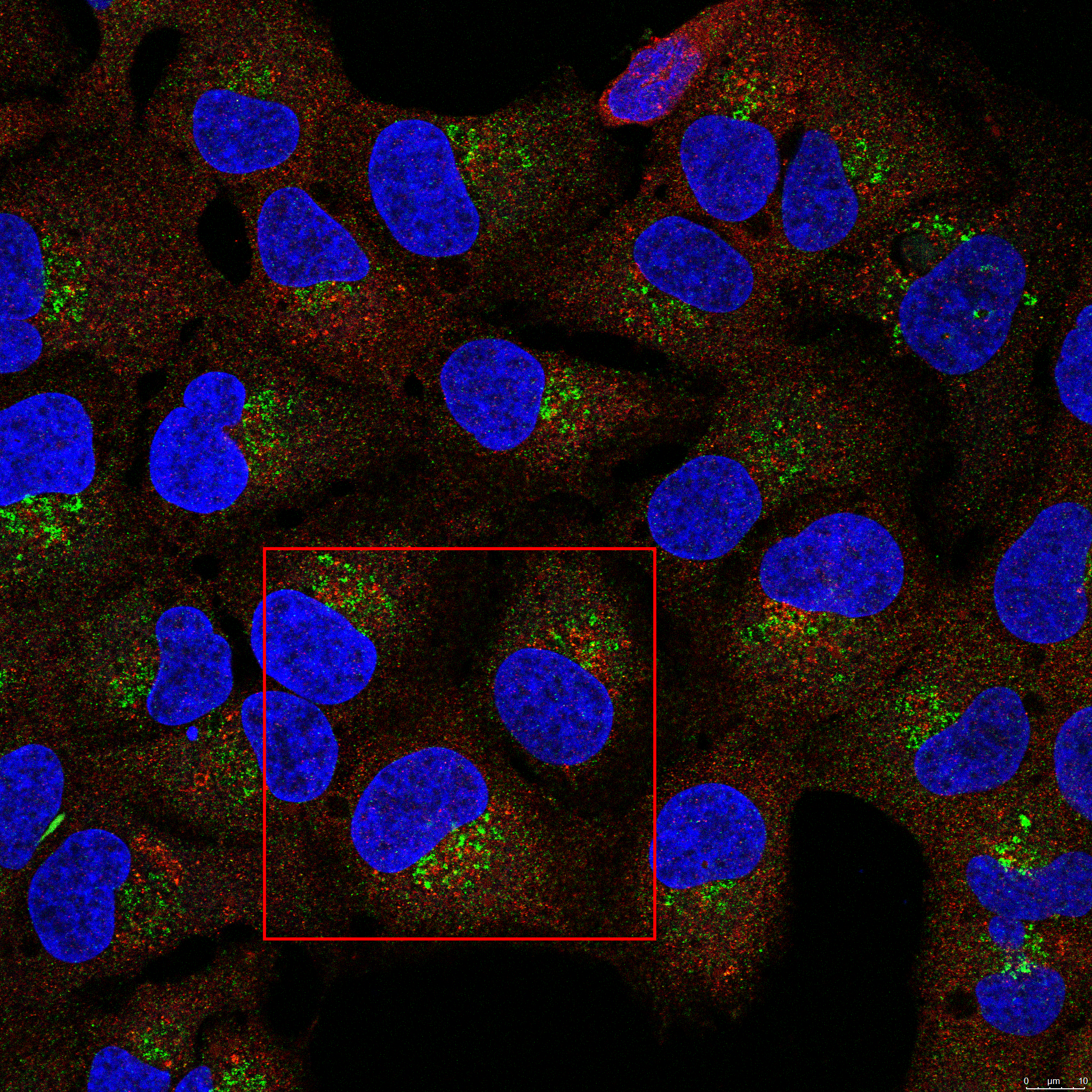

Supplement: Supplementary file 3 — Source Data Fig. 3 [file 44319_2023_45_MOESM3_ESM.zip › Fig 3/Fig 3A/F3A3.tif]

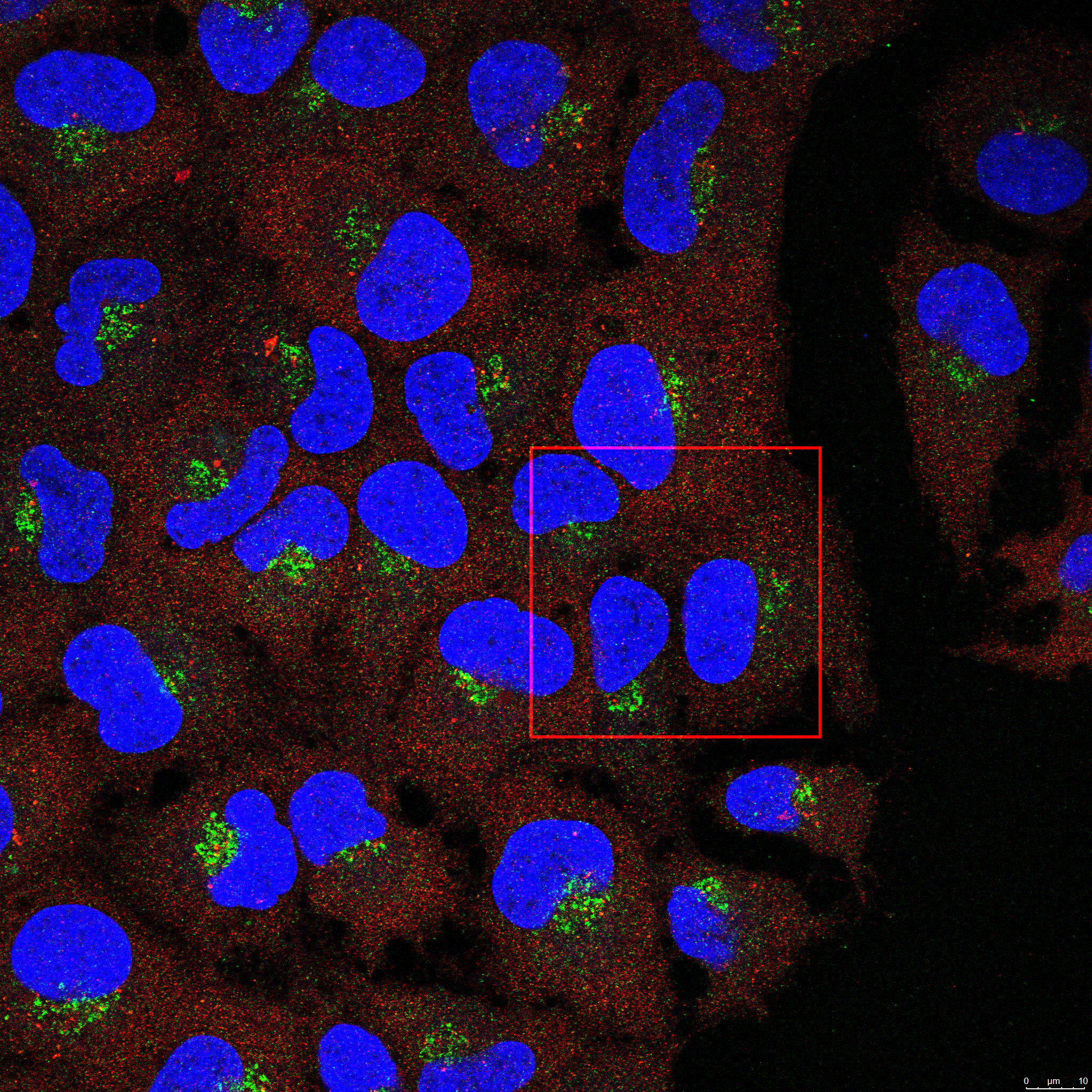

Supplement: Supplementary file 3 — Source Data Fig. 3 [file 44319_2023_45_MOESM3_ESM.zip › Fig 3/Fig 3A/F3A2.tif]

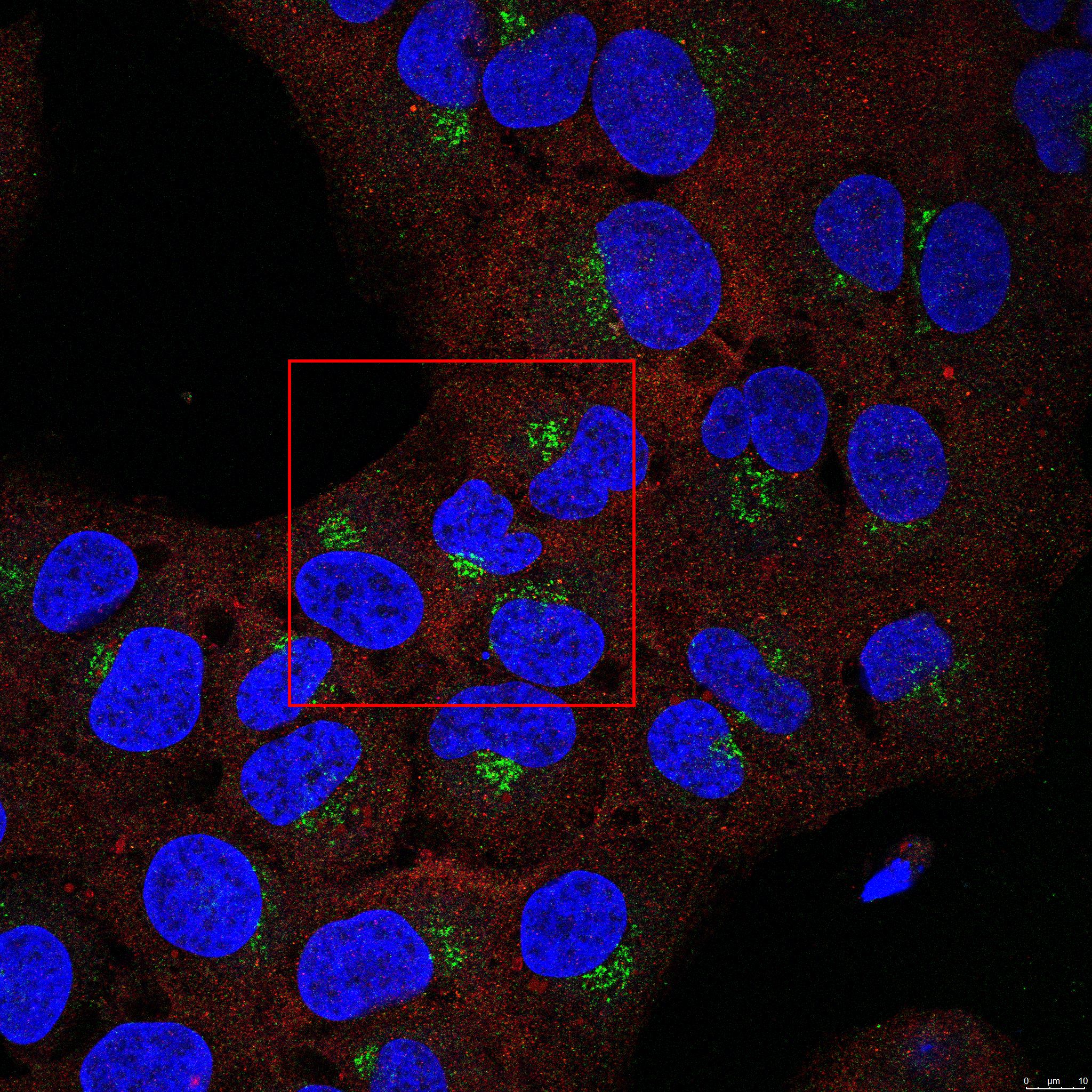

Supplement: Supplementary file 3 — Source Data Fig. 3 [file 44319_2023_45_MOESM3_ESM.zip › Fig 3/Fig 3A/F3A1.tif]

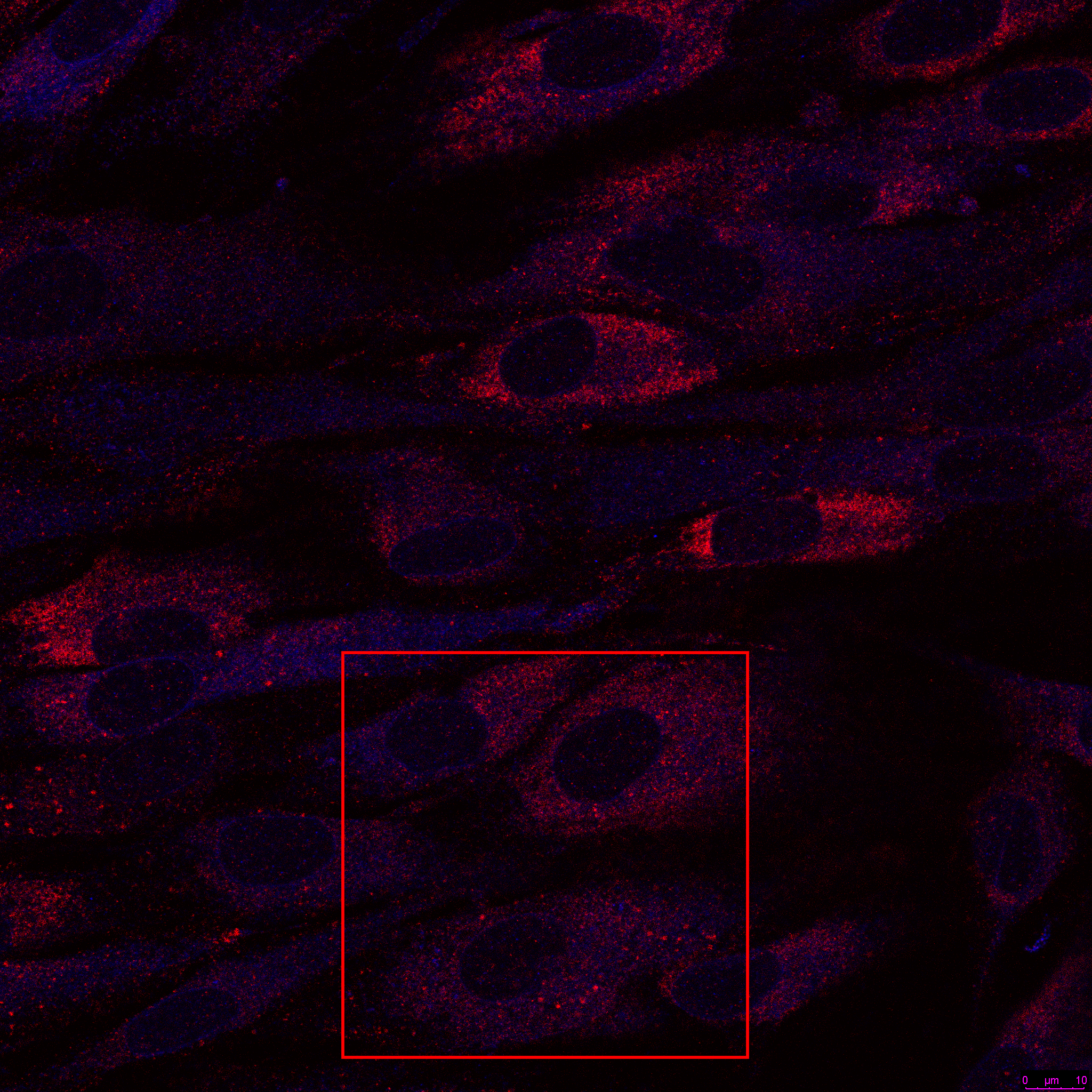

Supplement: Supplementary file 3 — Source Data Fig. 3 [file 44319_2023_45_MOESM3_ESM.zip › Fig 3/Fig 3F/F3F1 BJ ST-blue RAB5-594_cGAMP-0min.tif]

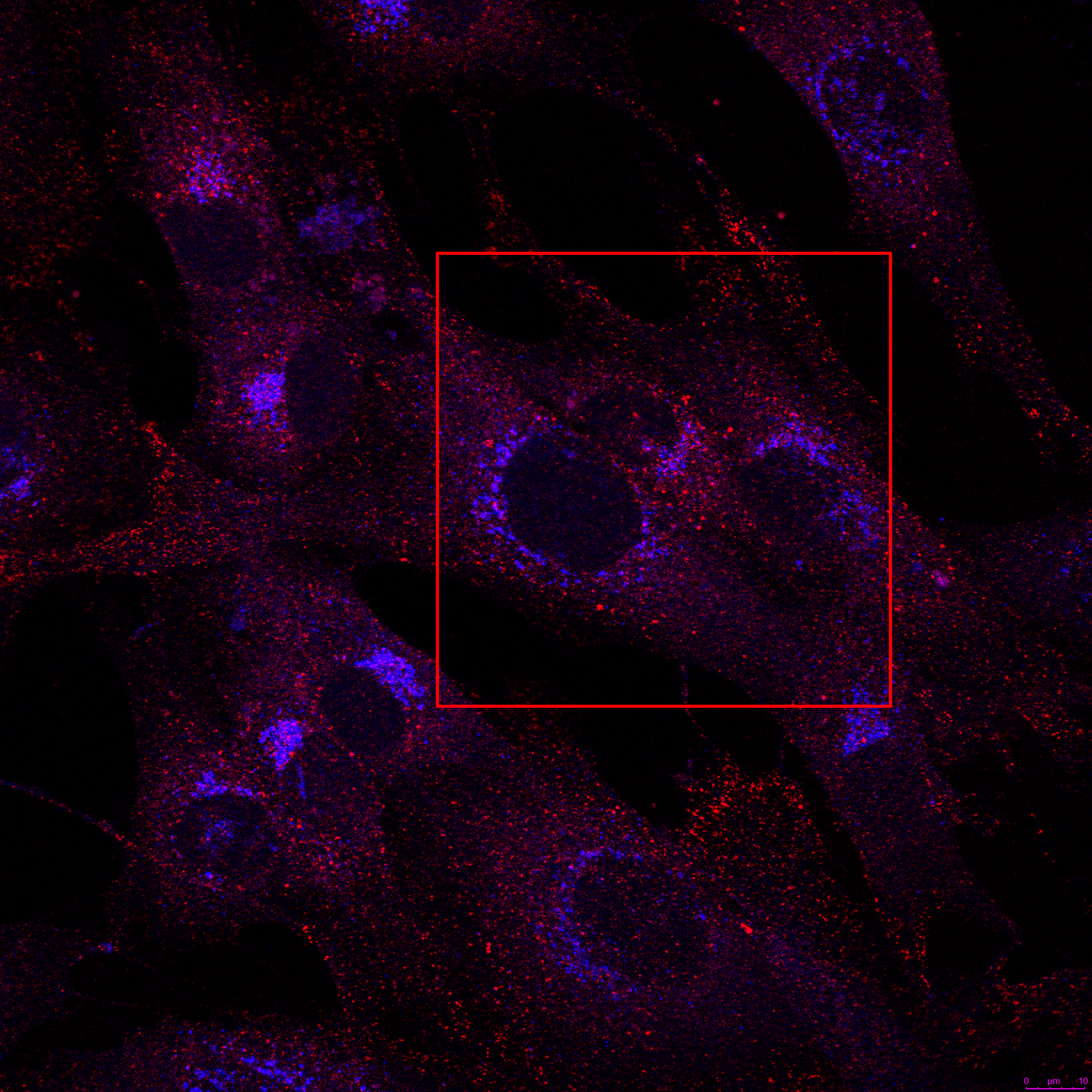

Supplement: Supplementary file 3 — Source Data Fig. 3 [file 44319_2023_45_MOESM3_ESM.zip › Fig 3/Fig 3F/F3F3 BJ ST-blue RAB5-594_cGAMP-60min.tif]

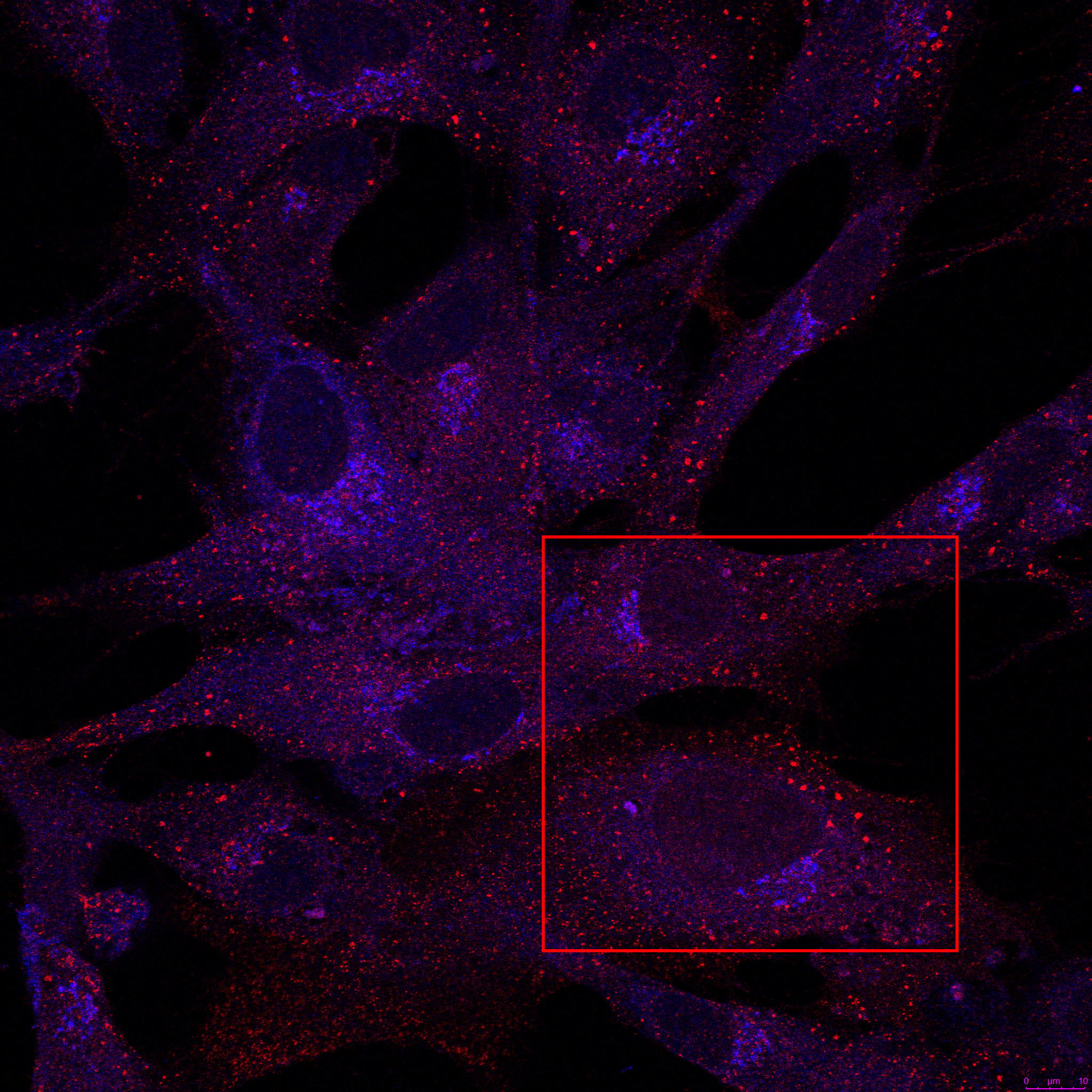

Supplement: Supplementary file 3 — Source Data Fig. 3 [file 44319_2023_45_MOESM3_ESM.zip › Fig 3/Fig 3F/F3F2 BJ ST-blue RAB5-594_cGAMP-30min.tif]

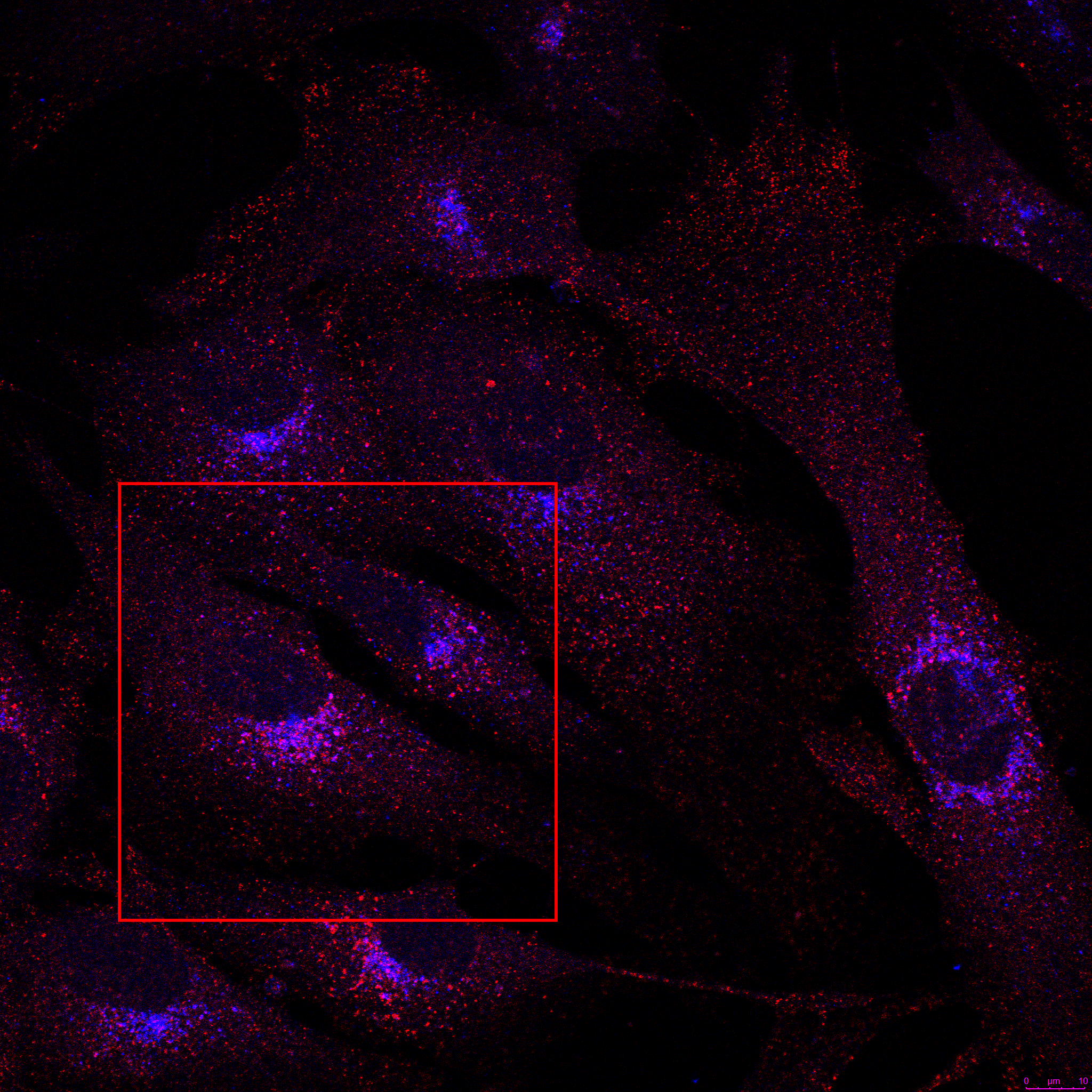

Supplement: Supplementary file 3 — Source Data Fig. 3 [file 44319_2023_45_MOESM3_ESM.zip › Fig 3/Fig 3F/F3F4 BJ ST-blue RAB5-594_cGAMP-90min.tif]

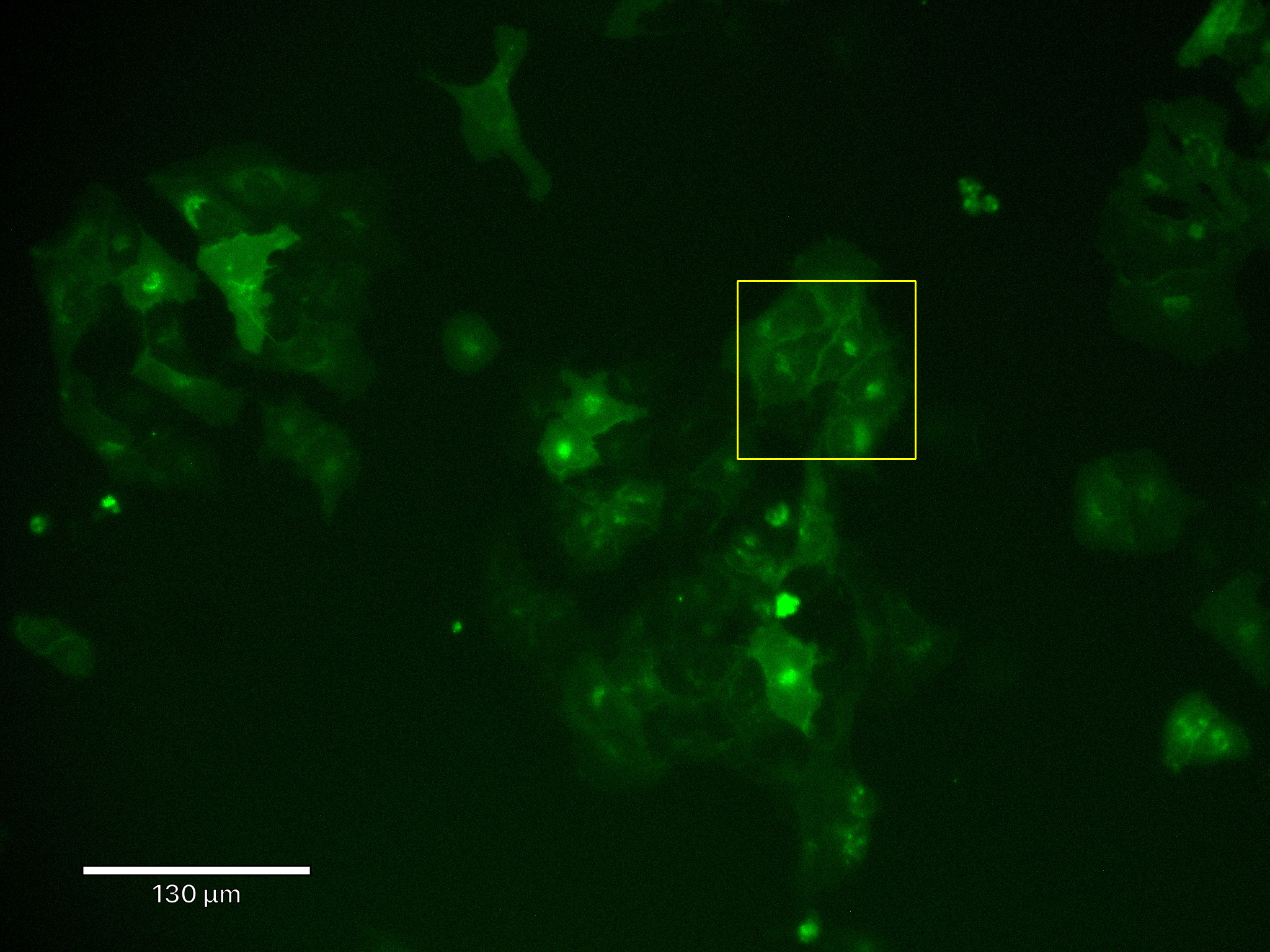

Supplement: Supplementary file 4 — Source Data Fig. 4 [file 44319_2023_45_MOESM4_ESM.zip › Fig 4/Fig 4C/F4C5_mST+ DMXAA 1h.png]

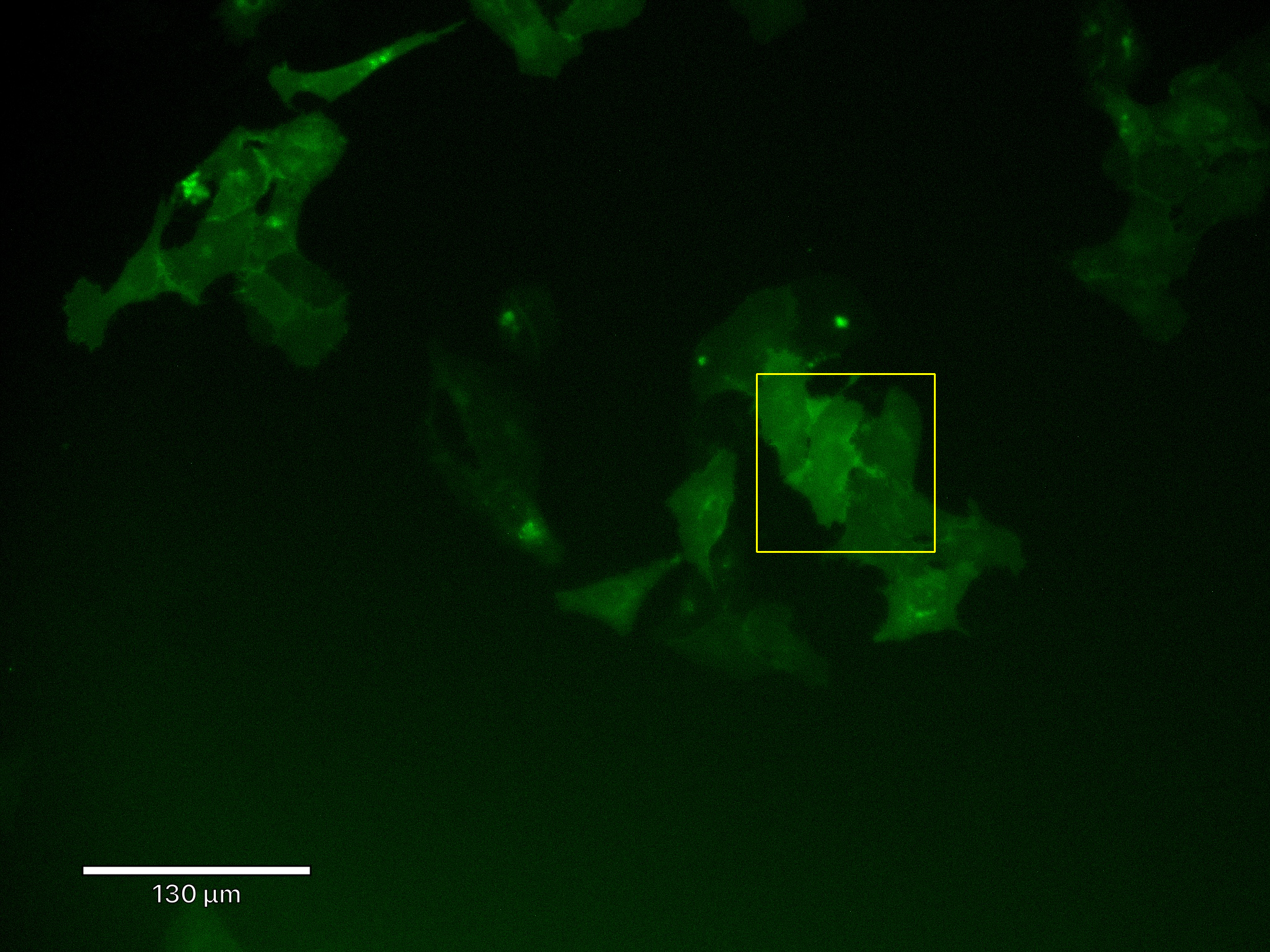

Supplement: Supplementary file 4 — Source Data Fig. 4 [file 44319_2023_45_MOESM4_ESM.zip › Fig 4/Fig 4C/F4C4_mST+ DMXAA 0h.png]

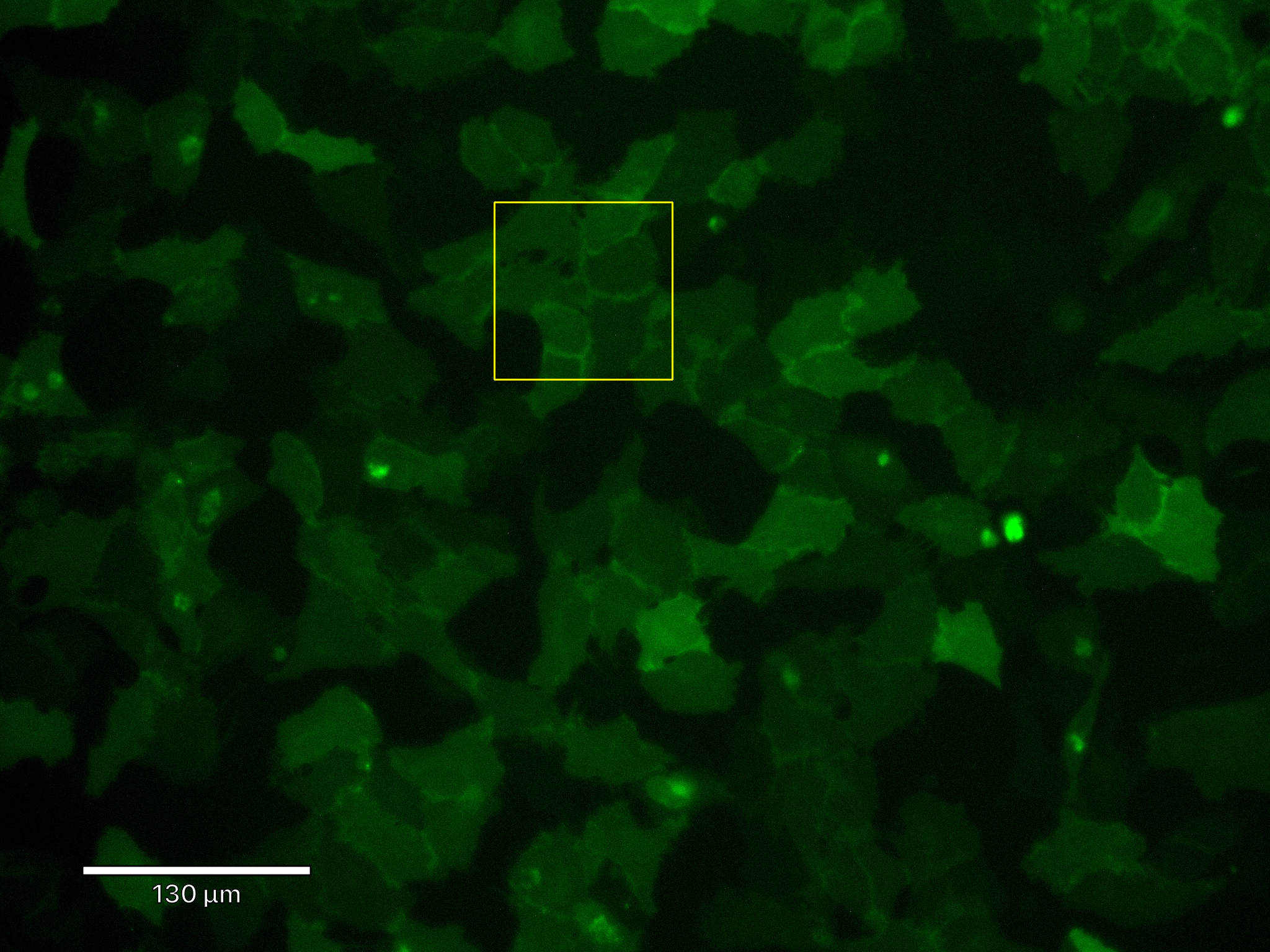

Supplement: Supplementary file 4 — Source Data Fig. 4 [file 44319_2023_45_MOESM4_ESM.zip › Fig 4/Fig 4C/F4C3_EV + DMXAA 4h.png]

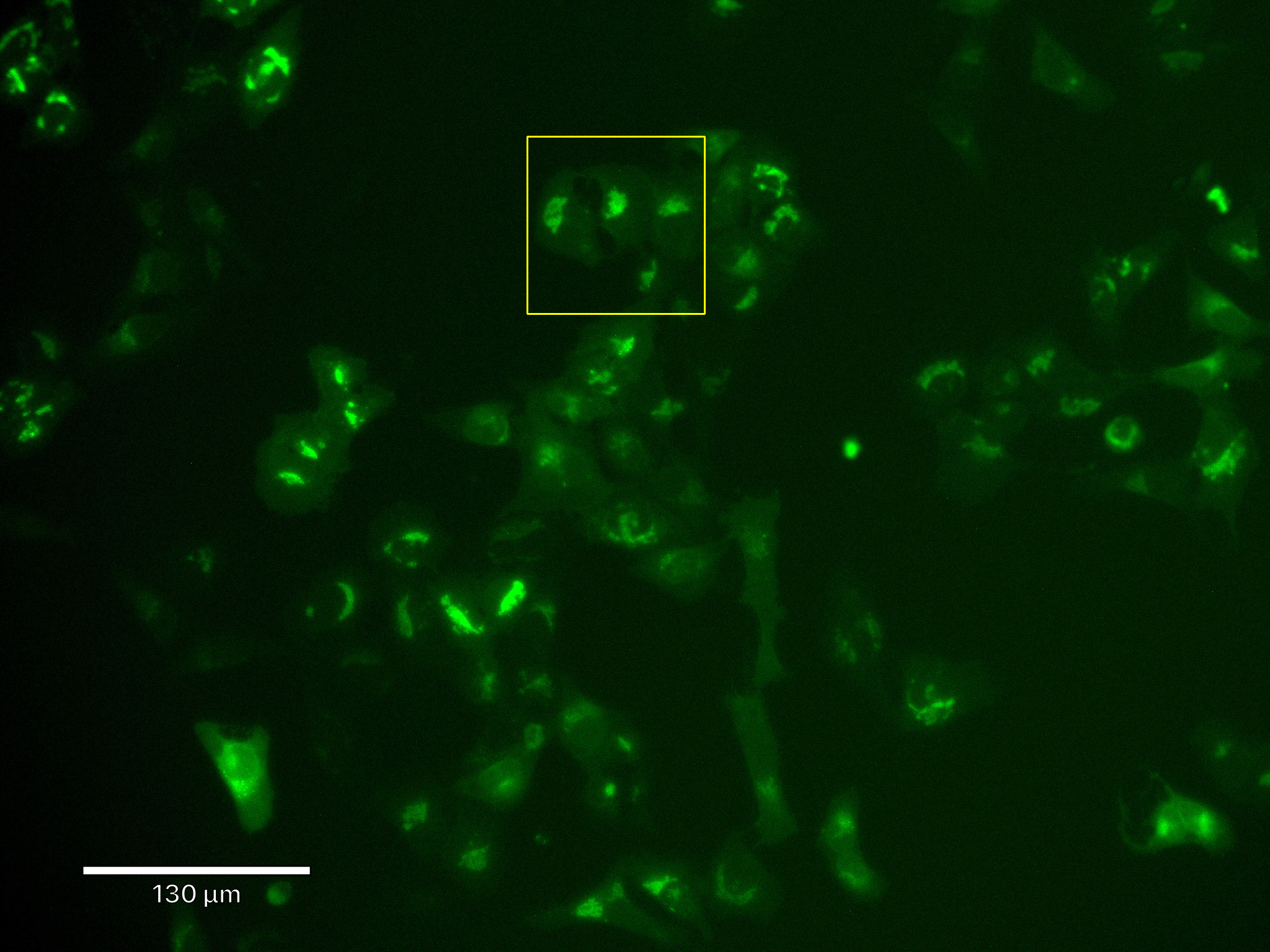

Supplement: Supplementary file 4 — Source Data Fig. 4 [file 44319_2023_45_MOESM4_ESM.zip › Fig 4/Fig 4C/F4C6_mST+ DMXAA 4h.png]

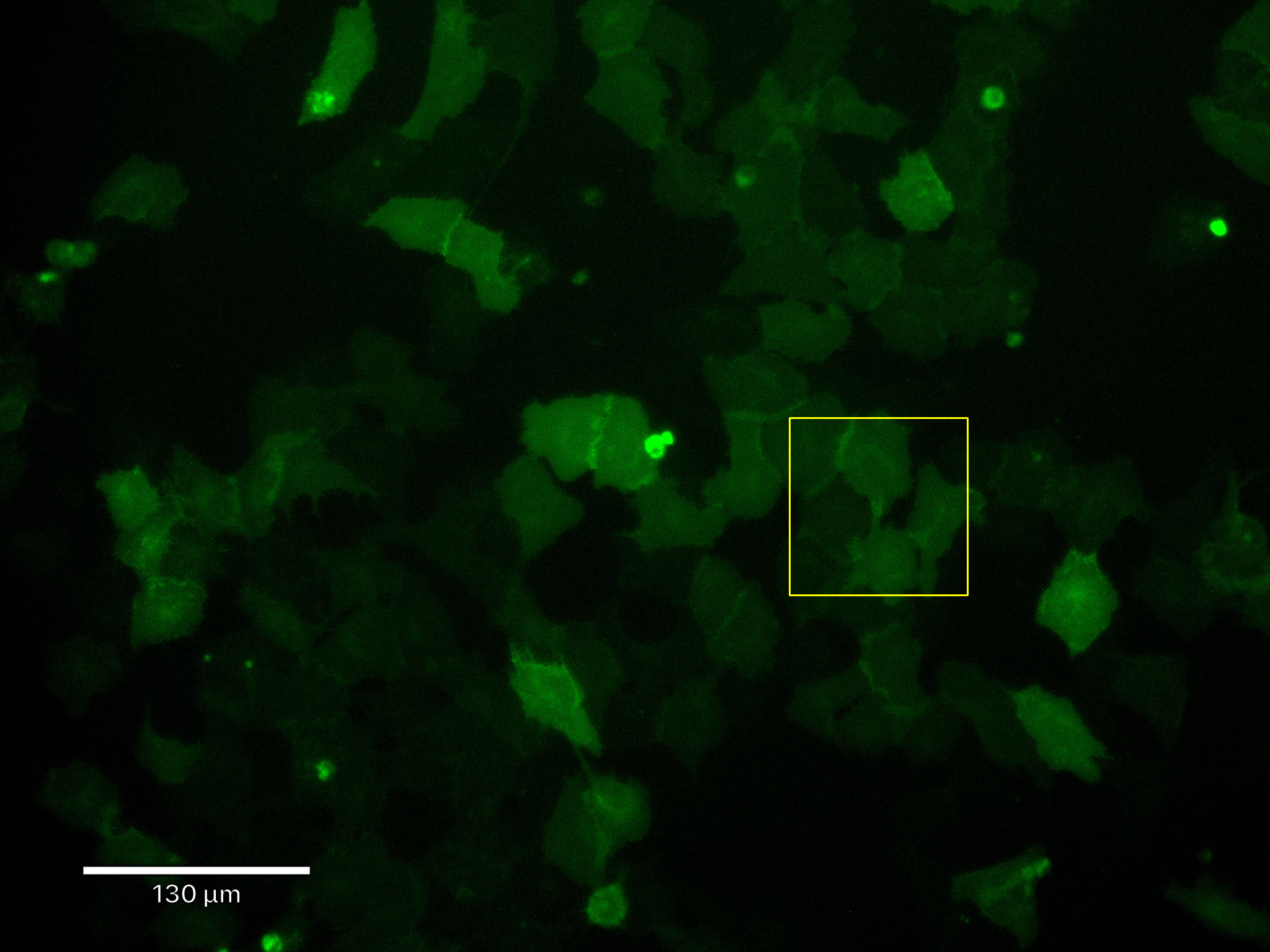

Supplement: Supplementary file 4 — Source Data Fig. 4 [file 44319_2023_45_MOESM4_ESM.zip › Fig 4/Fig 4C/F4C1_EV + DMXAA 0h.png]

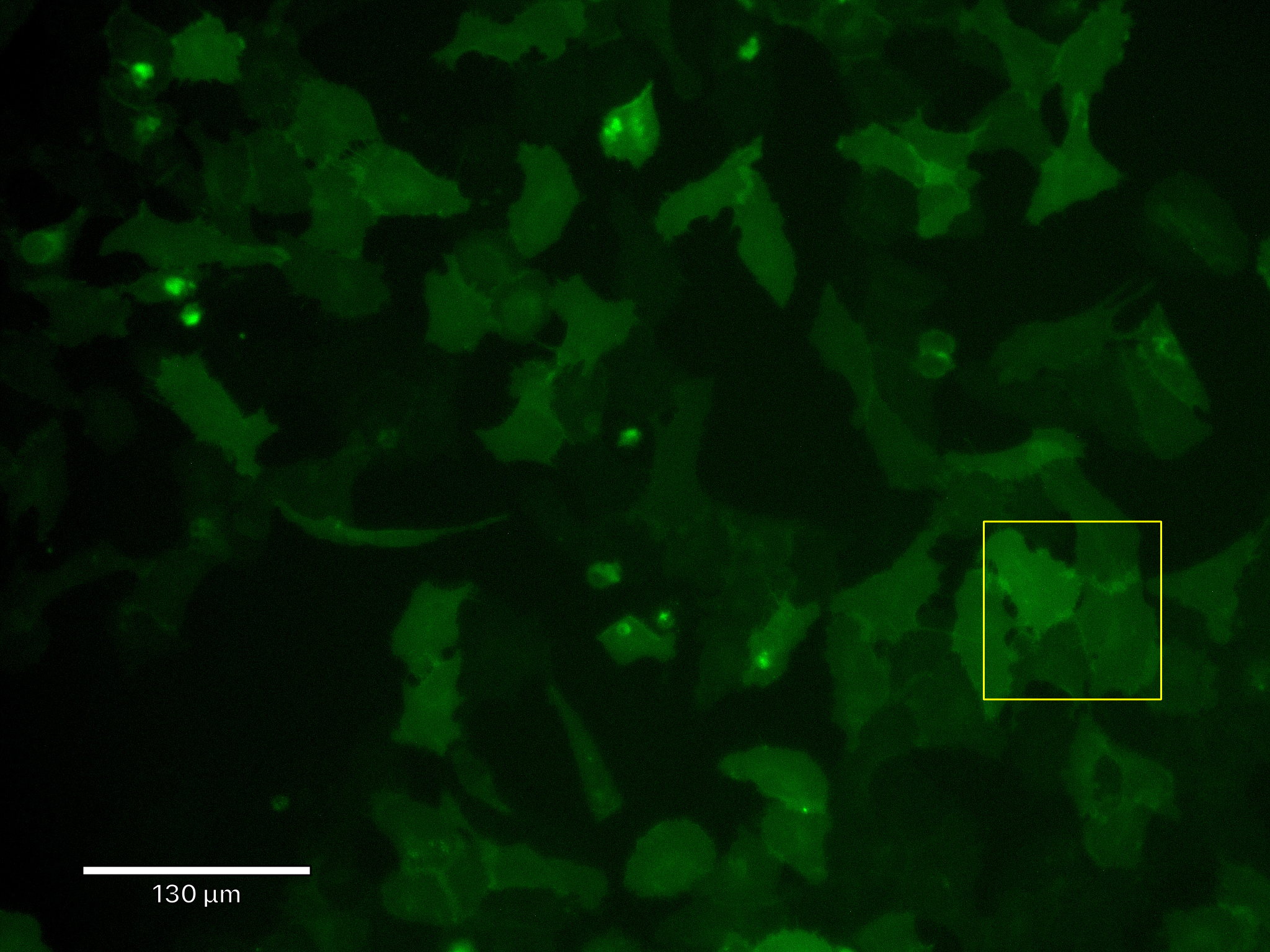

Supplement: Supplementary file 4 — Source Data Fig. 4 [file 44319_2023_45_MOESM4_ESM.zip › Fig 4/Fig 4C/F4C2_EV + DMXAA 1h.png]

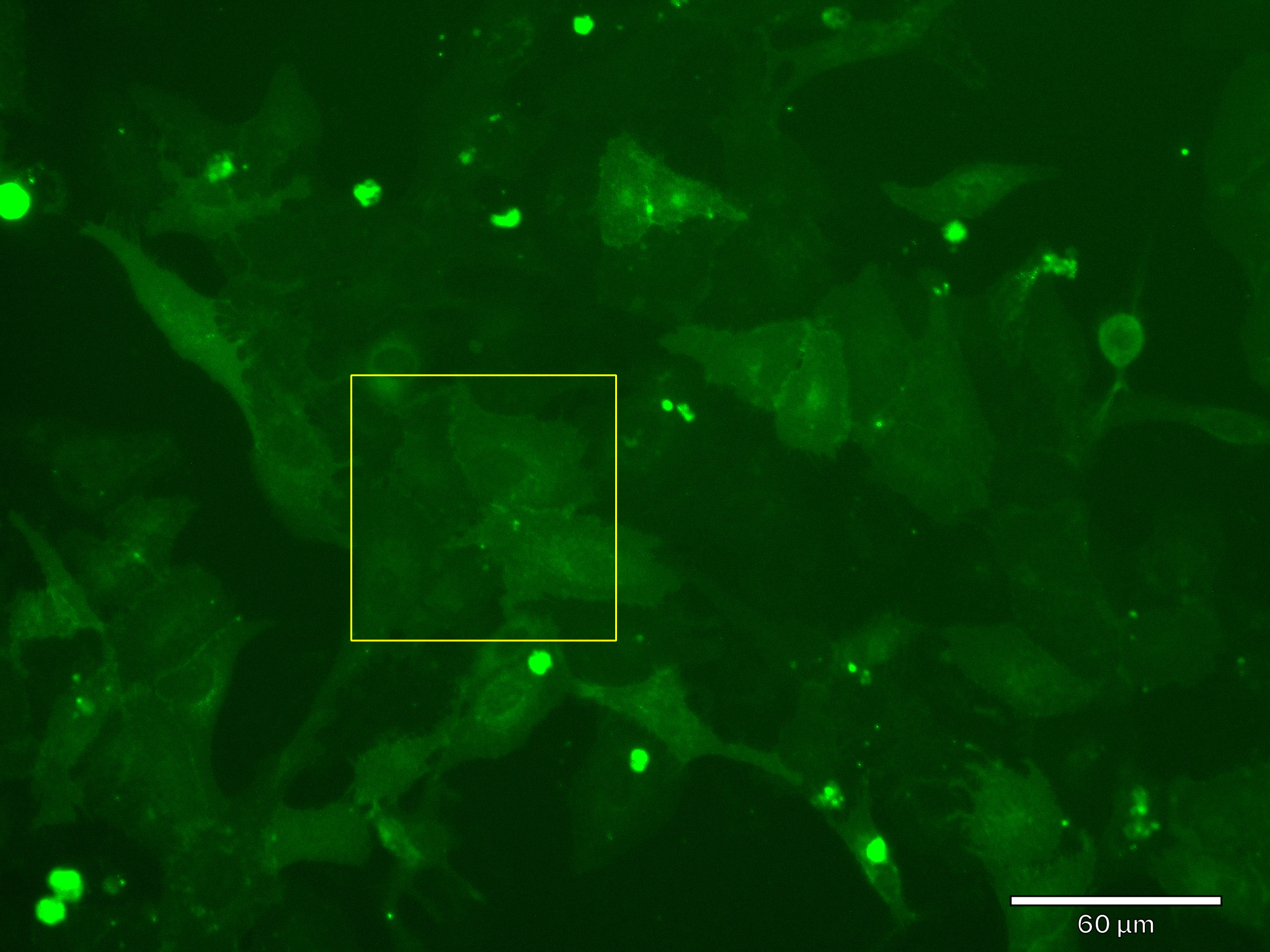

Supplement: Supplementary file 4 — Source Data Fig. 4 [file 44319_2023_45_MOESM4_ESM.zip › Fig 4/Fig 4E/F4E1_- cGAMP.png]

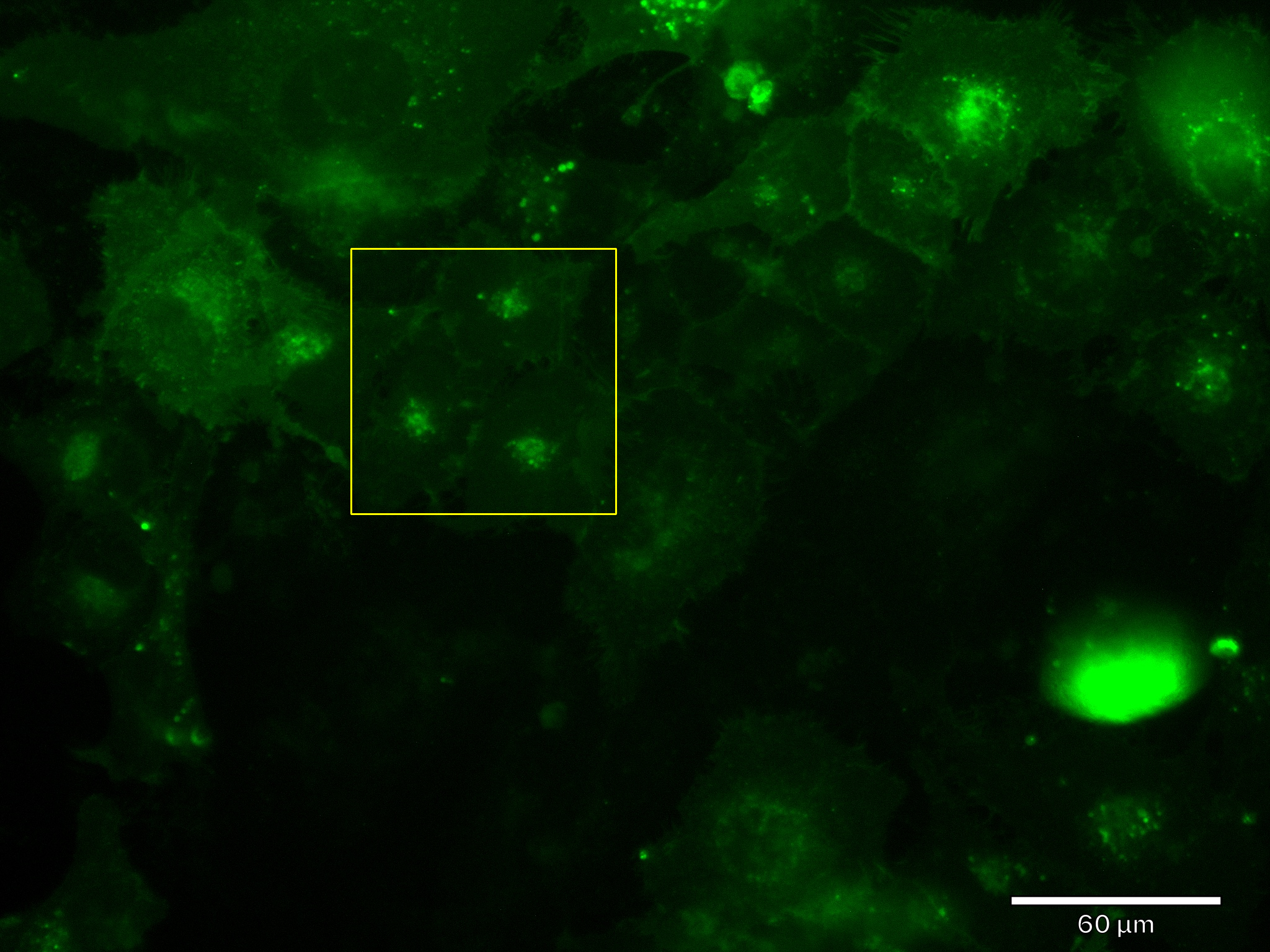

Supplement: Supplementary file 4 — Source Data Fig. 4 [file 44319_2023_45_MOESM4_ESM.zip › Fig 4/Fig 4E/F4E2_+ cGAMP.png]

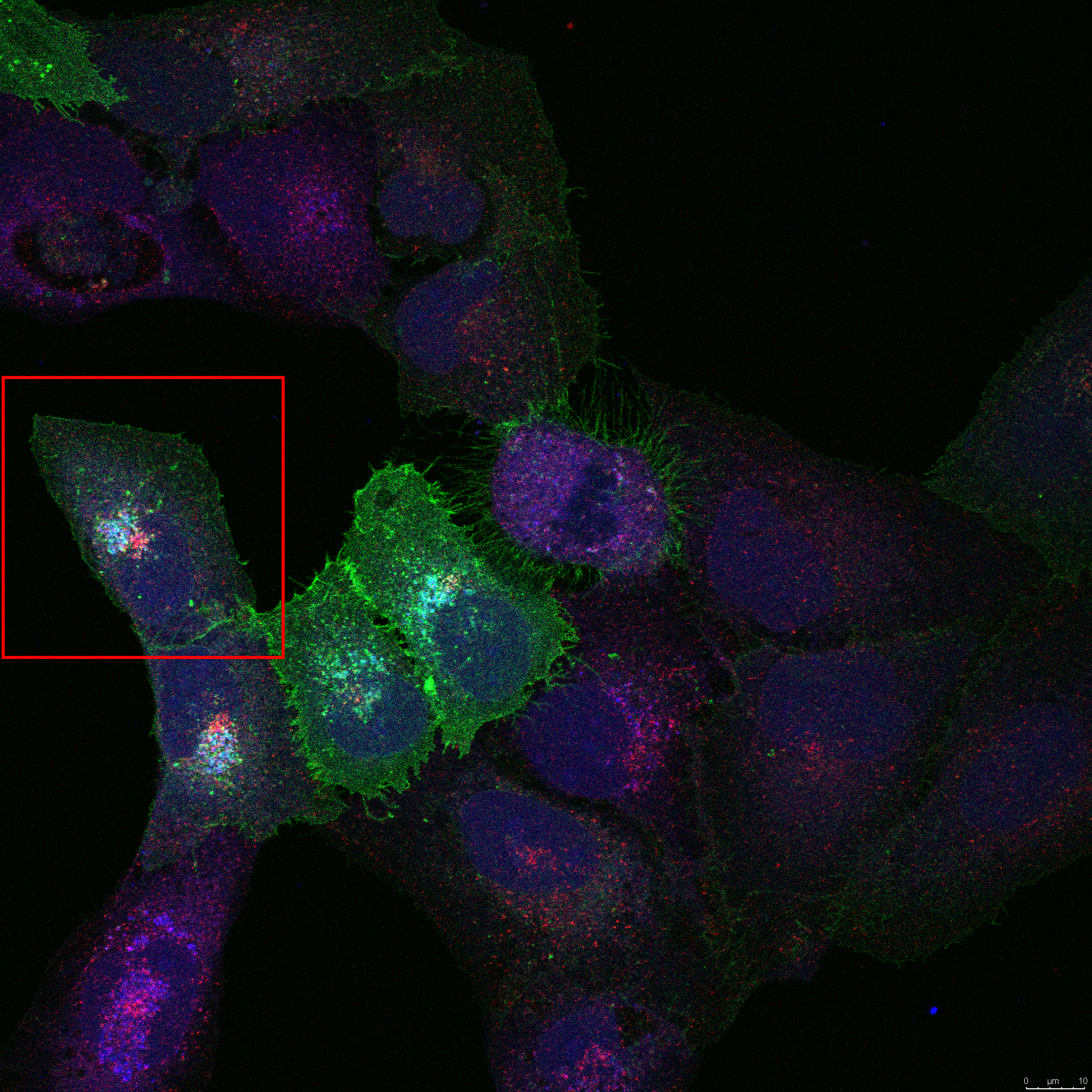

Supplement: Supplementary file 4 — Source Data Fig. 4 [file 44319_2023_45_MOESM4_ESM.zip › Fig 4/Fig 4K/F4K u2os FmST lysoph, fstblue, rab5-594_DMXAA_90MIN.tif]

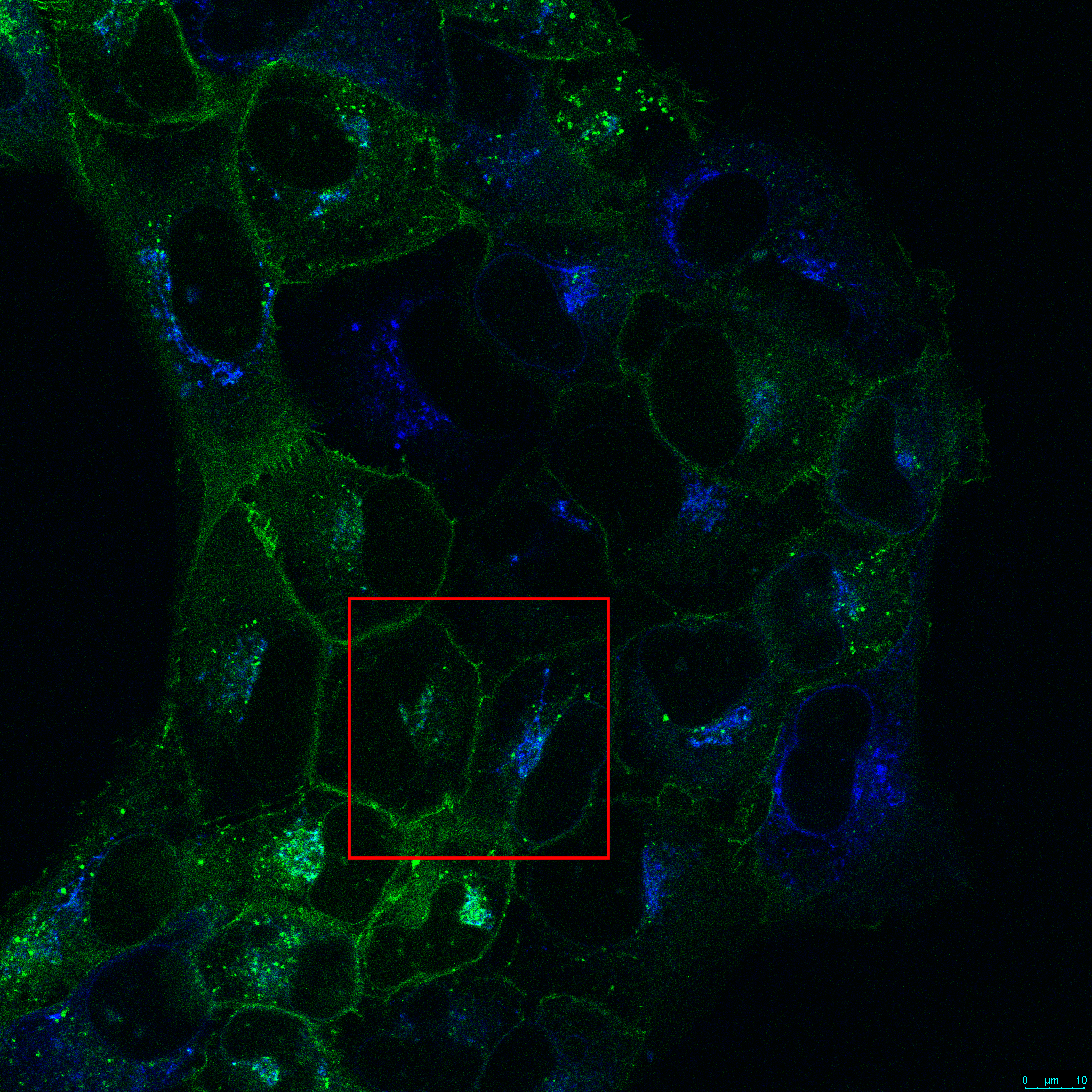

Supplement: Supplementary file 4 — Source Data Fig. 4 [file 44319_2023_45_MOESM4_ESM.zip › Fig 4/Fig 4I/F4I2 U2OS lysoPH FST-blue_DMXAA-30min.tif]

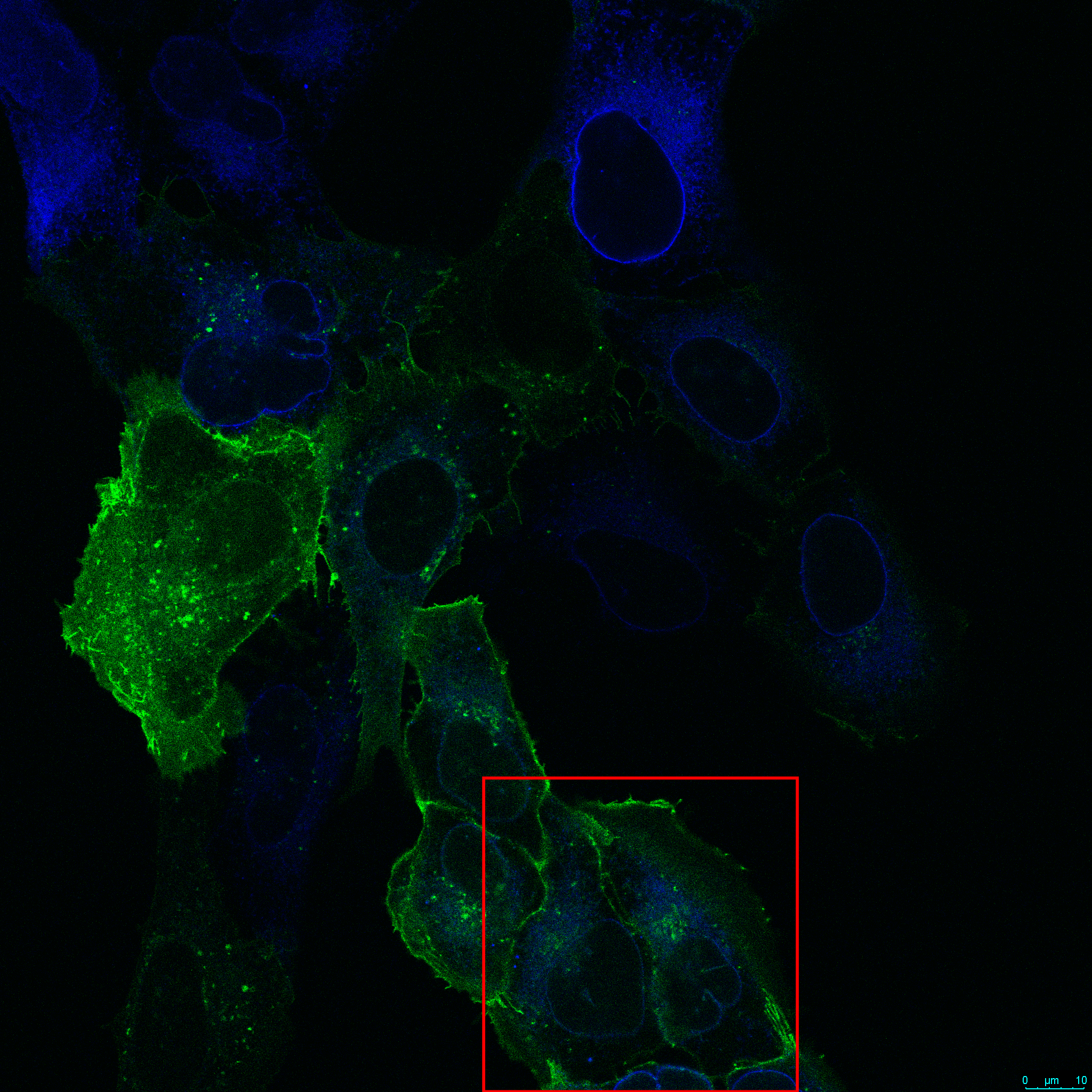

Supplement: Supplementary file 4 — Source Data Fig. 4 [file 44319_2023_45_MOESM4_ESM.zip › Fig 4/Fig 4I/F4I1 U2OS lysoPH FST-blue_DMXAA-0min.tif]

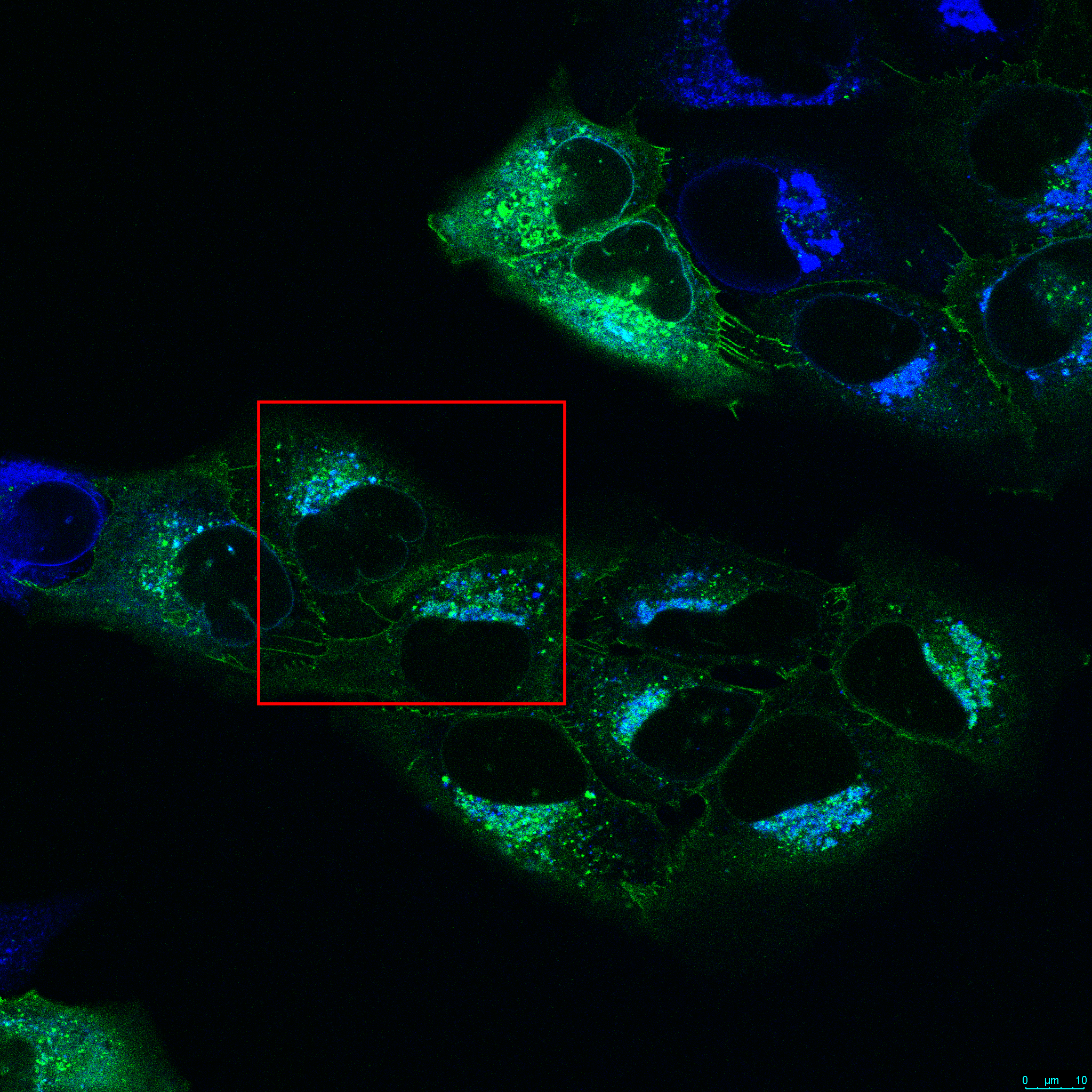

Supplement: Supplementary file 4 — Source Data Fig. 4 [file 44319_2023_45_MOESM4_ESM.zip › Fig 4/Fig 4I/F4I3 U2OS lysoPH FST-blue_DMXAA-120min.tif]

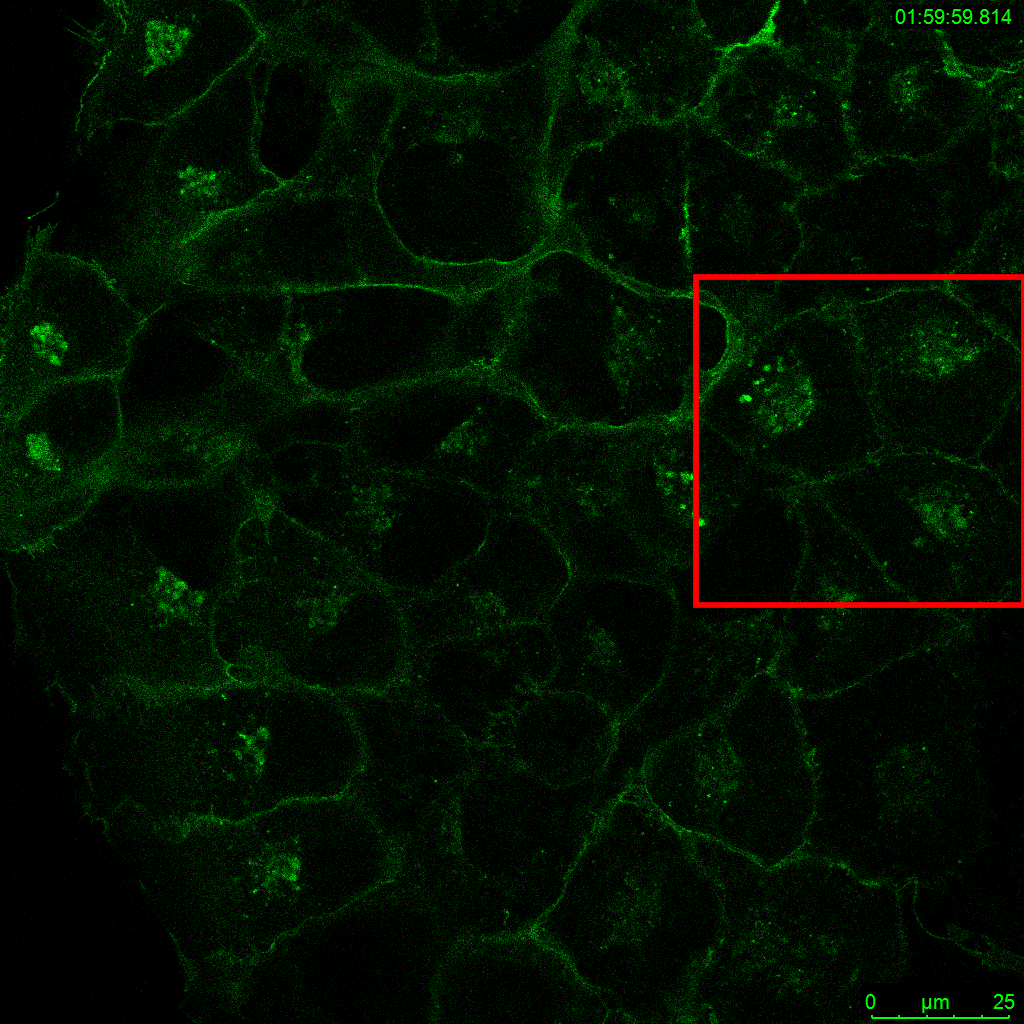

Supplement: Supplementary file 4 — Source Data Fig. 4 [file 44319_2023_45_MOESM4_ESM.zip › Fig 4/Fig 4G/F4G3 120min.tif]

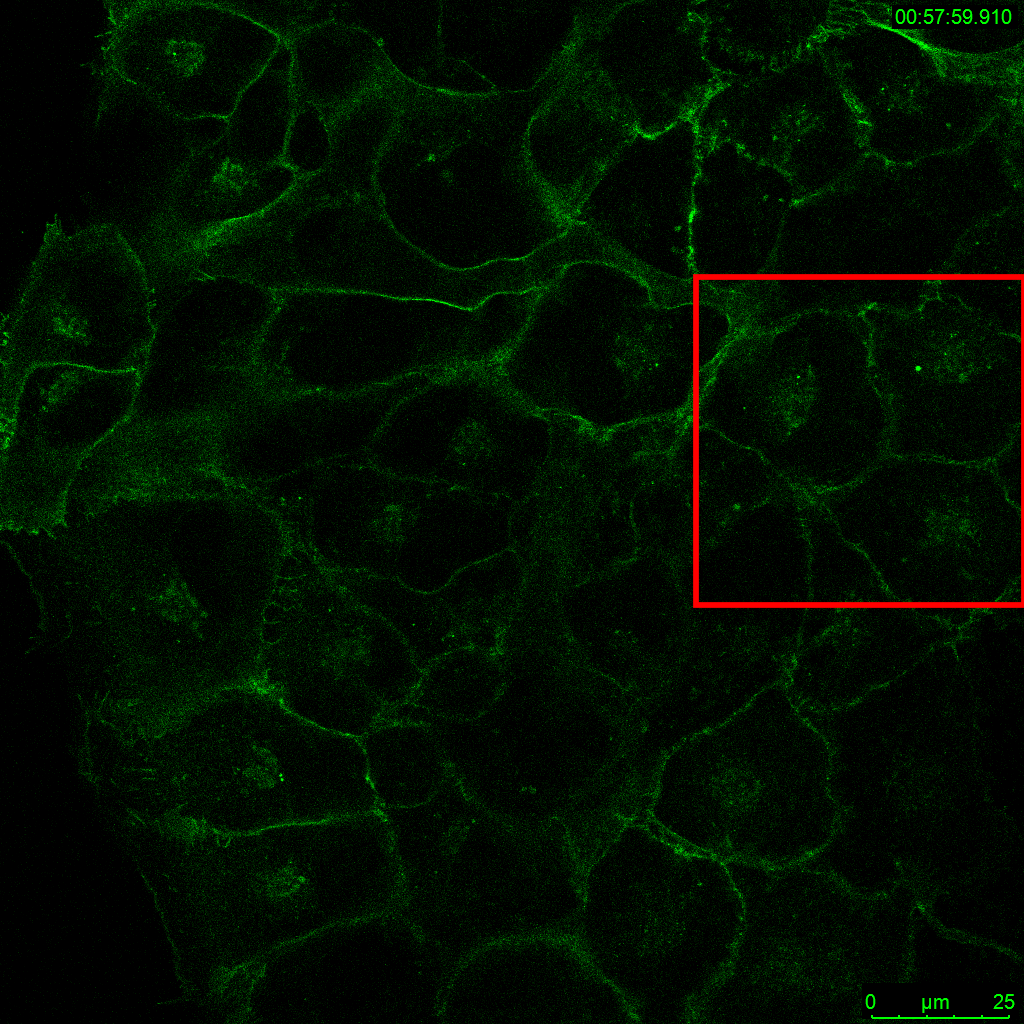

Supplement: Supplementary file 4 — Source Data Fig. 4 [file 44319_2023_45_MOESM4_ESM.zip › Fig 4/Fig 4G/F4G3 60min.tif]

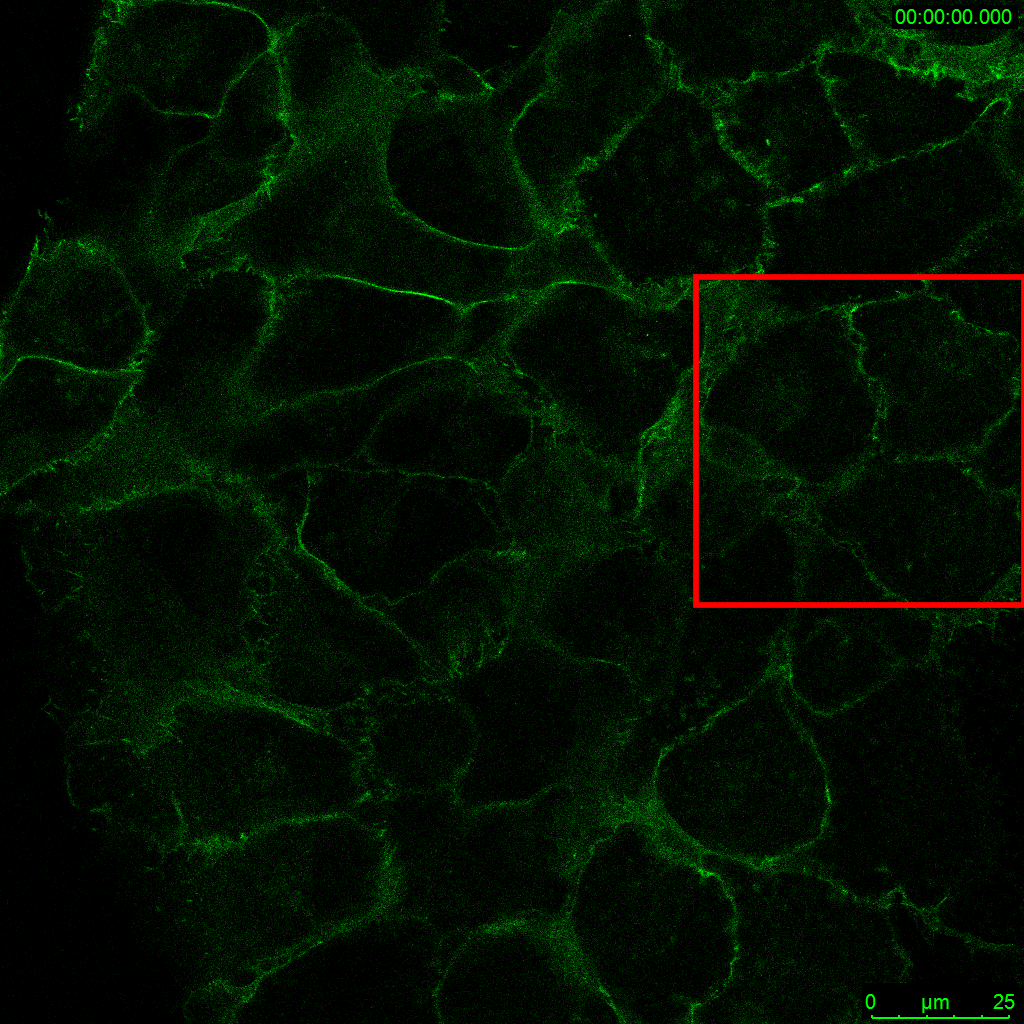

Supplement: Supplementary file 4 — Source Data Fig. 4 [file 44319_2023_45_MOESM4_ESM.zip › Fig 4/Fig 4G/F4G1 0min.tif]

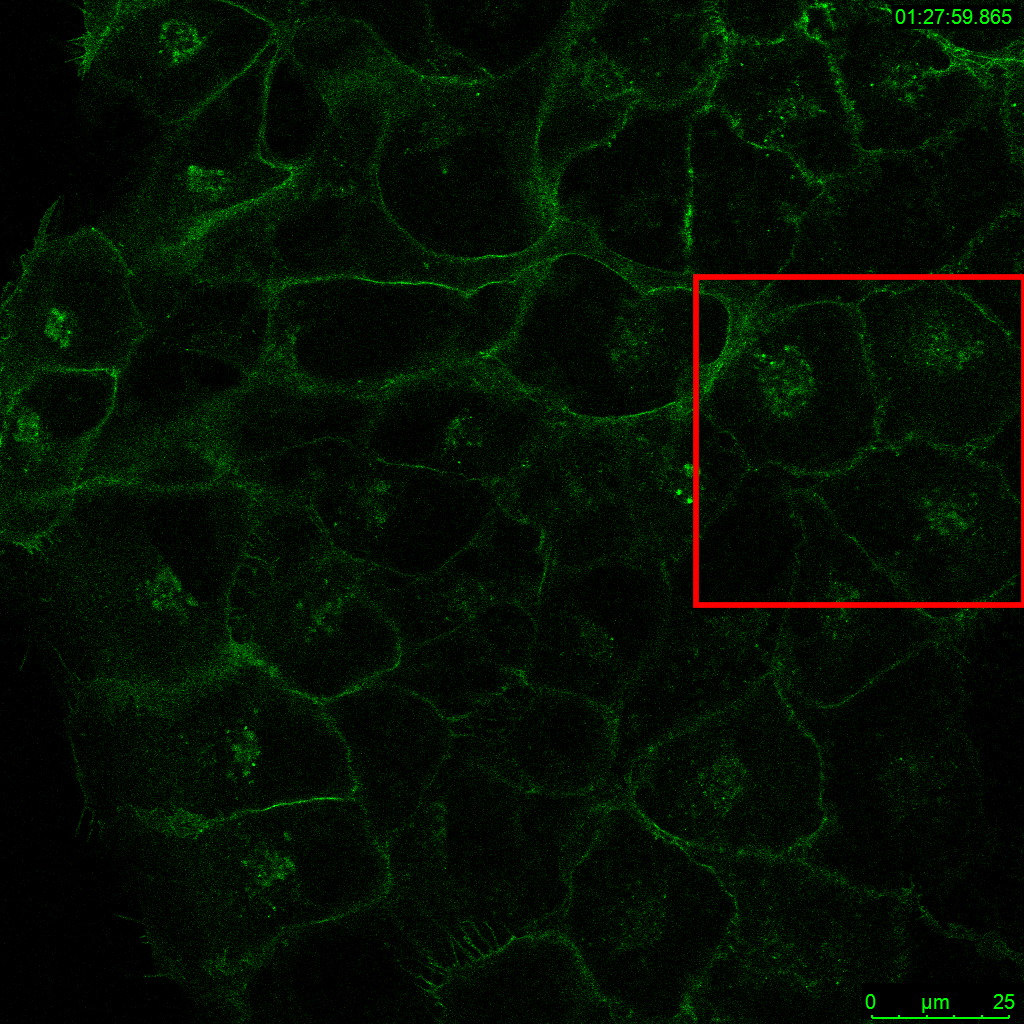

Supplement: Supplementary file 4 — Source Data Fig. 4 [file 44319_2023_45_MOESM4_ESM.zip › Fig 4/Fig 4G/F4G3 90min.tif]

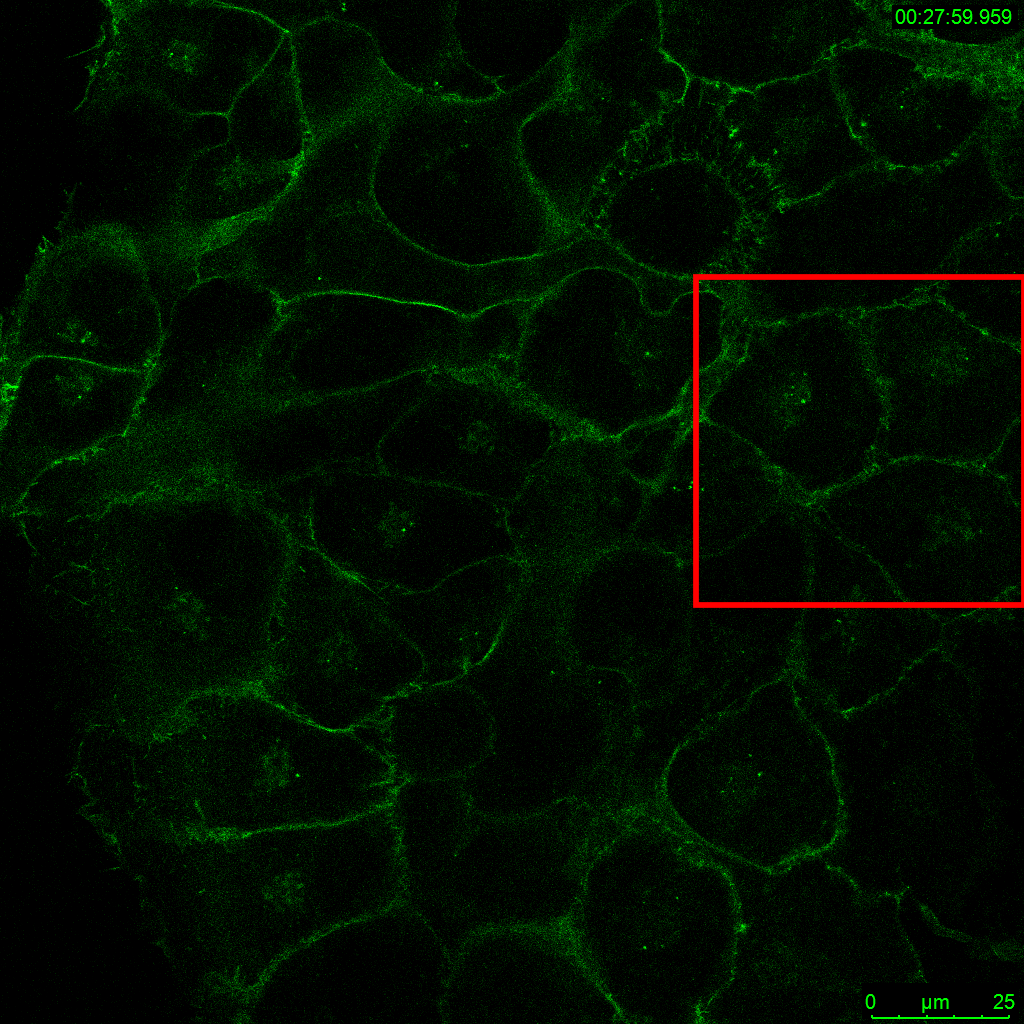

Supplement: Supplementary file 4 — Source Data Fig. 4 [file 44319_2023_45_MOESM4_ESM.zip › Fig 4/Fig 4G/F4G2 30min.tif]

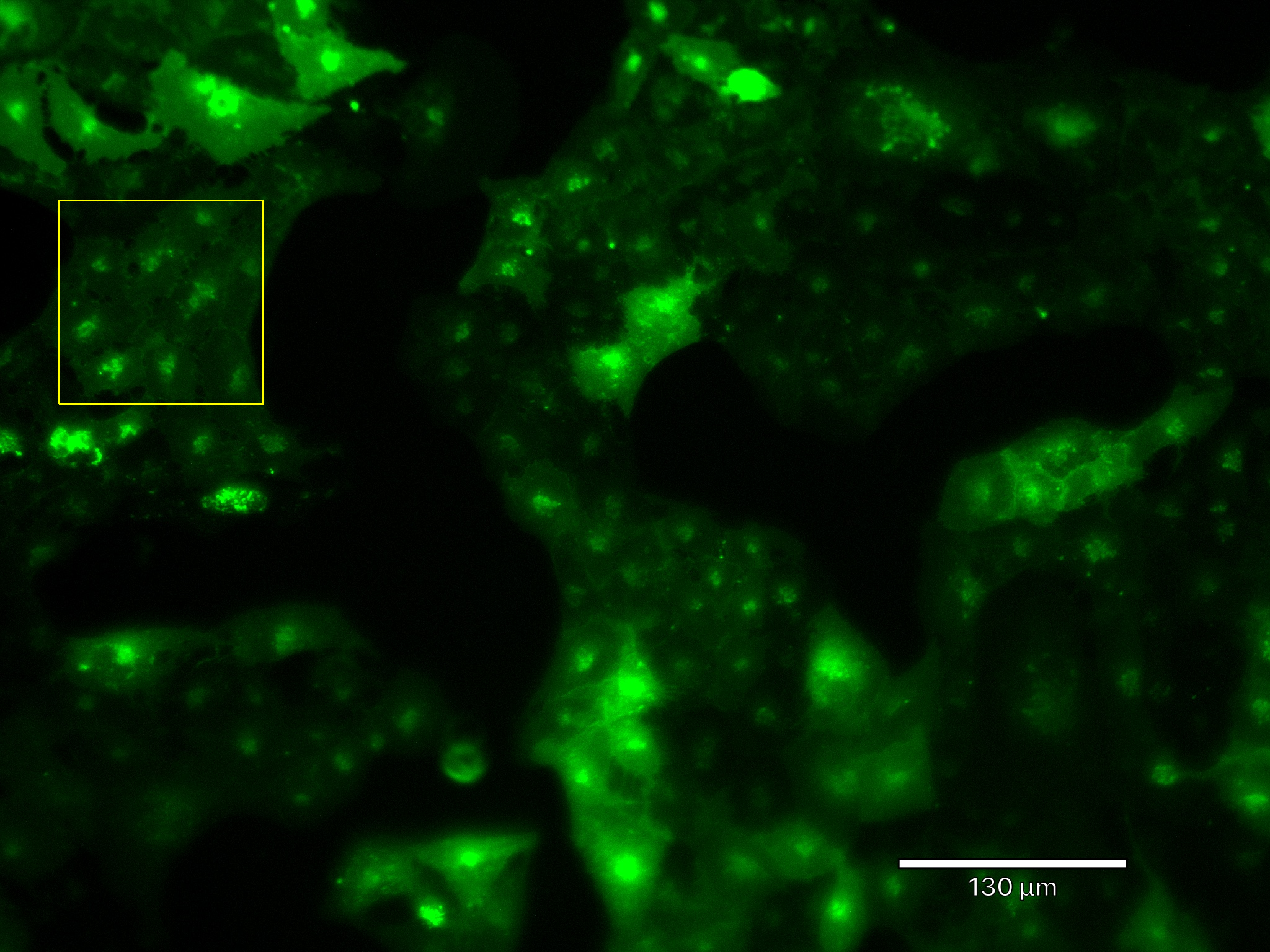

Supplement: Supplementary file 4 — Source Data Fig. 4 [file 44319_2023_45_MOESM4_ESM.zip › Fig 4/Fig 4A/F4A2_+cGAMP.png]

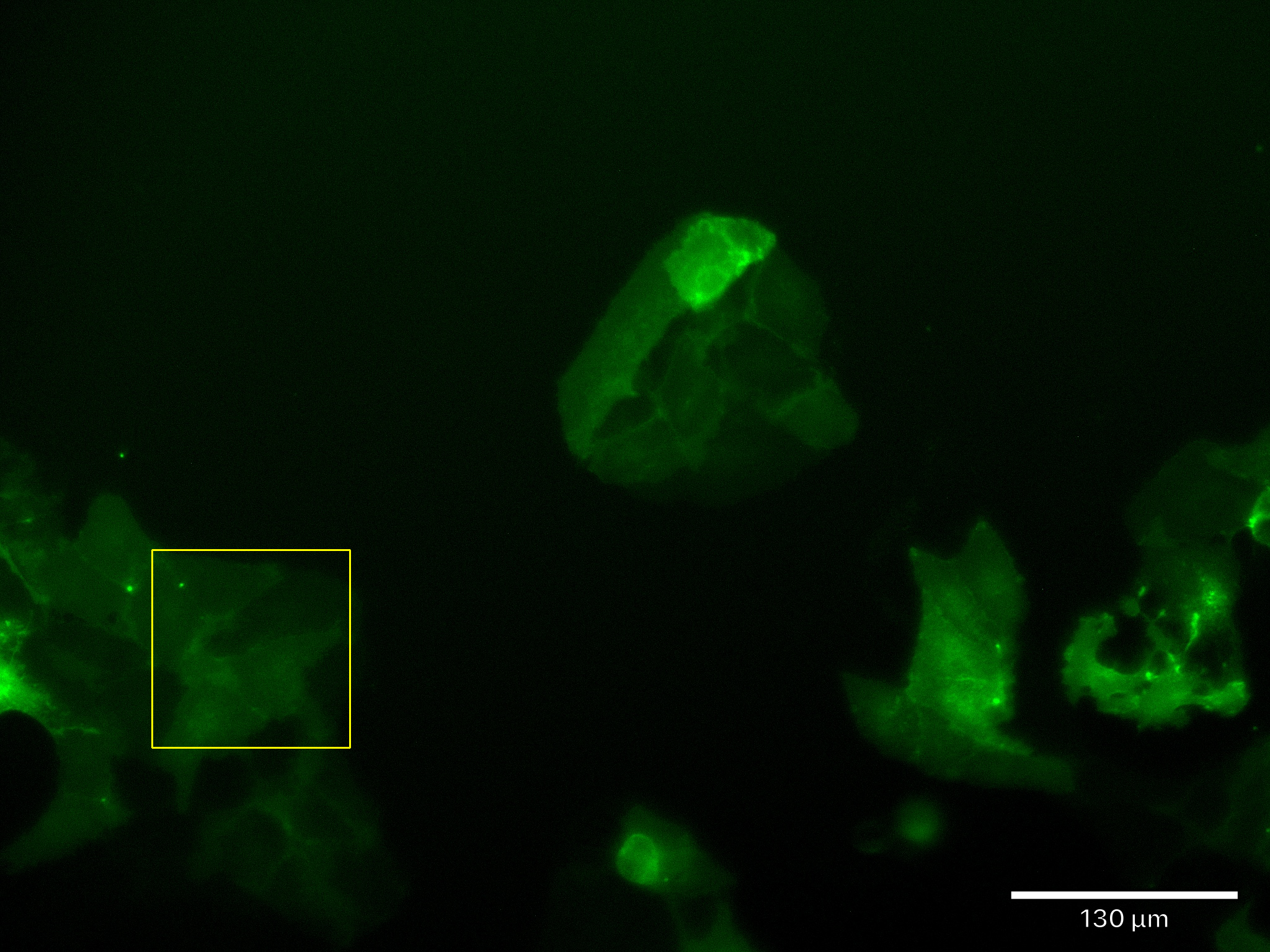

Supplement: Supplementary file 4 — Source Data Fig. 4 [file 44319_2023_45_MOESM4_ESM.zip › Fig 4/Fig 4A/F4A3_-diABZI.png]

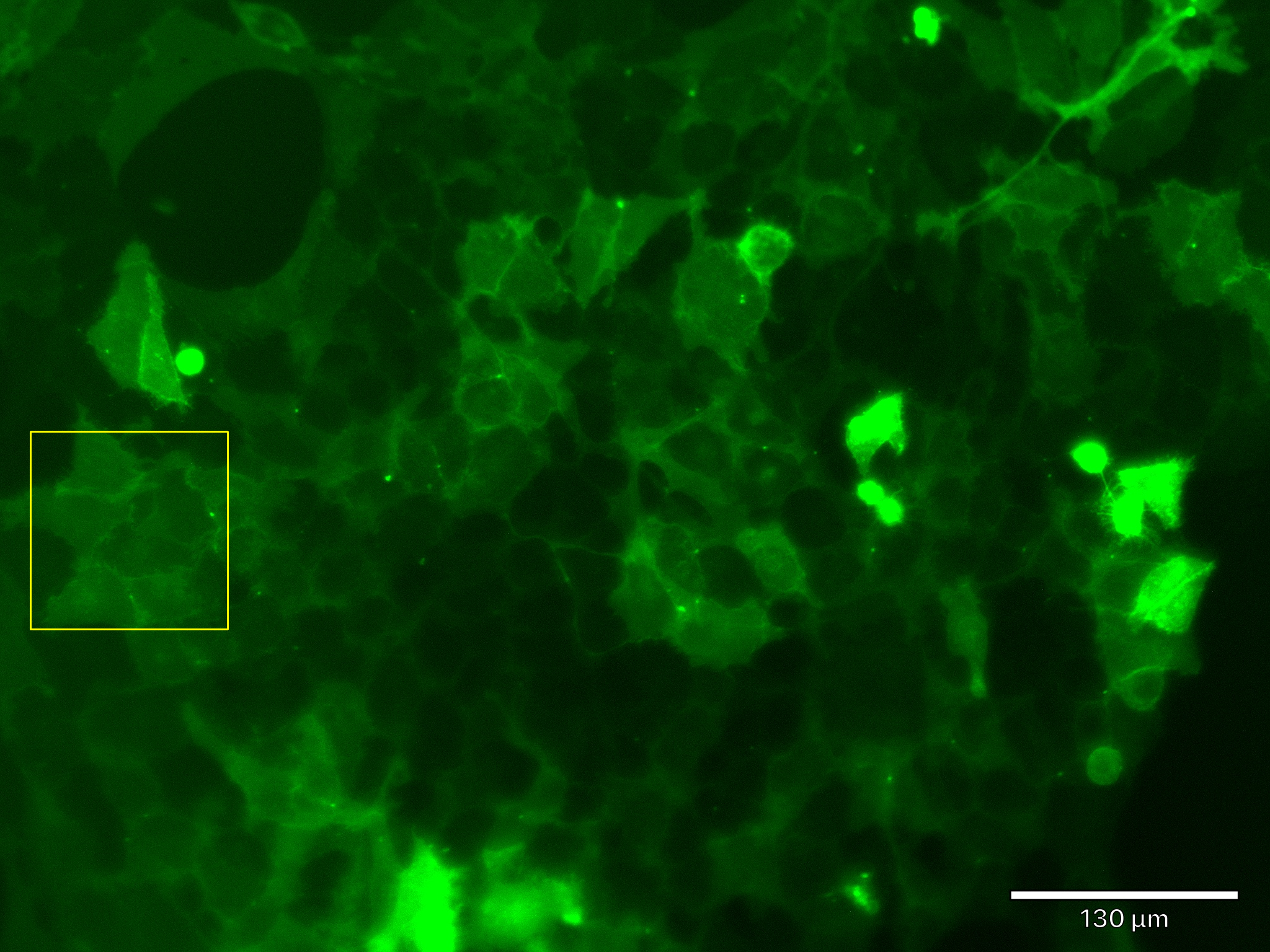

Supplement: Supplementary file 4 — Source Data Fig. 4 [file 44319_2023_45_MOESM4_ESM.zip › Fig 4/Fig 4A/F4A1_- cGAMP.png]

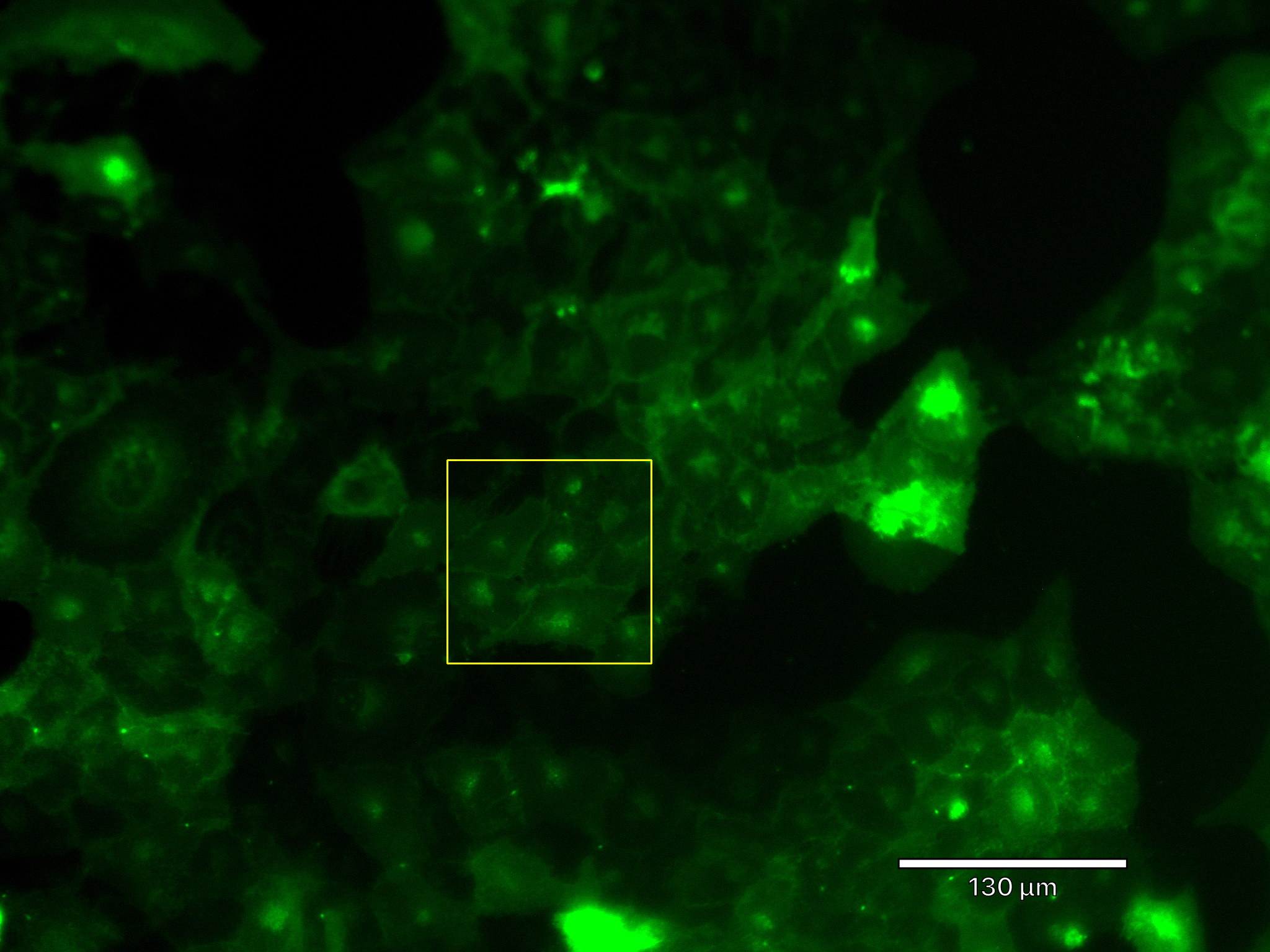

Supplement: Supplementary file 4 — Source Data Fig. 4 [file 44319_2023_45_MOESM4_ESM.zip › Fig 4/Fig 4A/F4A4_+diABZI.png]

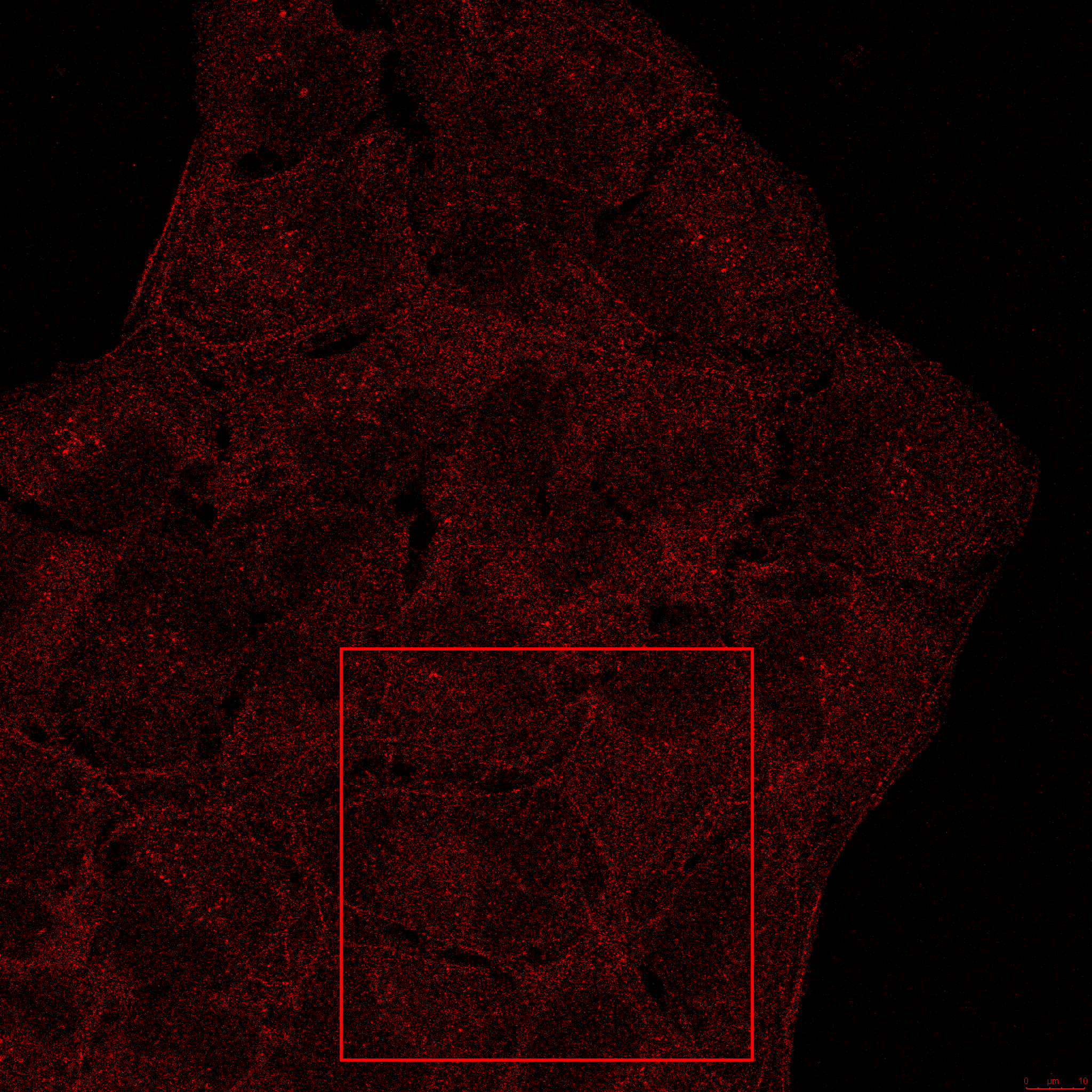

Supplement: Supplementary file 6 — Source Data Fig. 6 [file 44319_2023_45_MOESM6_ESM.zip › Fig 6/Fig 6I/Fig6I-3 U2OS FST LC3-R594_cGAMP(+C53-30'after) 90'.tif]

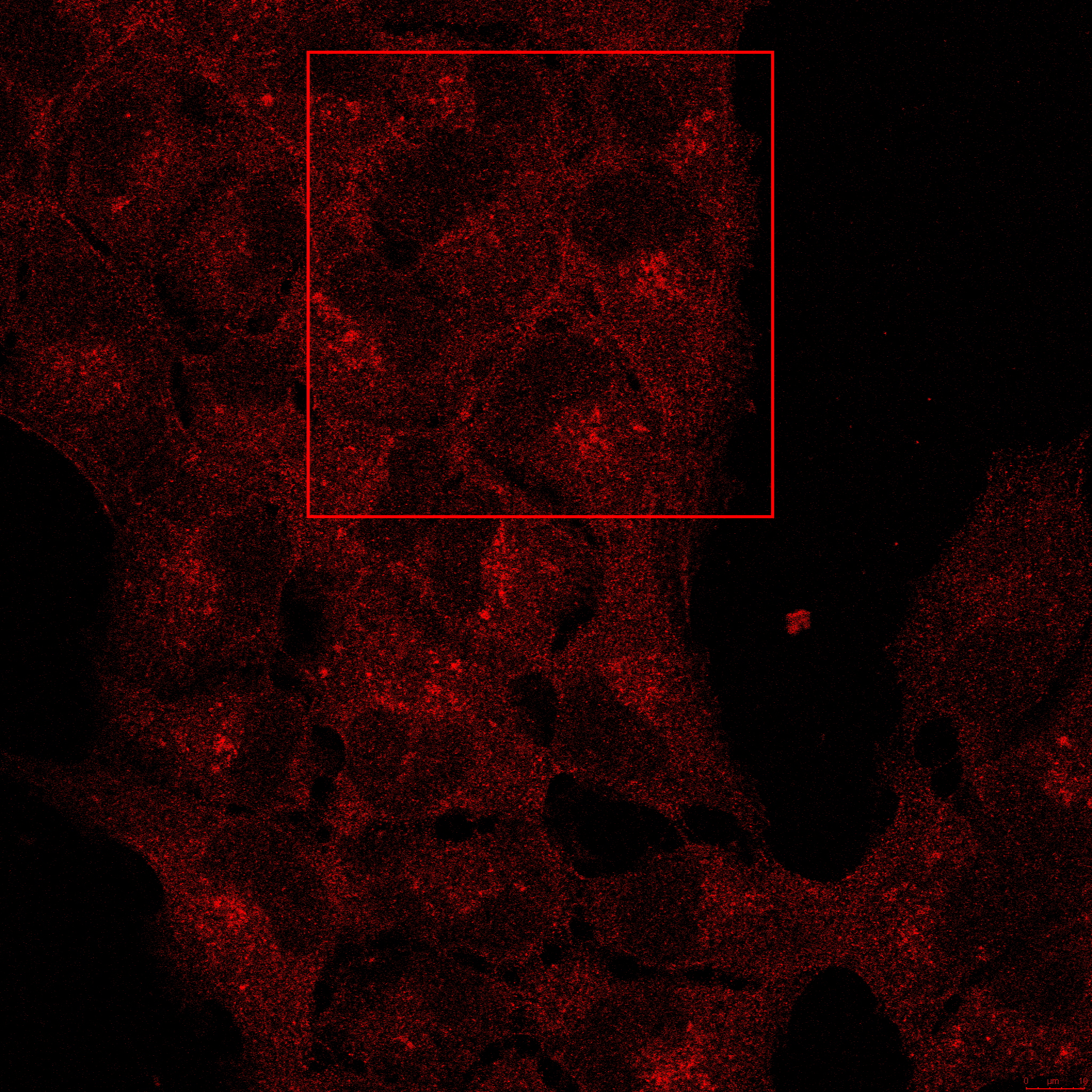

Supplement: Supplementary file 6 — Source Data Fig. 6 [file 44319_2023_45_MOESM6_ESM.zip › Fig 6/Fig 6I/Fig6I-2 U2OS FST LC3-R594_cGAMP 90'.tif]

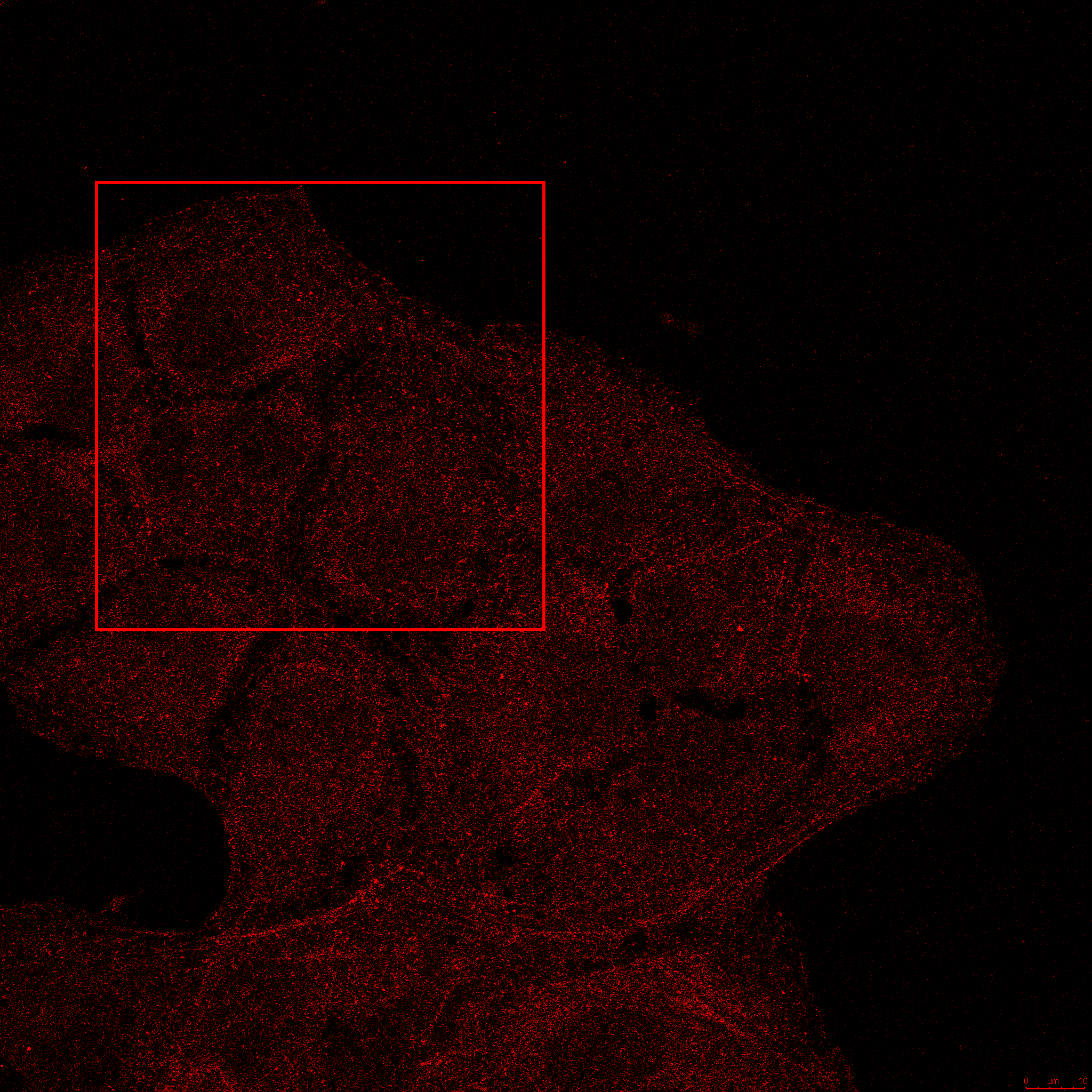

Supplement: Supplementary file 6 — Source Data Fig. 6 [file 44319_2023_45_MOESM6_ESM.zip › Fig 6/Fig 6I/Fig6I-1 U2OS FST LC3-R594_cGAMP 0'.tif]

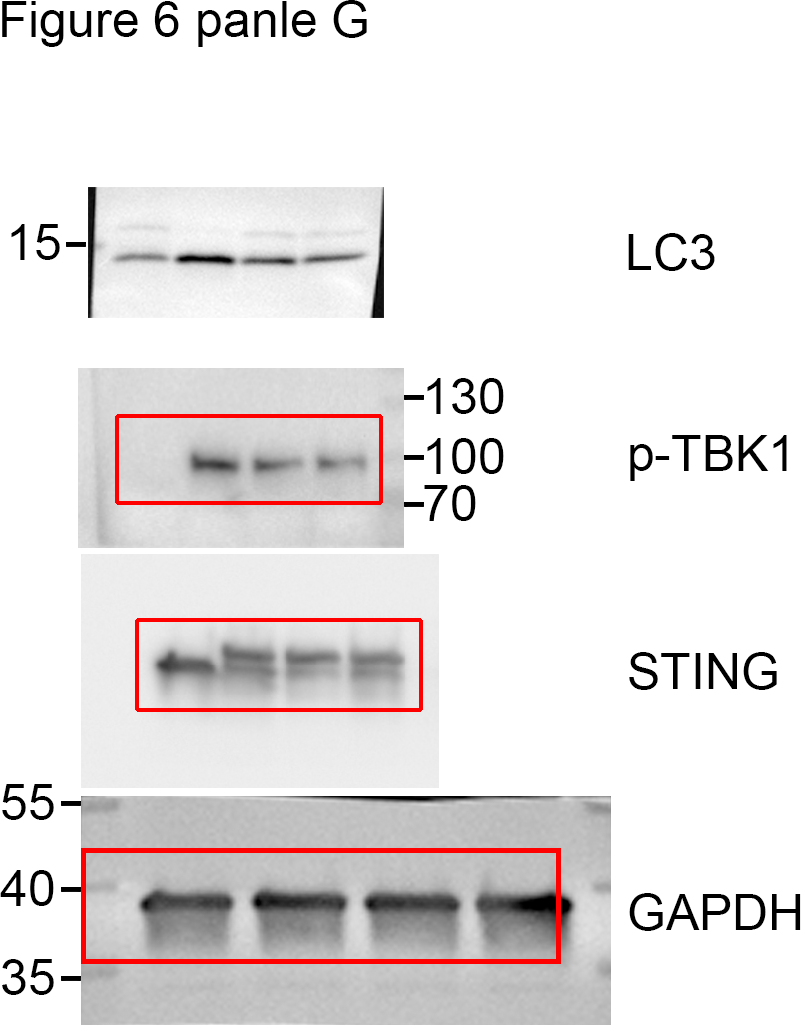

Supplement: Supplementary file 6 — Source Data Fig. 6 [file 44319_2023_45_MOESM6_ESM.zip › Fig 6/Fig 6G/western.TIF]

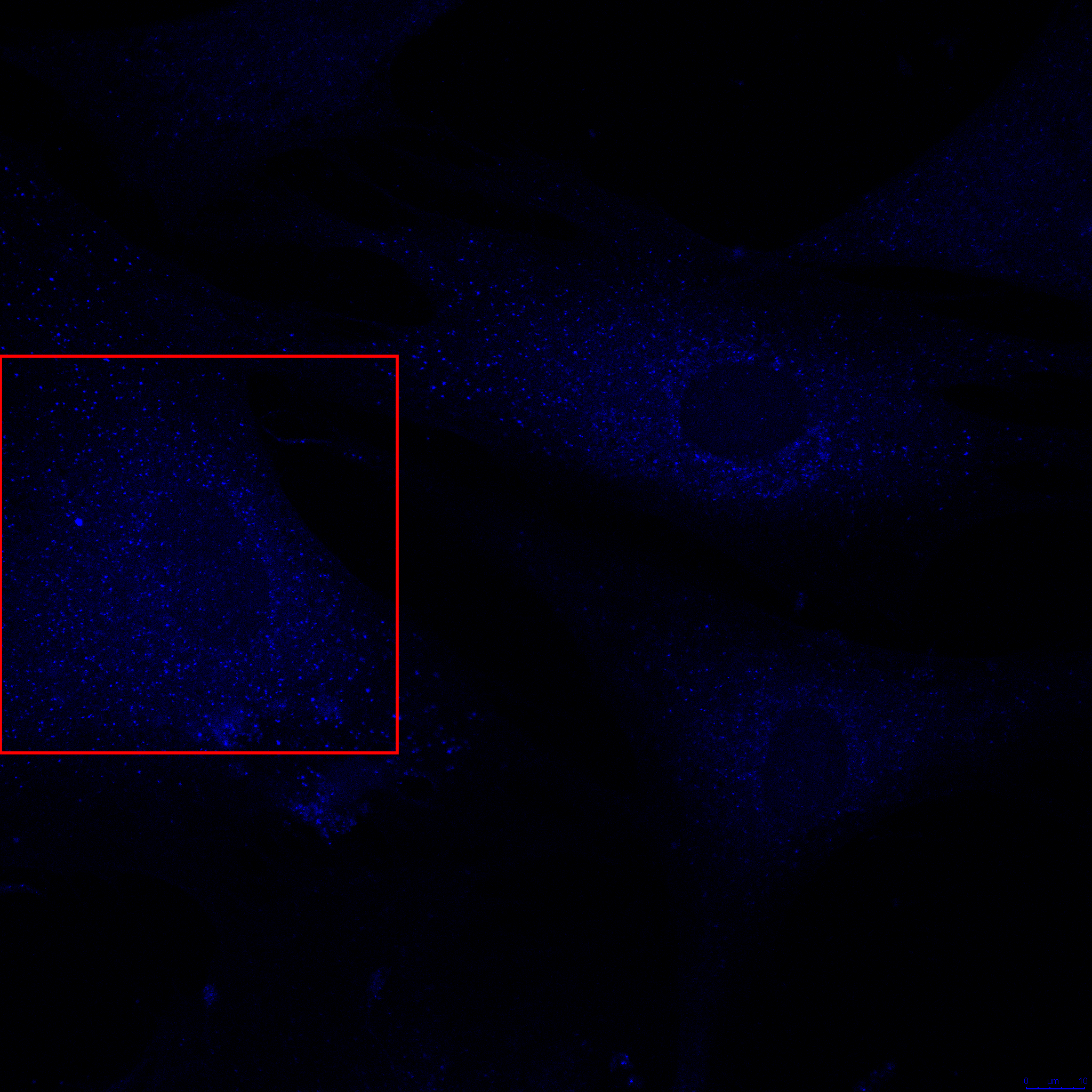

Supplement: Supplementary file 6 — Source Data Fig. 6 [file 44319_2023_45_MOESM6_ESM.zip › Fig 6/Fig 6A/F6A7 BJ ST-blue_cGAMP+C53 60'.tif]

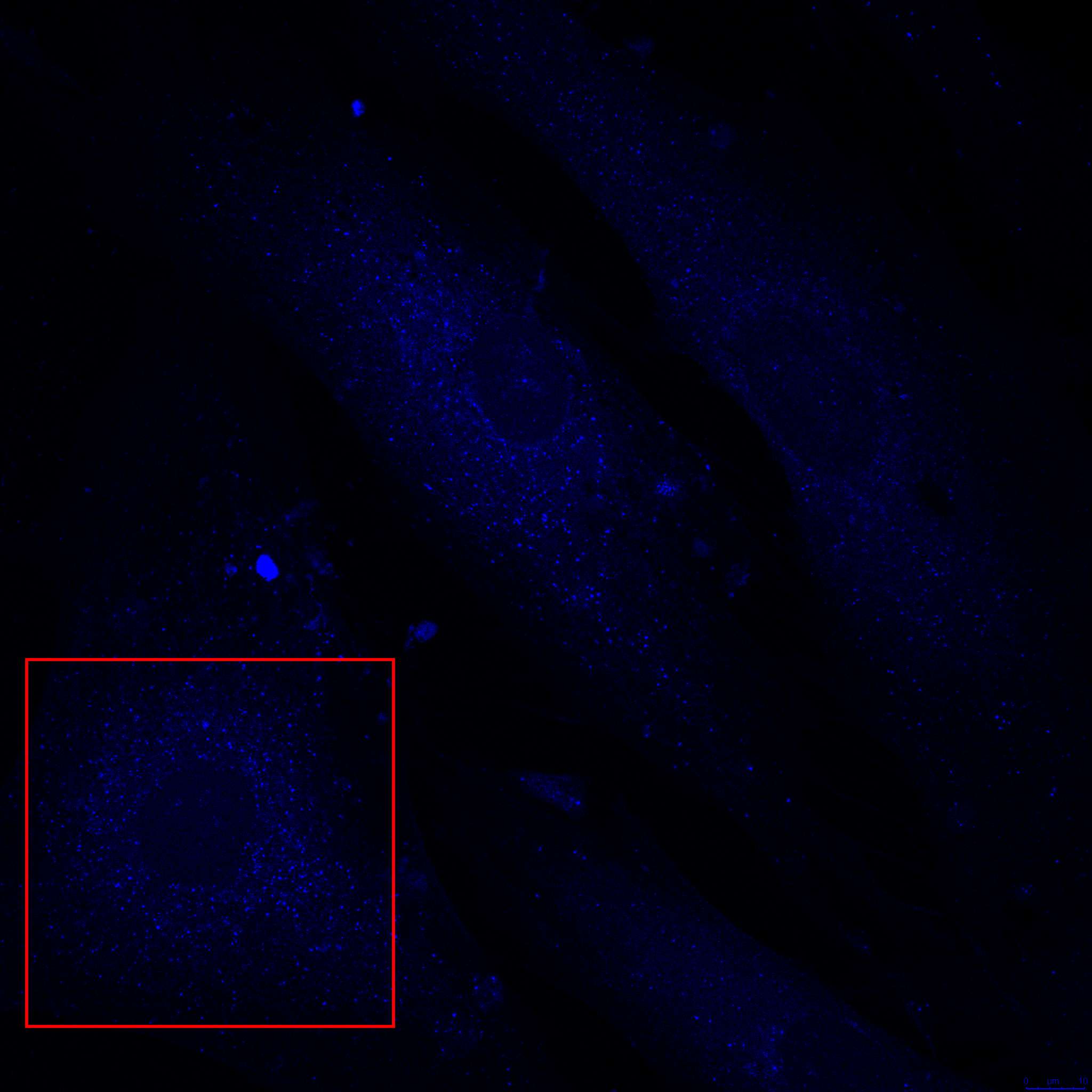

Supplement: Supplementary file 6 — Source Data Fig. 6 [file 44319_2023_45_MOESM6_ESM.zip › Fig 6/Fig 6A/F6A8 BJ ST-blue_cGAMP+C53 90'.tif]

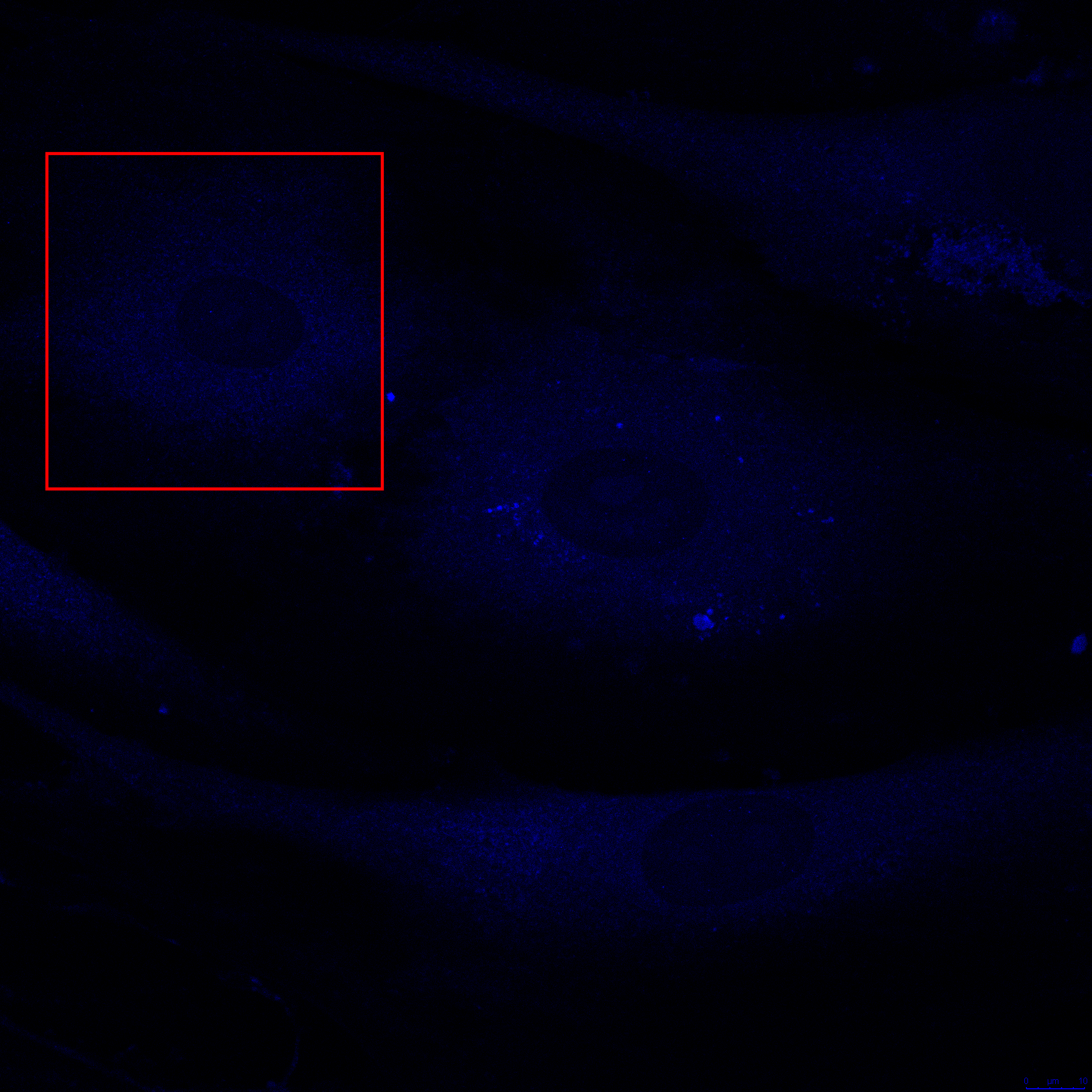

Supplement: Supplementary file 6 — Source Data Fig. 6 [file 44319_2023_45_MOESM6_ESM.zip › Fig 6/Fig 6A/F6A5 BJ ST-blue_cGAMP+C53 0'.tif]

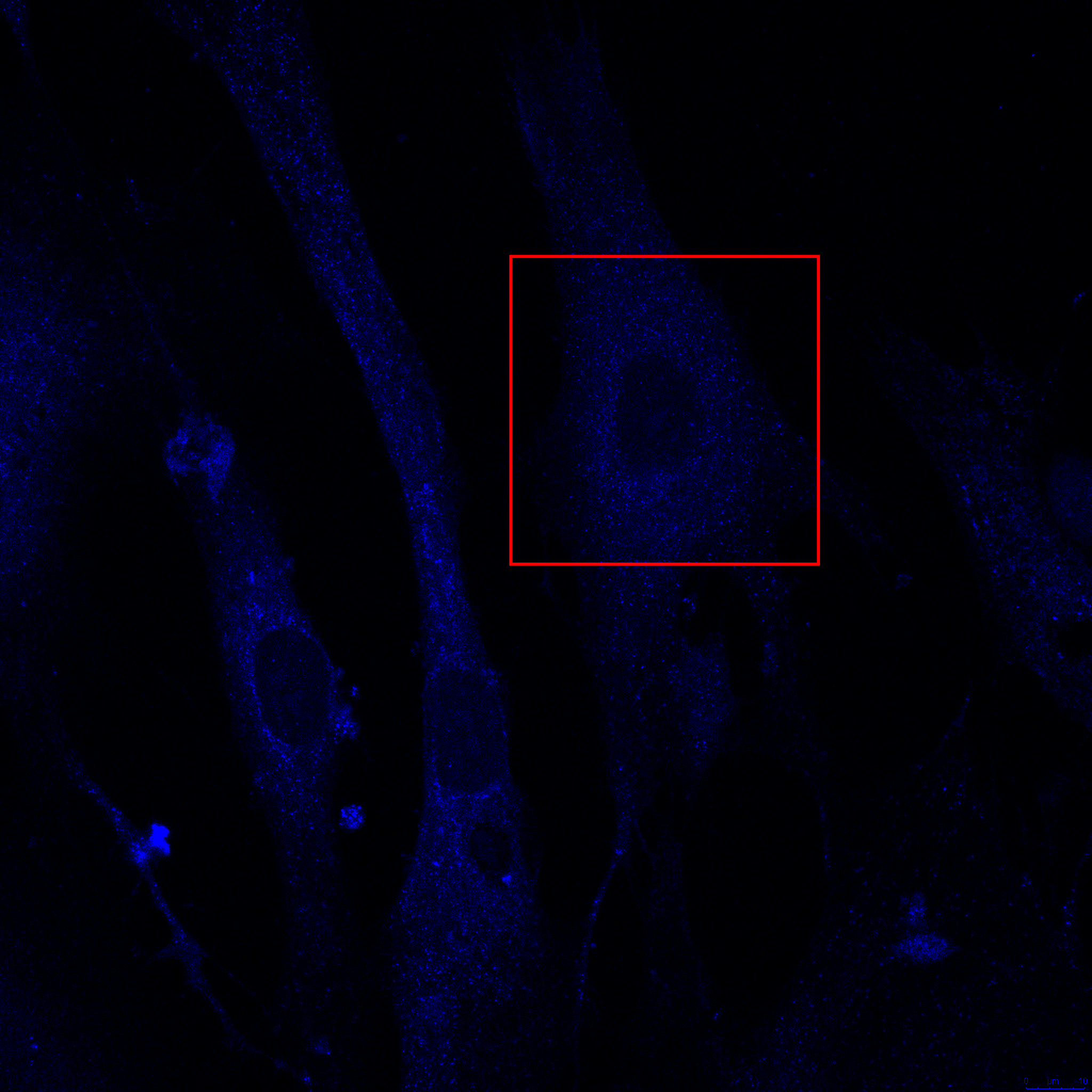

Supplement: Supplementary file 6 — Source Data Fig. 6 [file 44319_2023_45_MOESM6_ESM.zip › Fig 6/Fig 6A/F6A6 BJ ST-blue_cGAMP+C53 30'.tif]

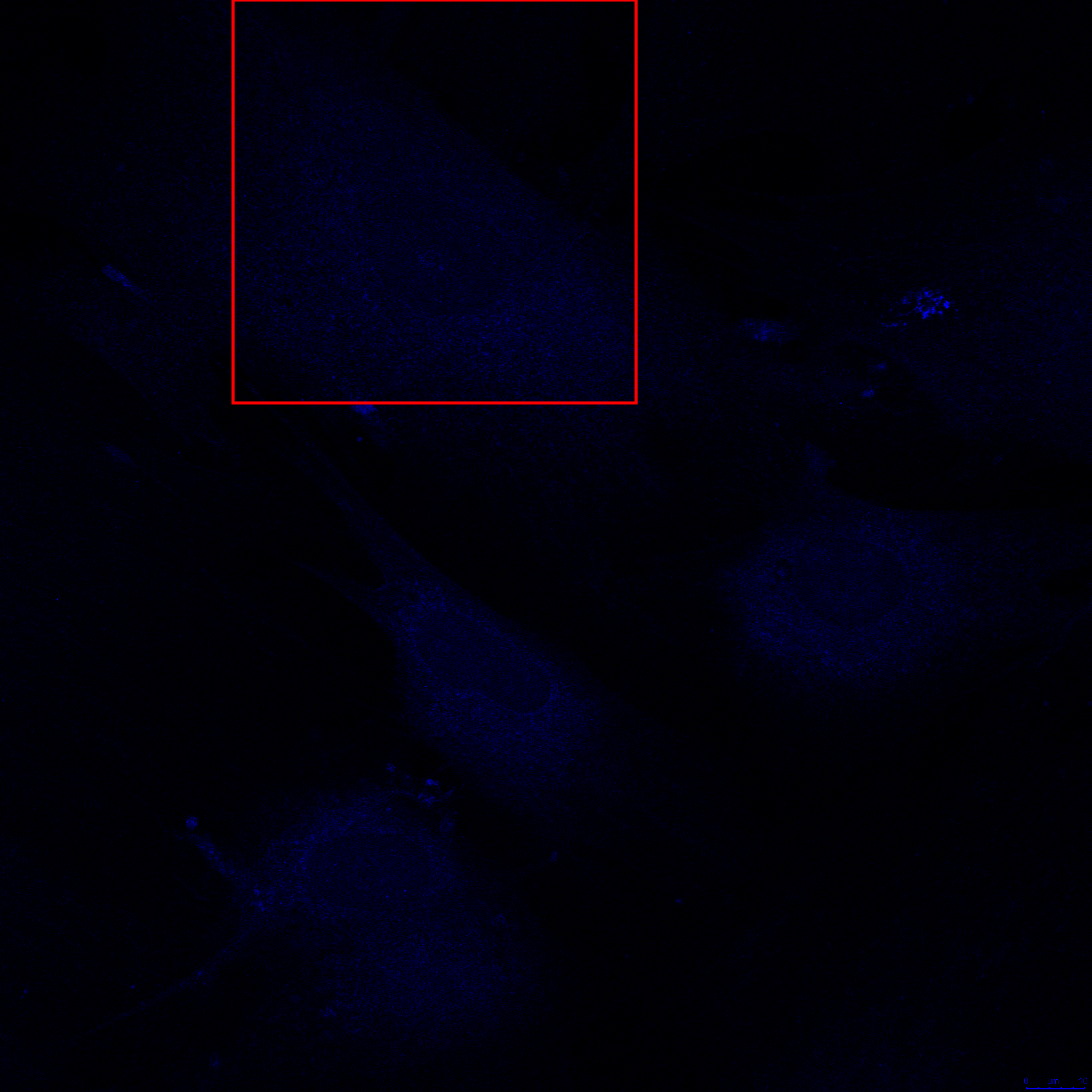

Supplement: Supplementary file 6 — Source Data Fig. 6 [file 44319_2023_45_MOESM6_ESM.zip › Fig 6/Fig 6A/F6A1 BJ ST-blue_cGAMP 0'.tif]

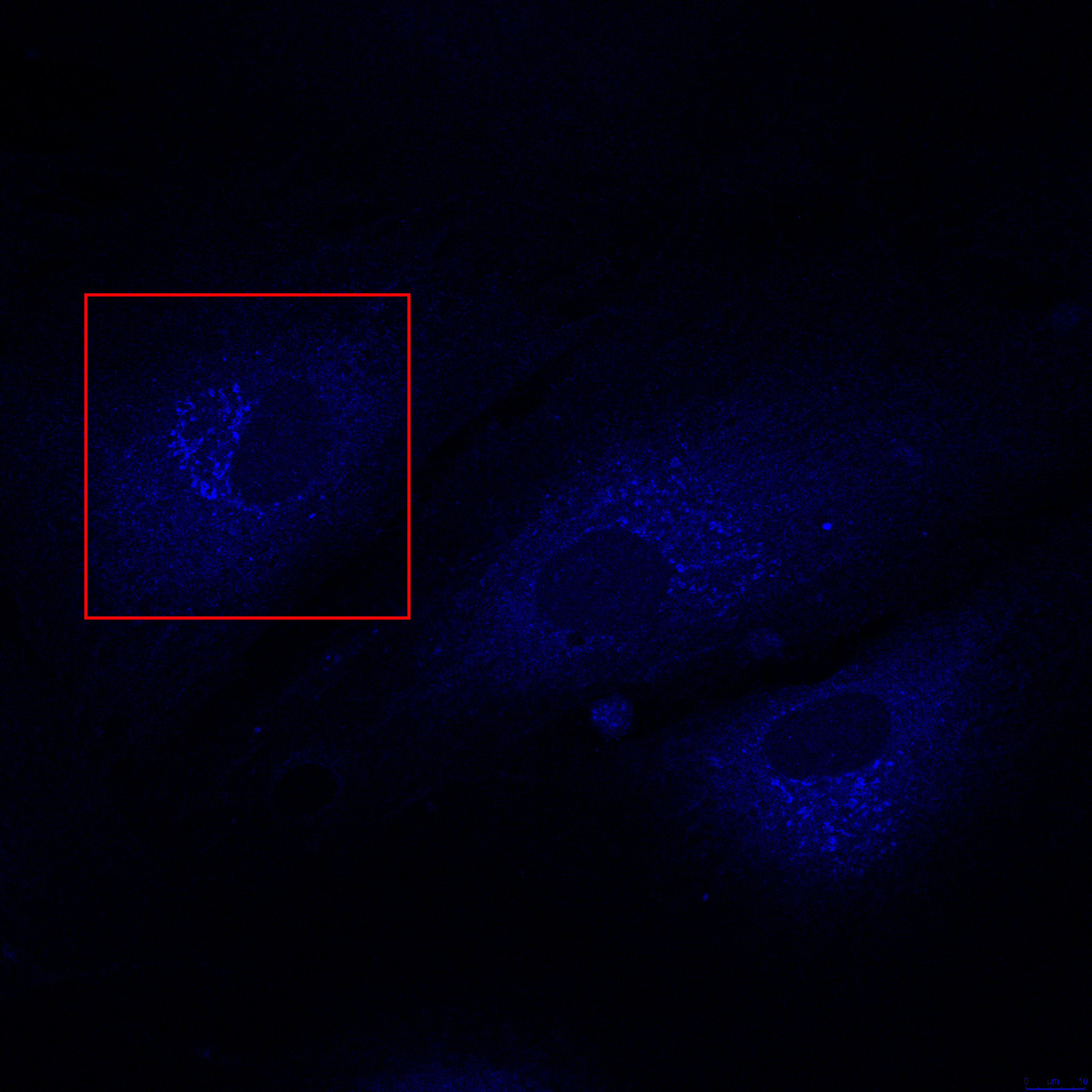

Supplement: Supplementary file 6 — Source Data Fig. 6 [file 44319_2023_45_MOESM6_ESM.zip › Fig 6/Fig 6A/F6A2 BJ ST-blue_cGAMP 30'.tif]

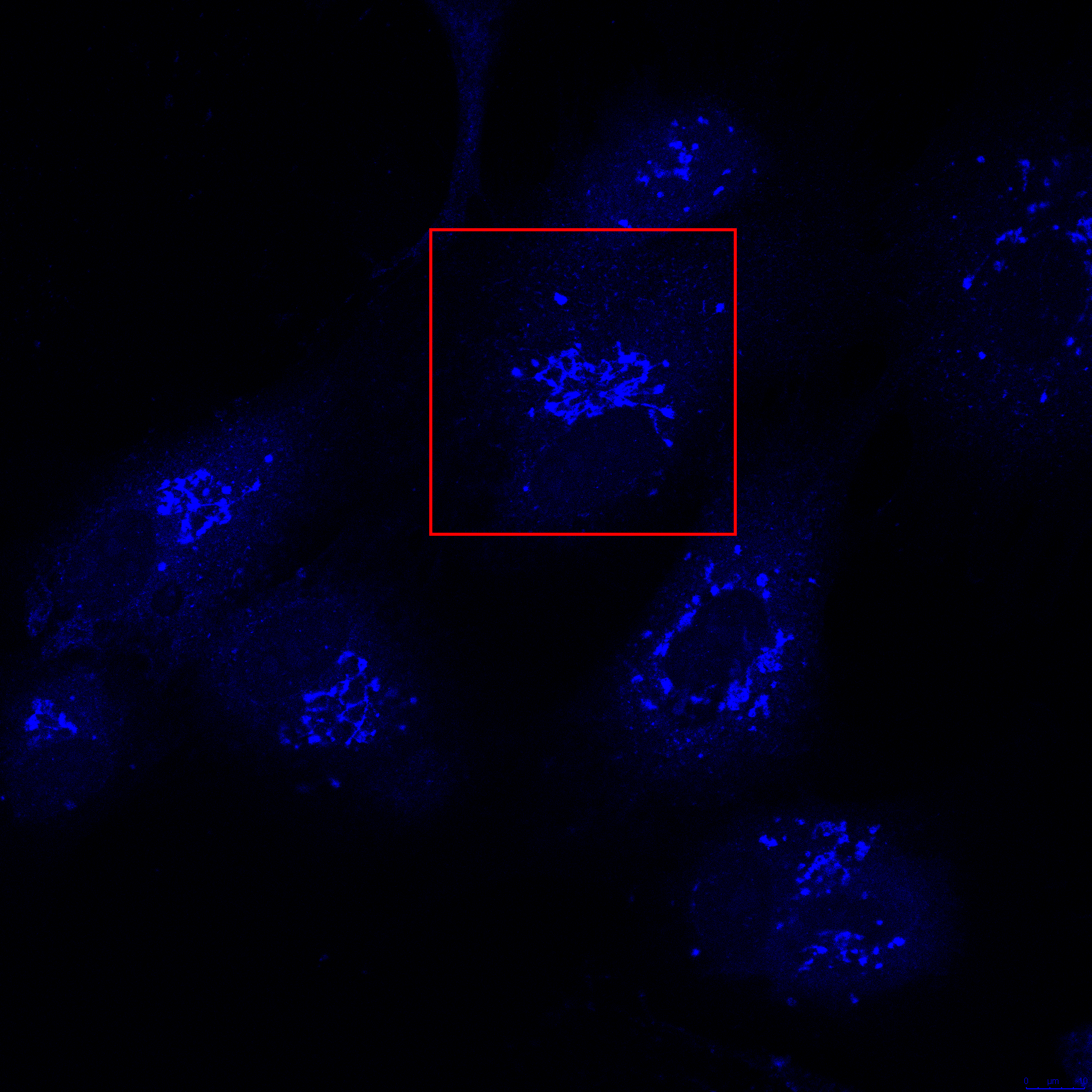

Supplement: Supplementary file 6 — Source Data Fig. 6 [file 44319_2023_45_MOESM6_ESM.zip › Fig 6/Fig 6A/F6A3 BJ ST-blue_cGAMP 60'.tif]

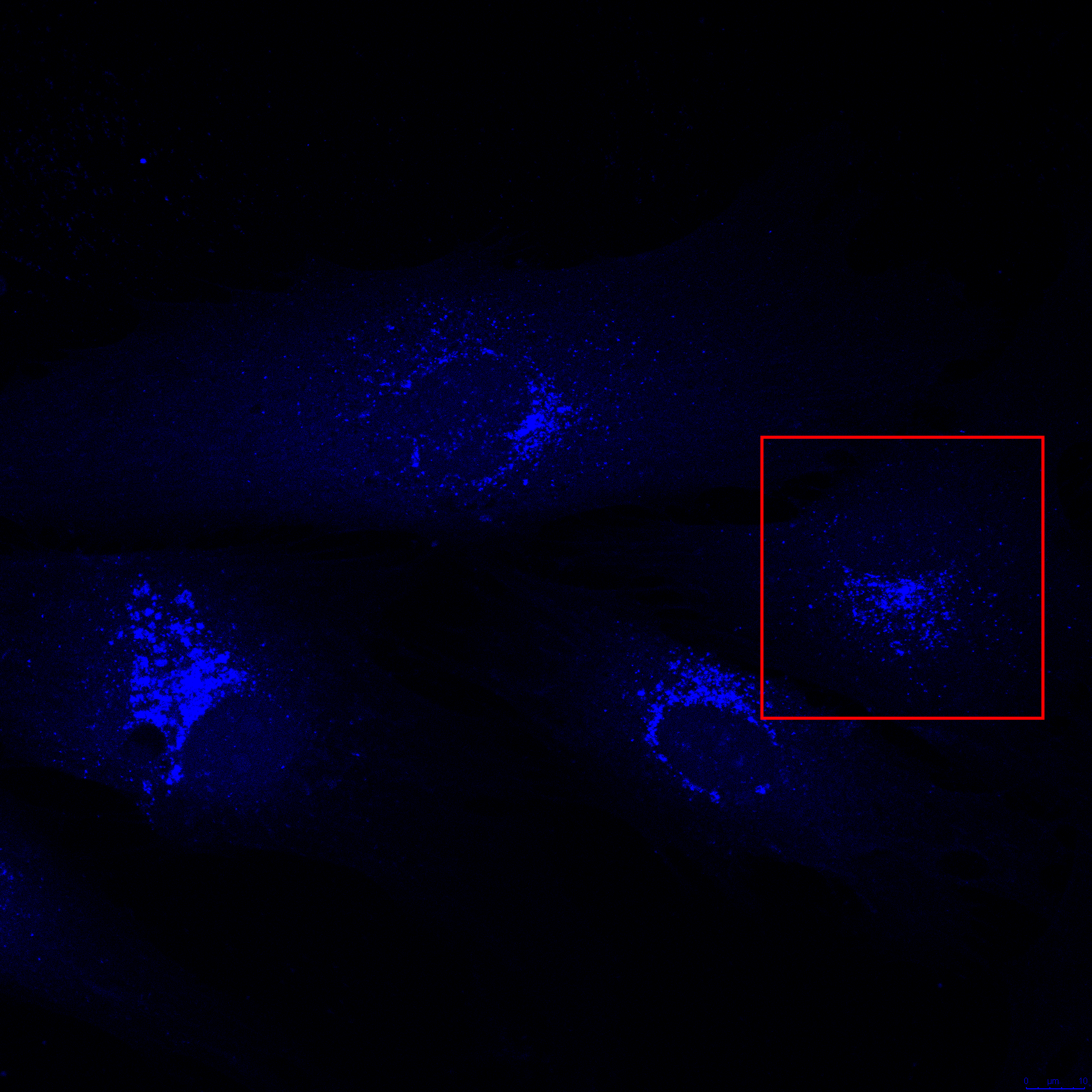

Supplement: Supplementary file 6 — Source Data Fig. 6 [file 44319_2023_45_MOESM6_ESM.zip › Fig 6/Fig 6A/F6A4 BJ ST-blue_cGAMP 90'.tif]

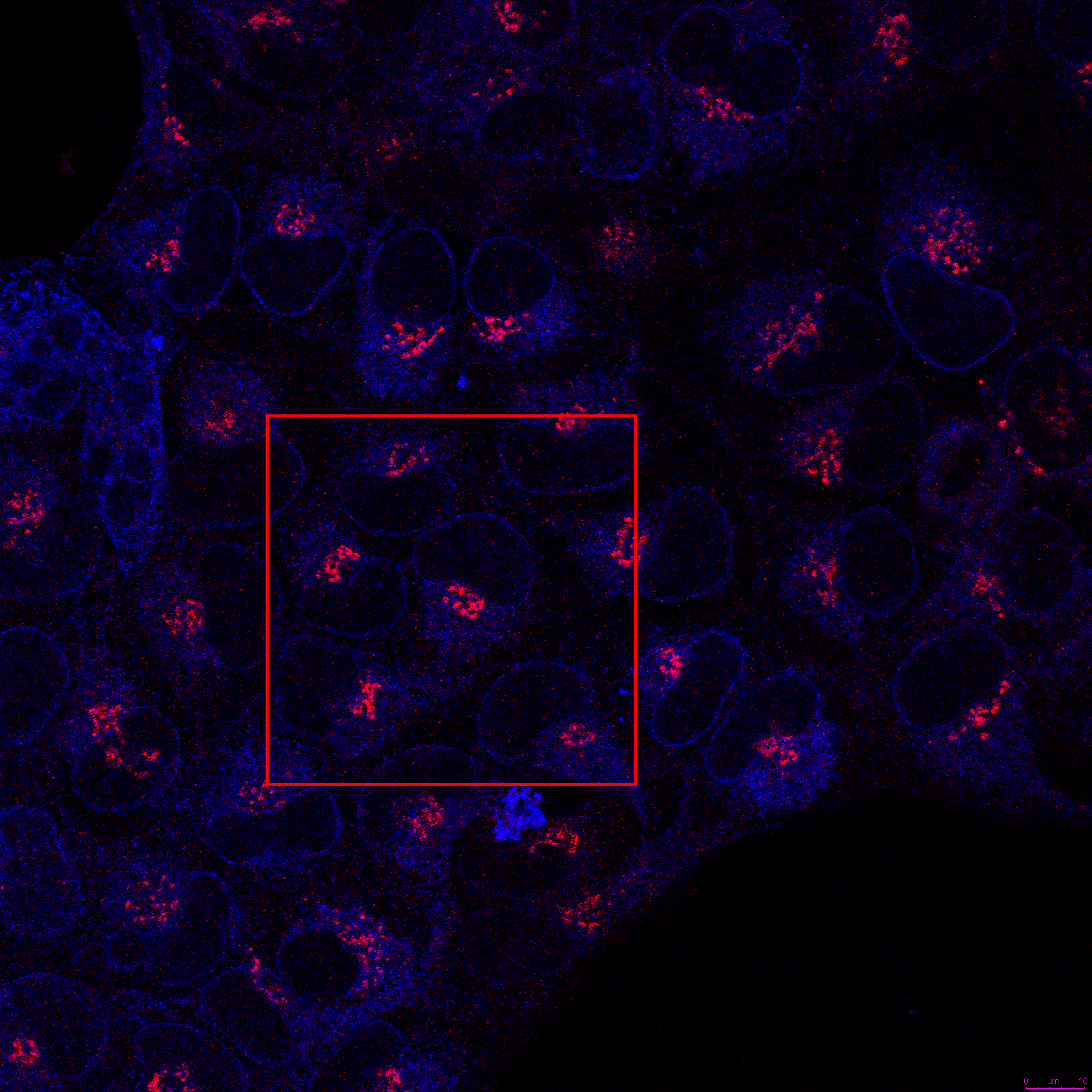

Supplement: Supplementary file 6 — Source Data Fig. 6 [file 44319_2023_45_MOESM6_ESM.zip › Fig 6/Fig 6D/Fig6D-1 U2OS FST#2 st-blue golgin97-594 _cGAMP-0'.tif]

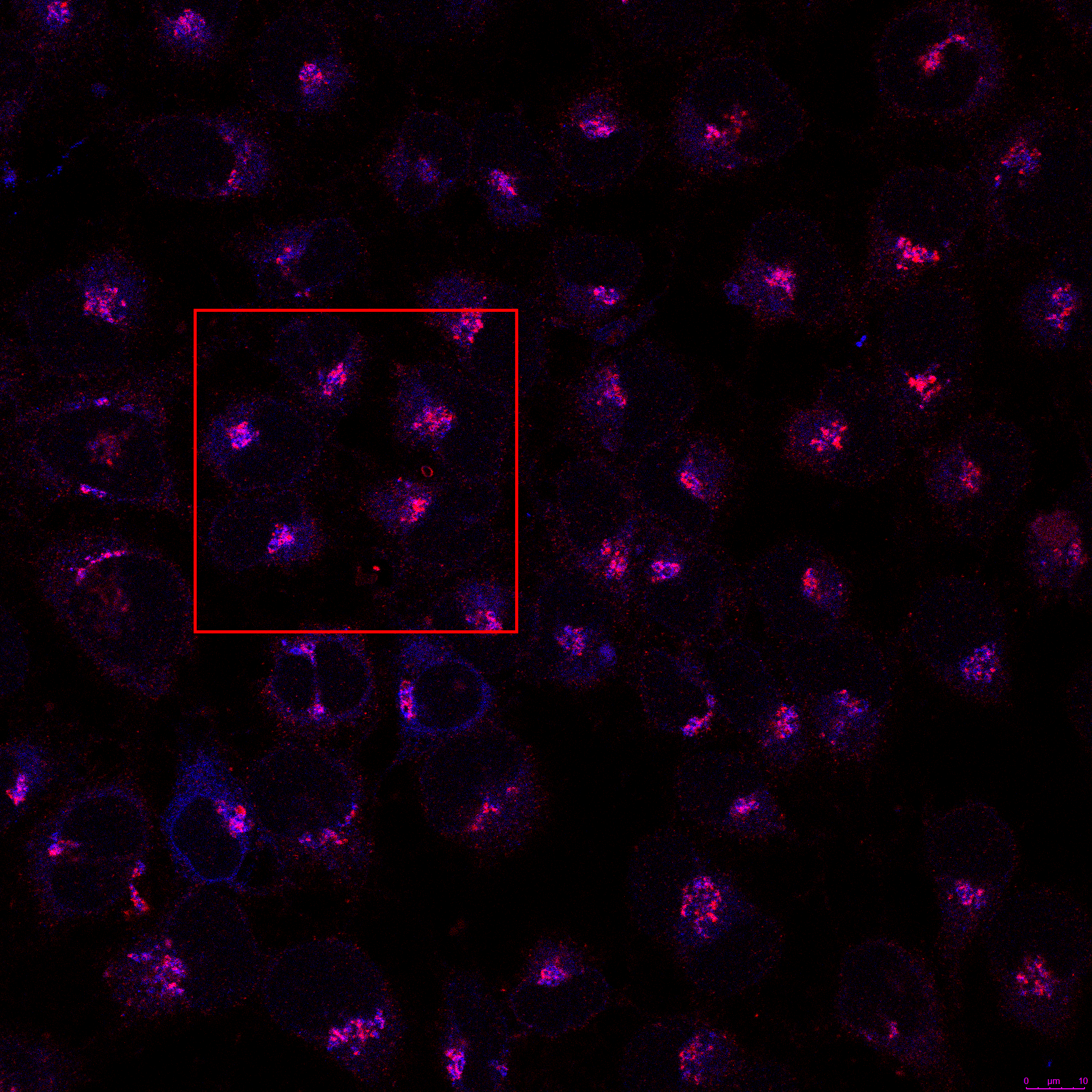

Supplement: Supplementary file 6 — Source Data Fig. 6 [file 44319_2023_45_MOESM6_ESM.zip › Fig 6/Fig 6D/Fig6D-2 U2OS FST#2 st-blue golgin97-594 _cGAMP 30'.tif]

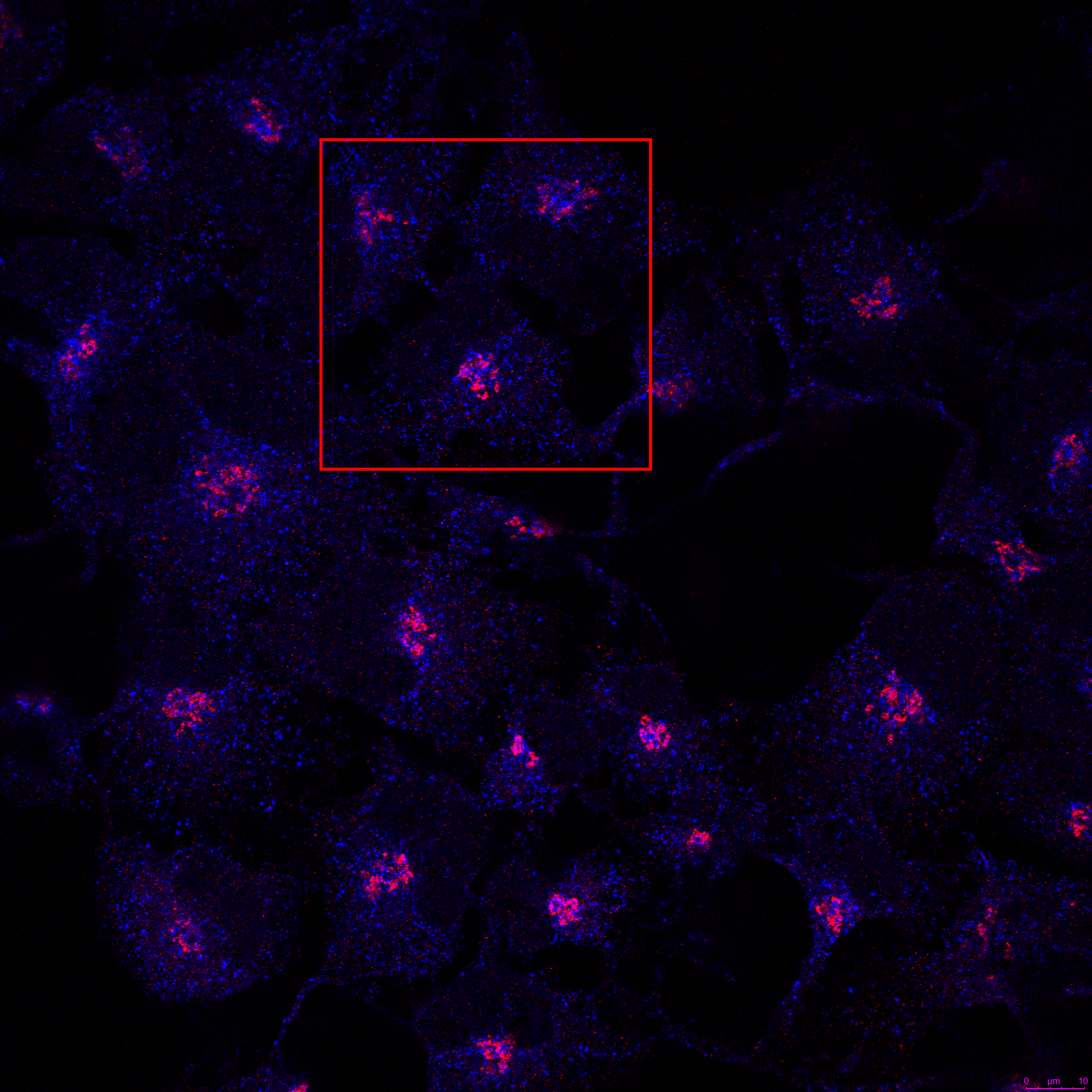

Supplement: Supplementary file 6 — Source Data Fig. 6 [file 44319_2023_45_MOESM6_ESM.zip › Fig 6/Fig 6D/Fig6D-4 U2OS FST#2 st-blue golgin97-594 _cGAMP 30'+C53 90'.tif]

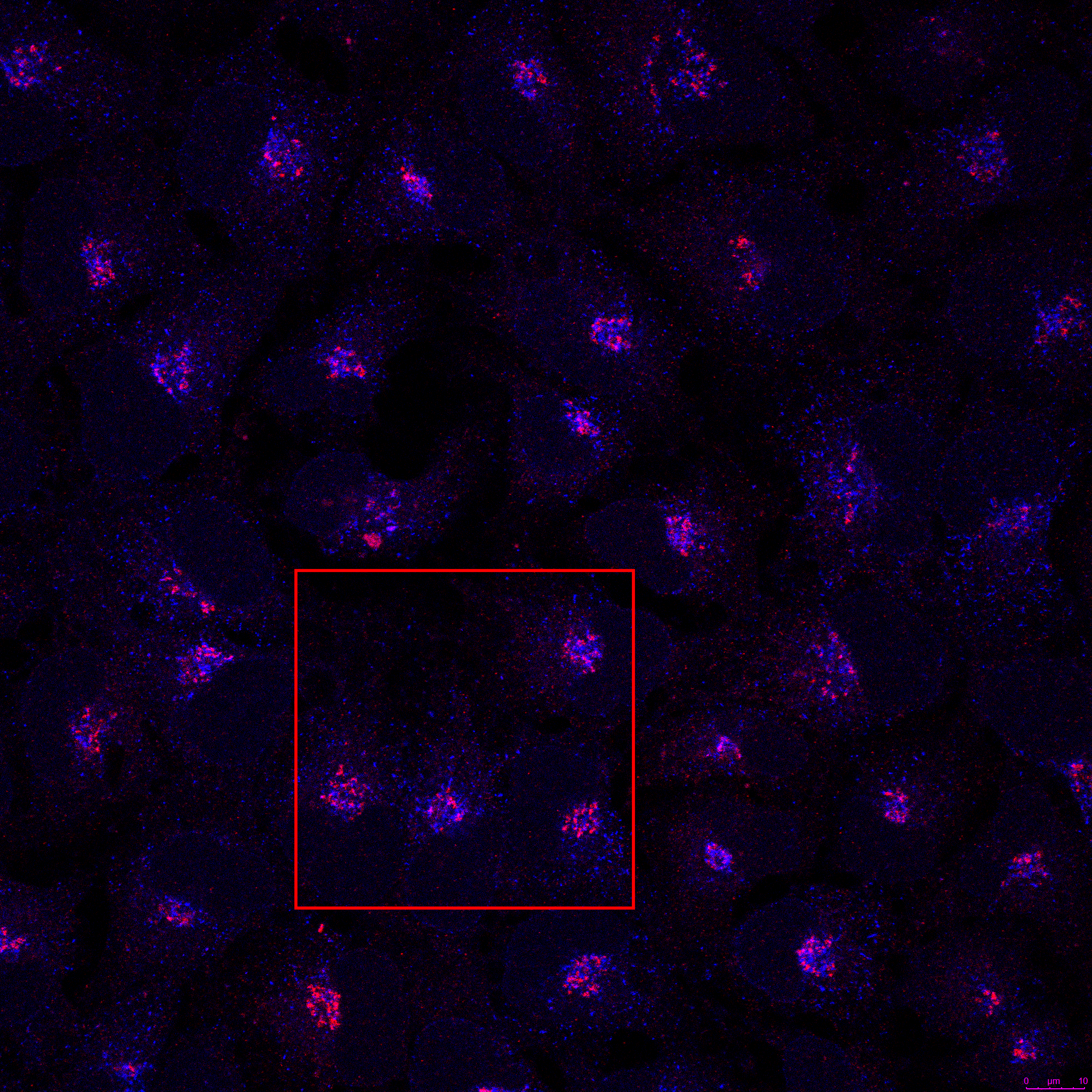

Supplement: Supplementary file 6 — Source Data Fig. 6 [file 44319_2023_45_MOESM6_ESM.zip › Fig 6/Fig 6D/Fig6D-3 U2OS FST#2 st-blue golgin97-594 _cGAMP 30'+C53 60'.tif]

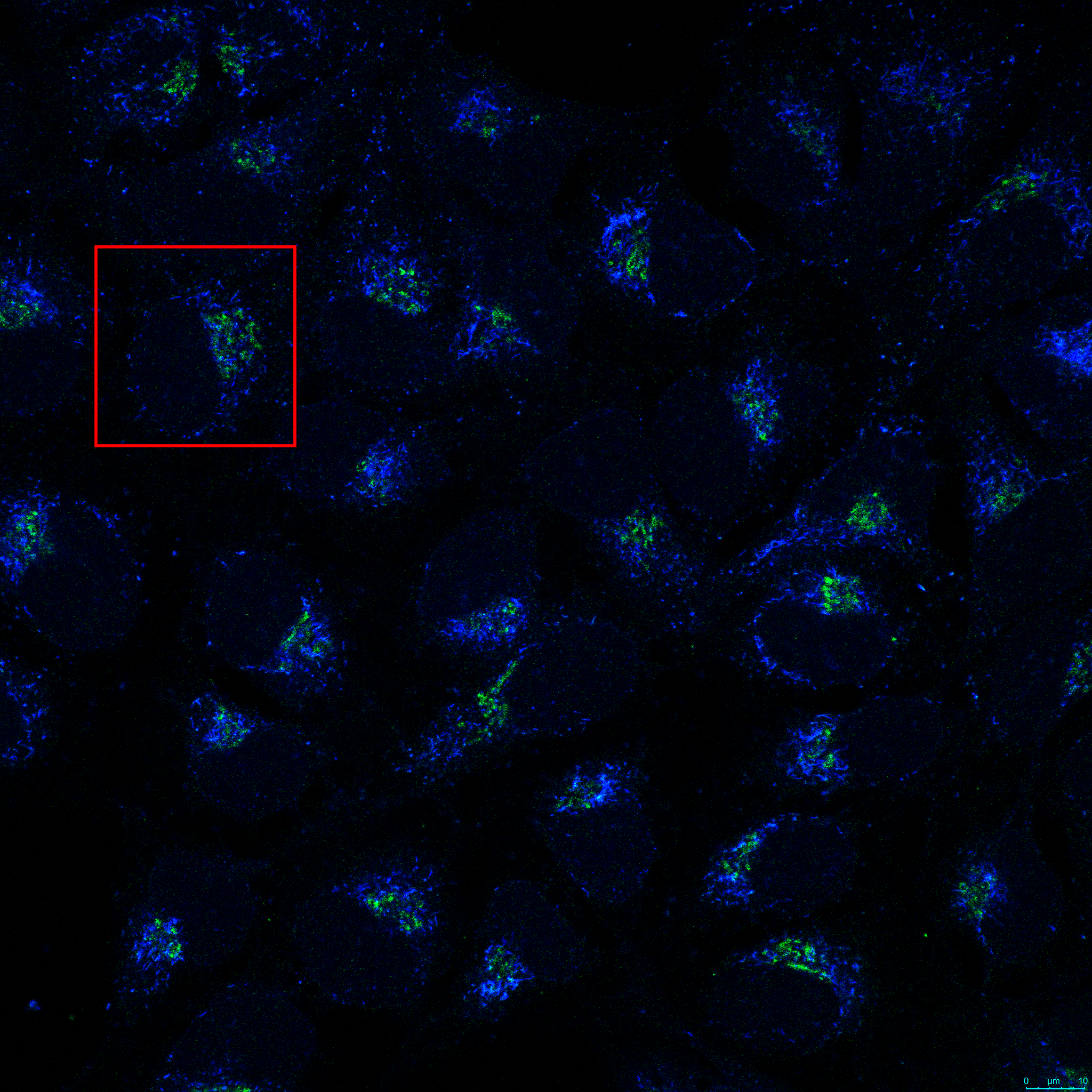

Supplement: Supplementary file 6 — Source Data Fig. 6 [file 44319_2023_45_MOESM6_ESM.zip › Fig 6/Fig 6B/F6B4.tif]

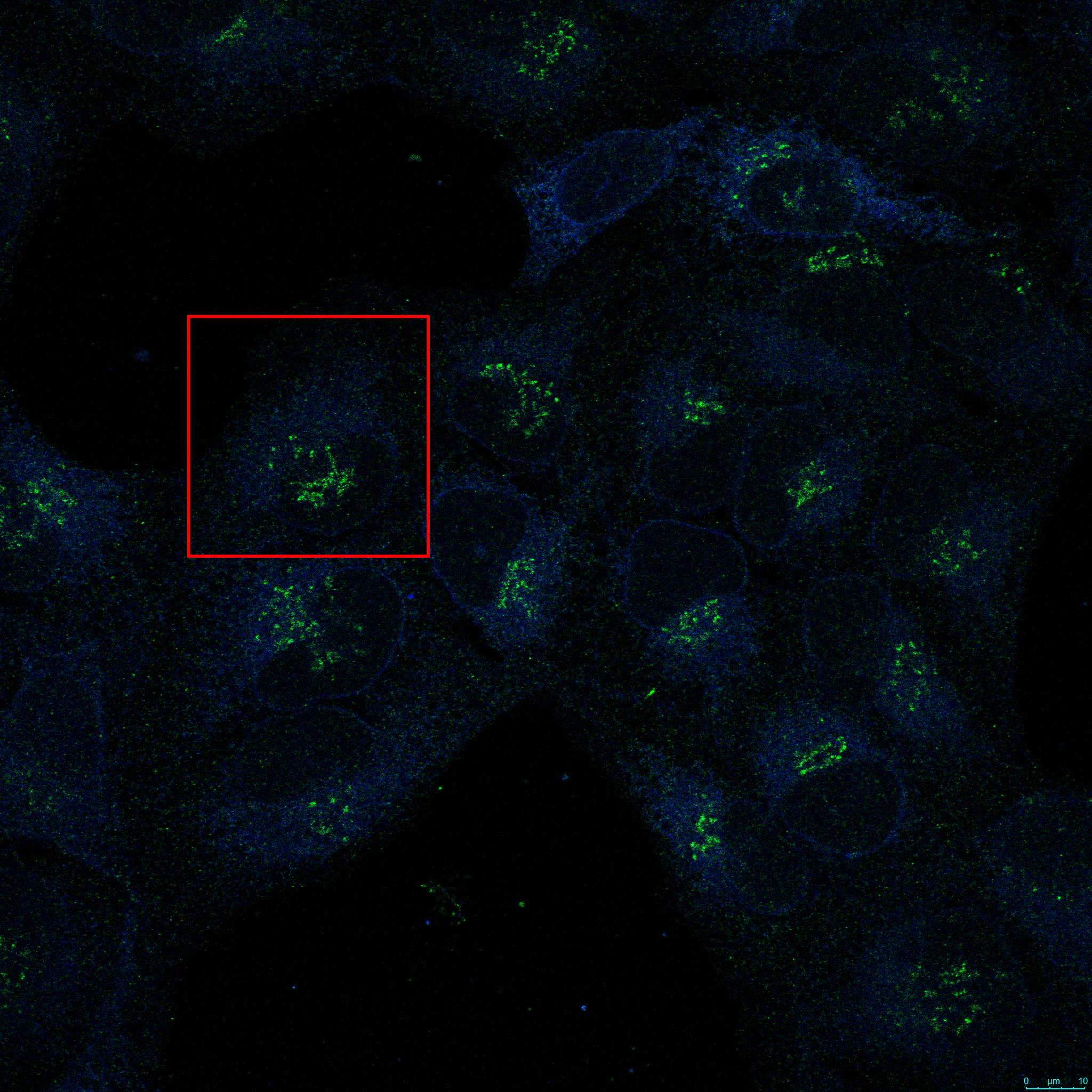

Supplement: Supplementary file 6 — Source Data Fig. 6 [file 44319_2023_45_MOESM6_ESM.zip › Fig 6/Fig 6B/F6B1.tif]

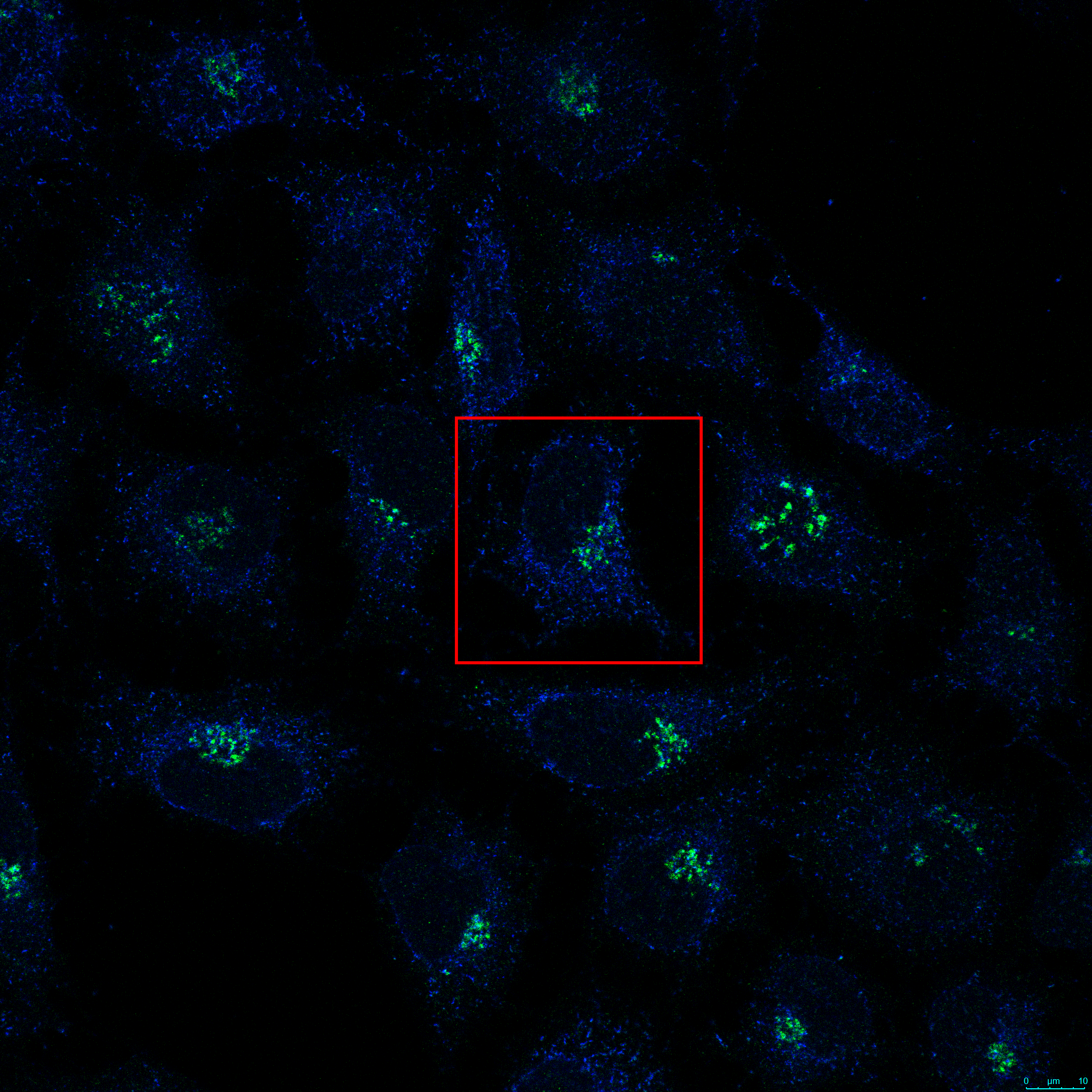

Supplement: Supplementary file 6 — Source Data Fig. 6 [file 44319_2023_45_MOESM6_ESM.zip › Fig 6/Fig 6B/F6B2.tif]

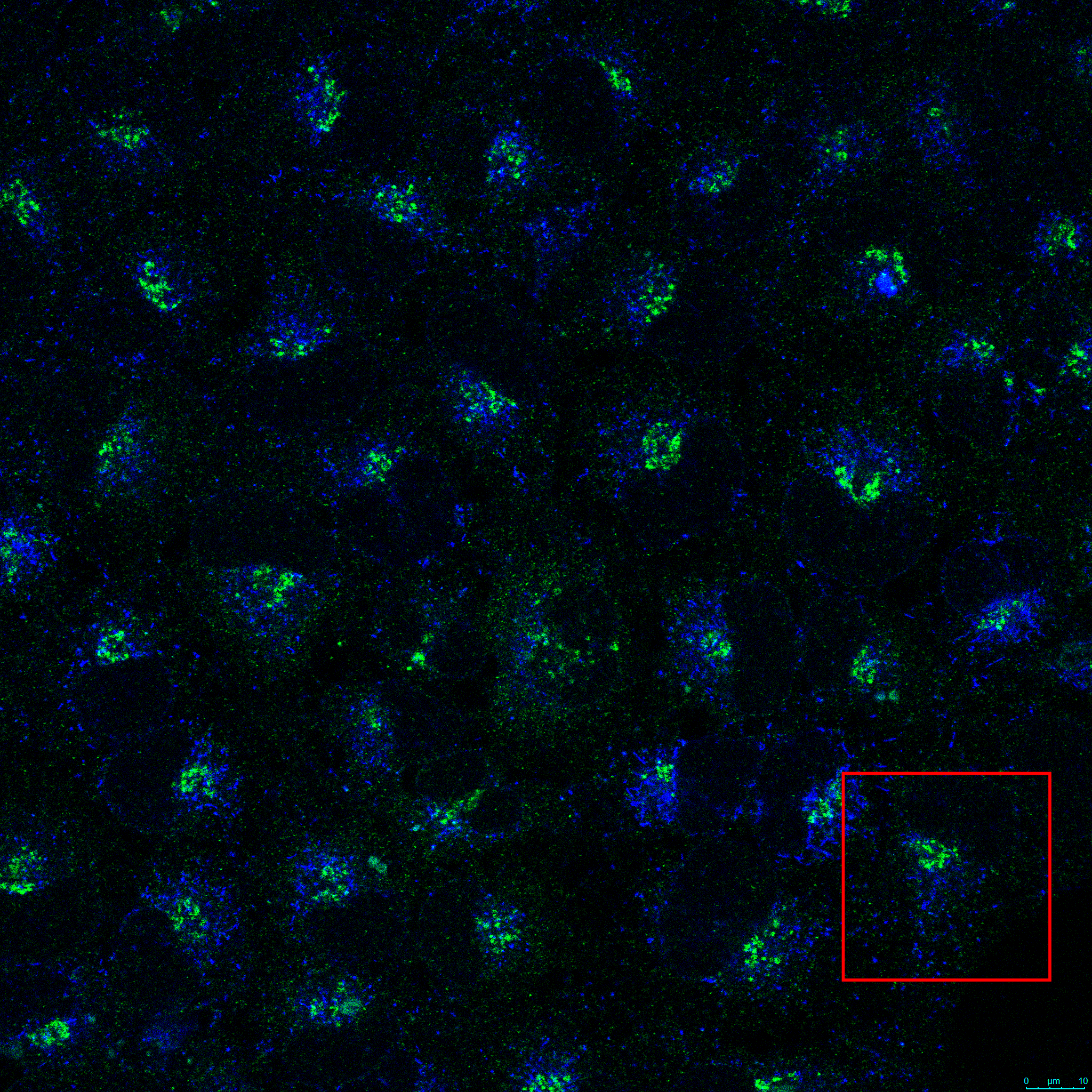

Supplement: Supplementary file 6 — Source Data Fig. 6 [file 44319_2023_45_MOESM6_ESM.zip › Fig 6/Fig 6B/F6B3.tif]

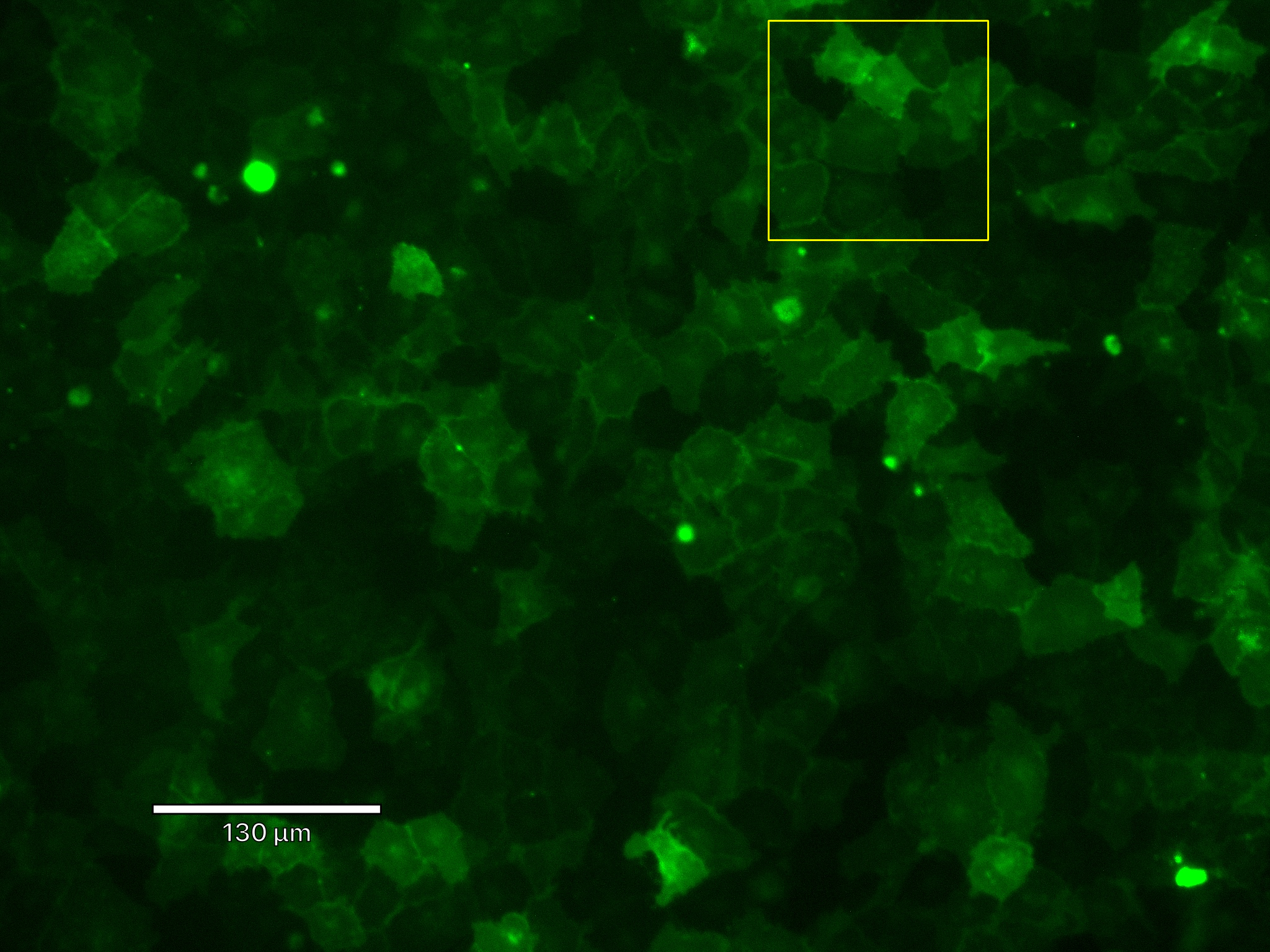

Supplement: Supplementary file 6 — Source Data Fig. 6 [file 44319_2023_45_MOESM6_ESM.zip › Fig 6/Fig 6E/F6E3_matched.png]

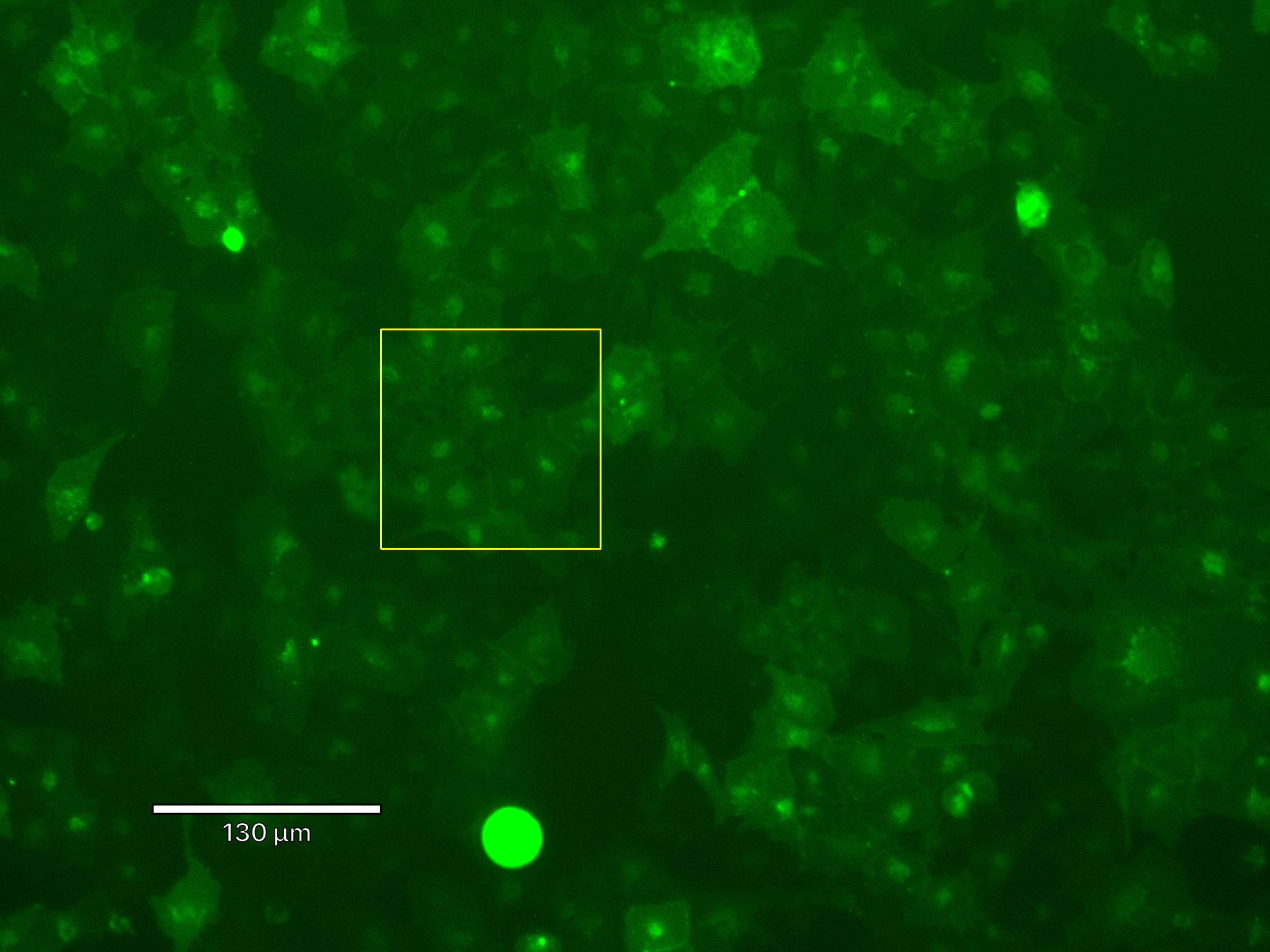

Supplement: Supplementary file 6 — Source Data Fig. 6 [file 44319_2023_45_MOESM6_ESM.zip › Fig 6/Fig 6E/F6E2_matched.png]

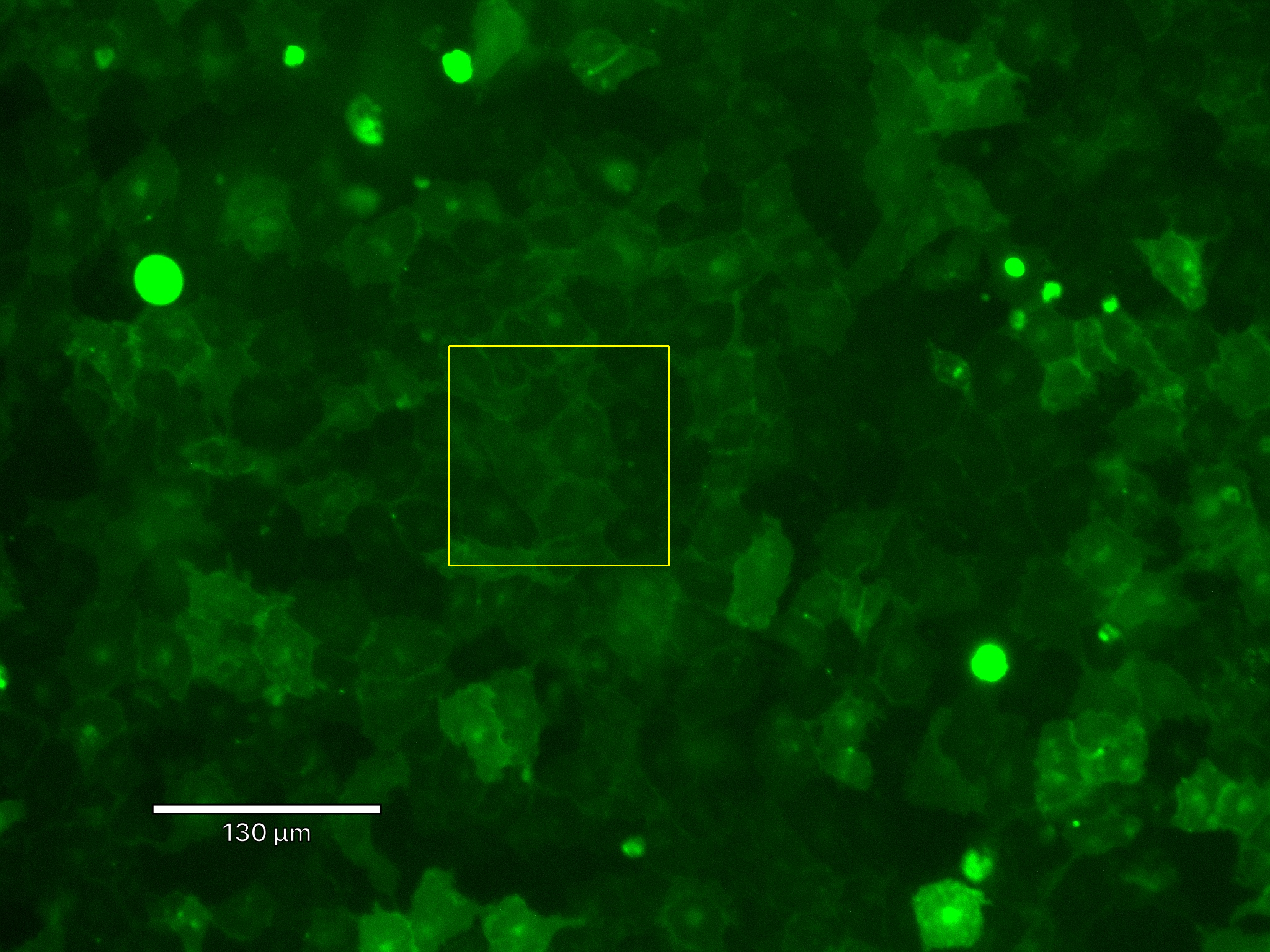

Supplement: Supplementary file 6 — Source Data Fig. 6 [file 44319_2023_45_MOESM6_ESM.zip › Fig 6/Fig 6E/F6E4_matched.png]

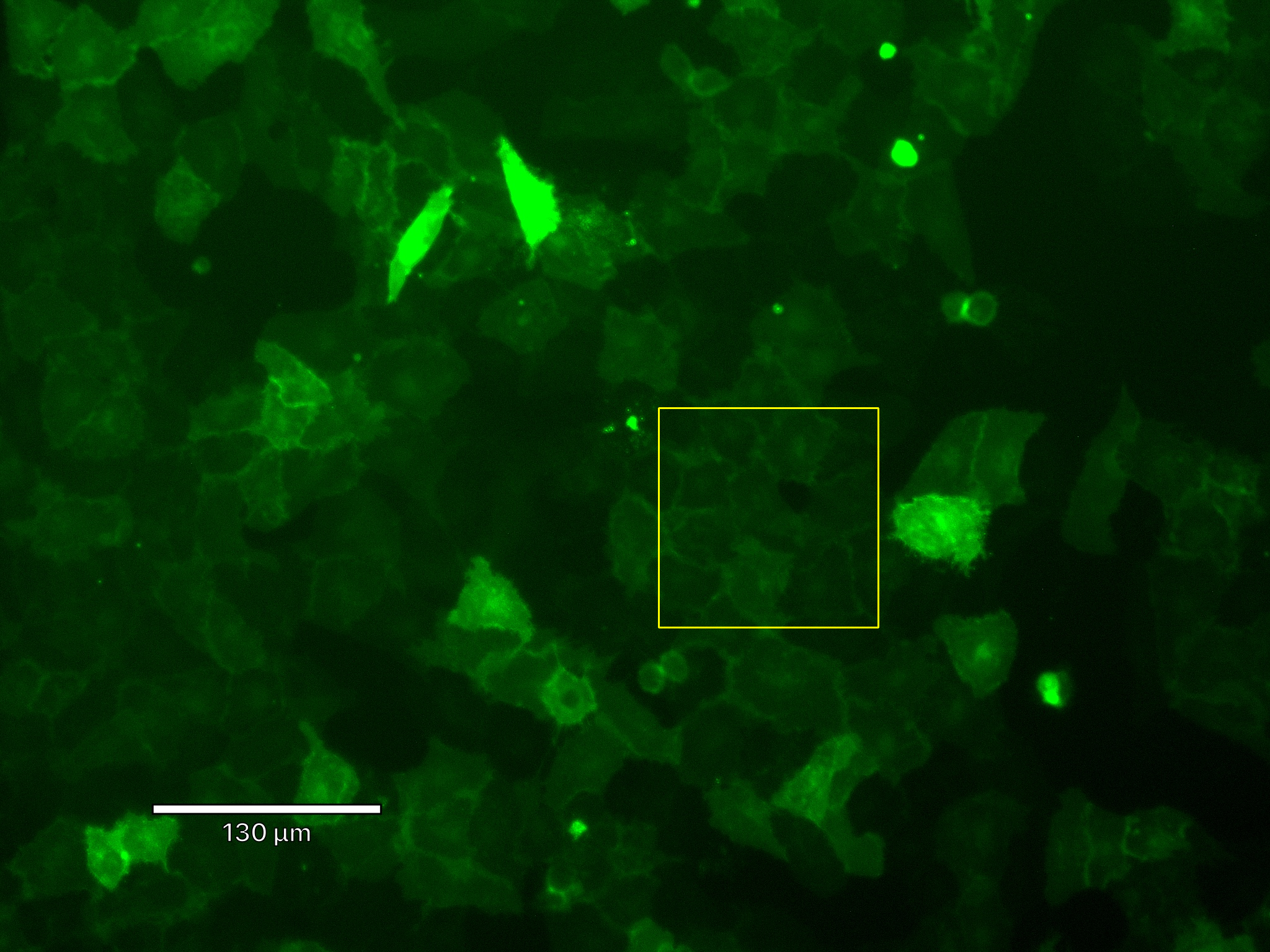

Supplement: Supplementary file 6 — Source Data Fig. 6 [file 44319_2023_45_MOESM6_ESM.zip › Fig 6/Fig 6E/F6E1_matched.png]

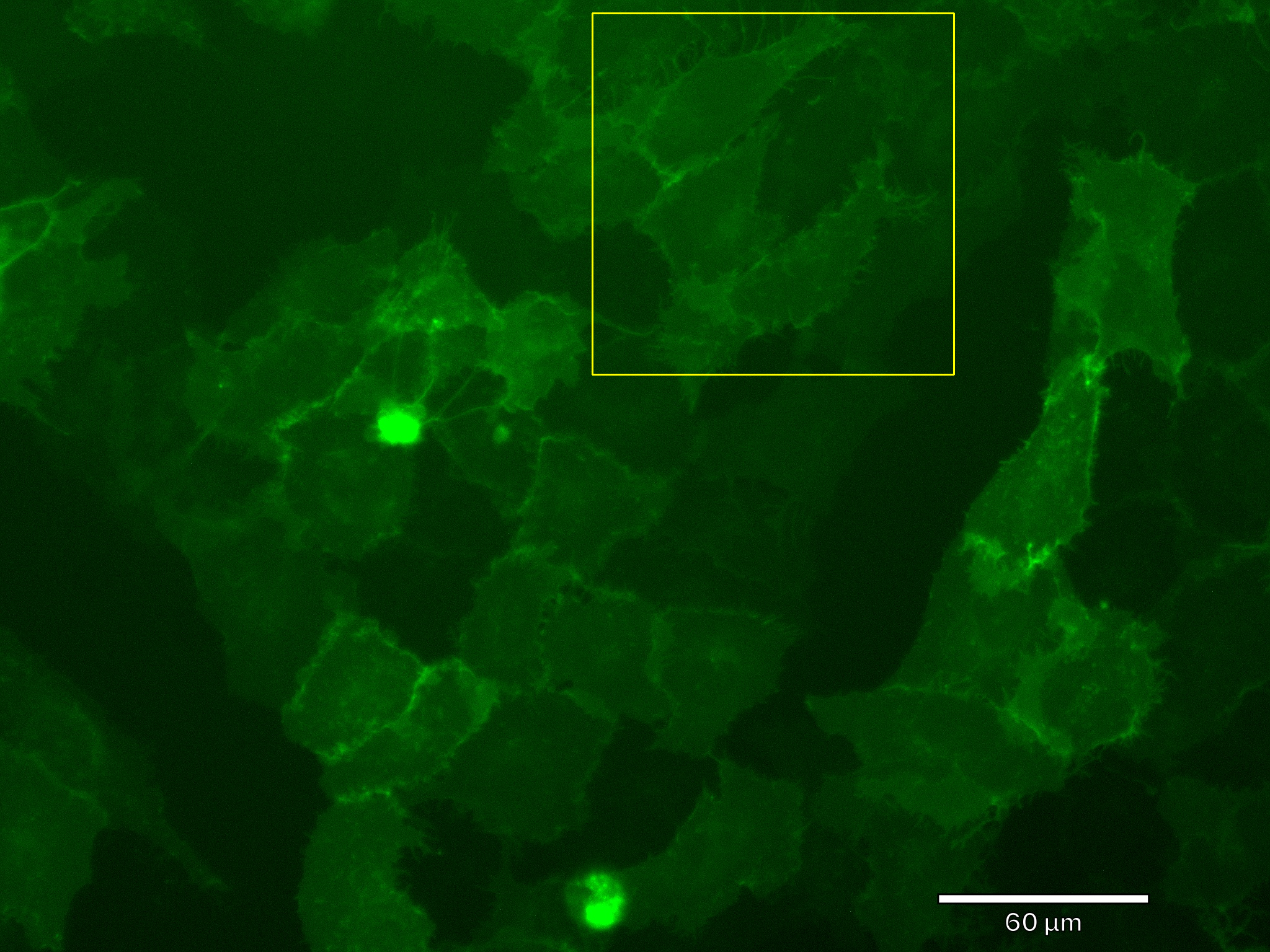

Supplement: Supplementary file 7 — Source Data Fig. 7 [file 44319_2023_45_MOESM7_ESM.zip › Fig 7/Fig 7A/F7A1_matched.png]

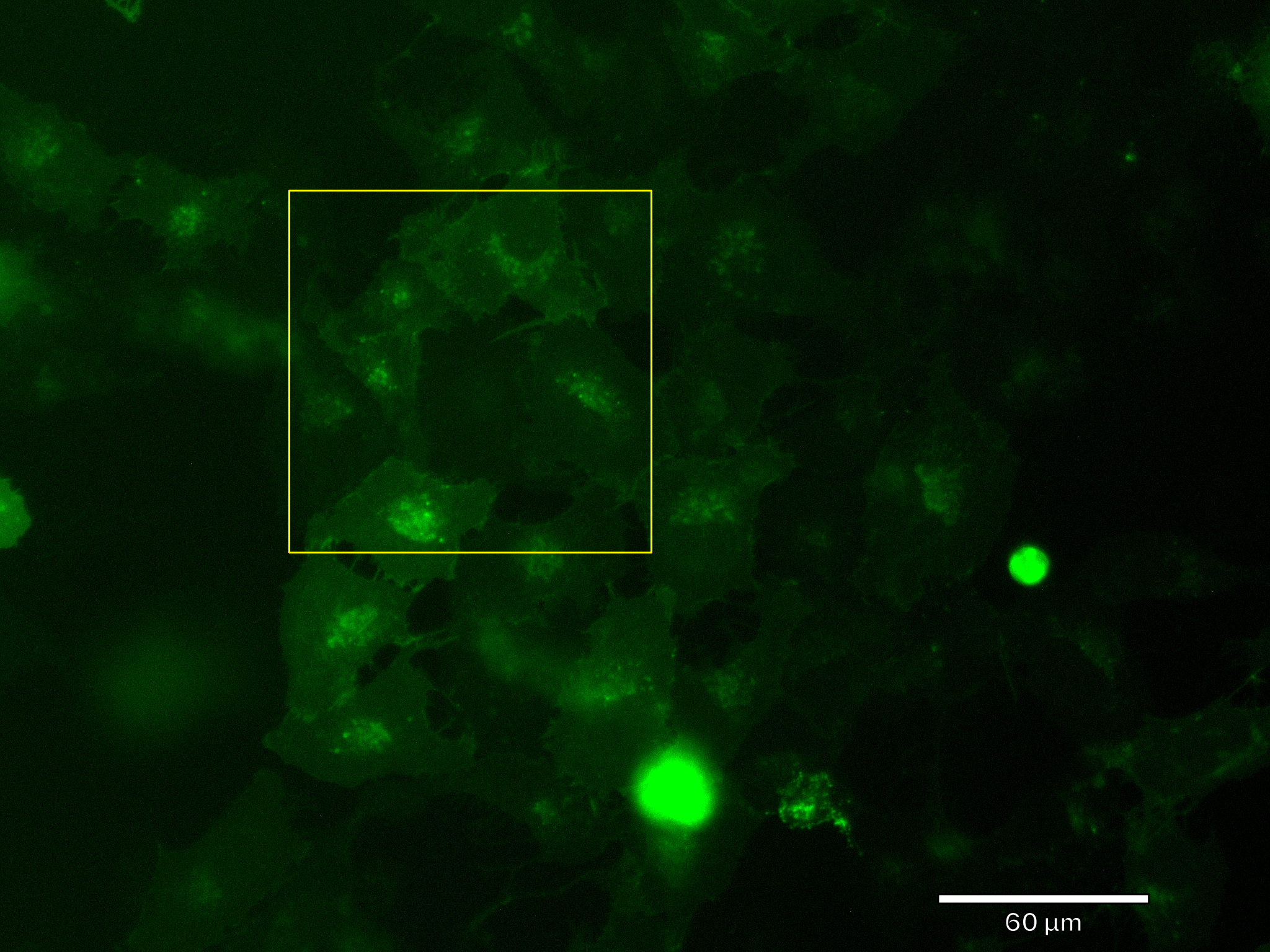

Supplement: Supplementary file 7 — Source Data Fig. 7 [file 44319_2023_45_MOESM7_ESM.zip › Fig 7/Fig 7A/F7A2_matched.png]

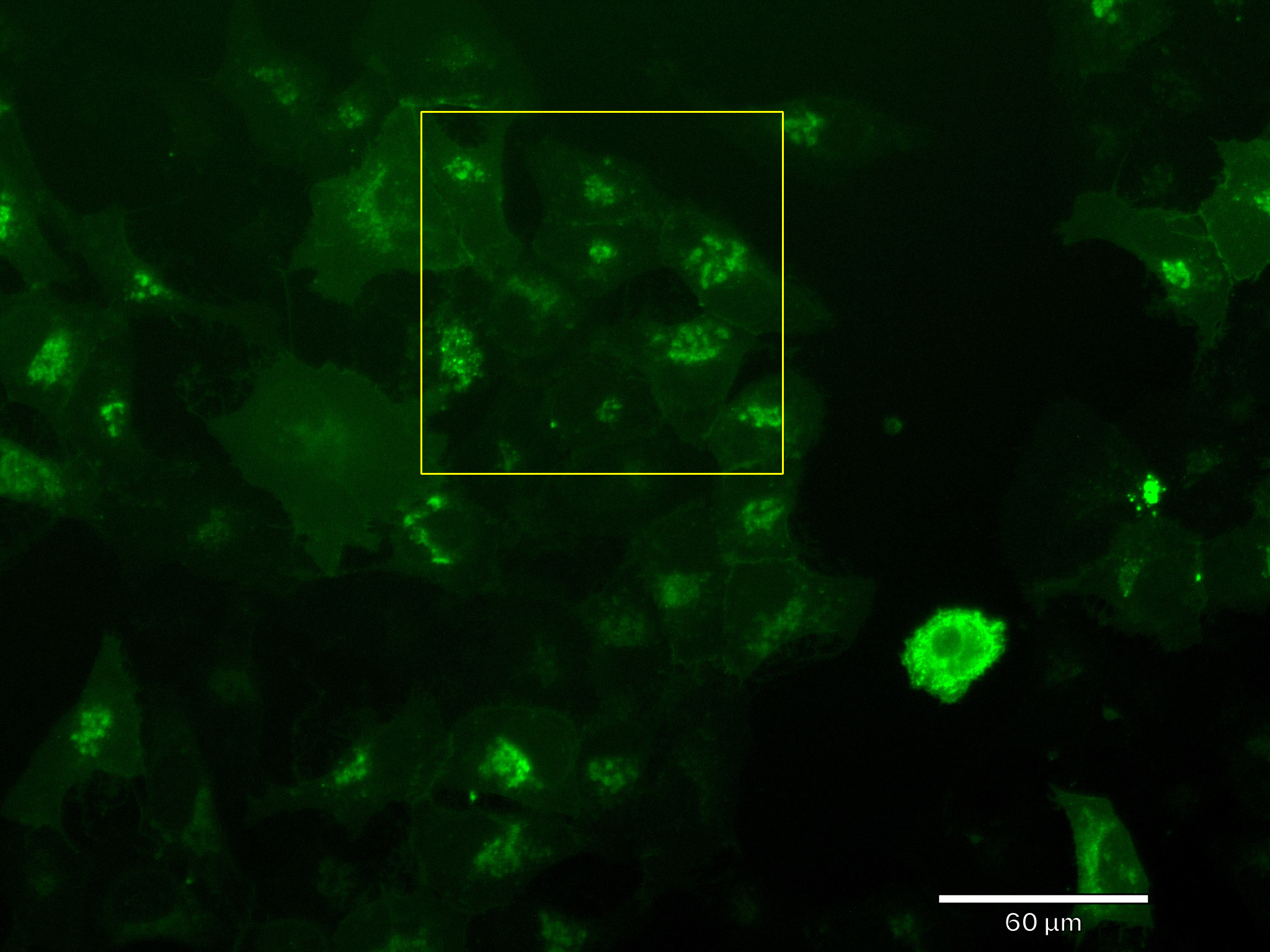

Supplement: Supplementary file 7 — Source Data Fig. 7 [file 44319_2023_45_MOESM7_ESM.zip › Fig 7/Fig 7A/F7A3_matched.png]

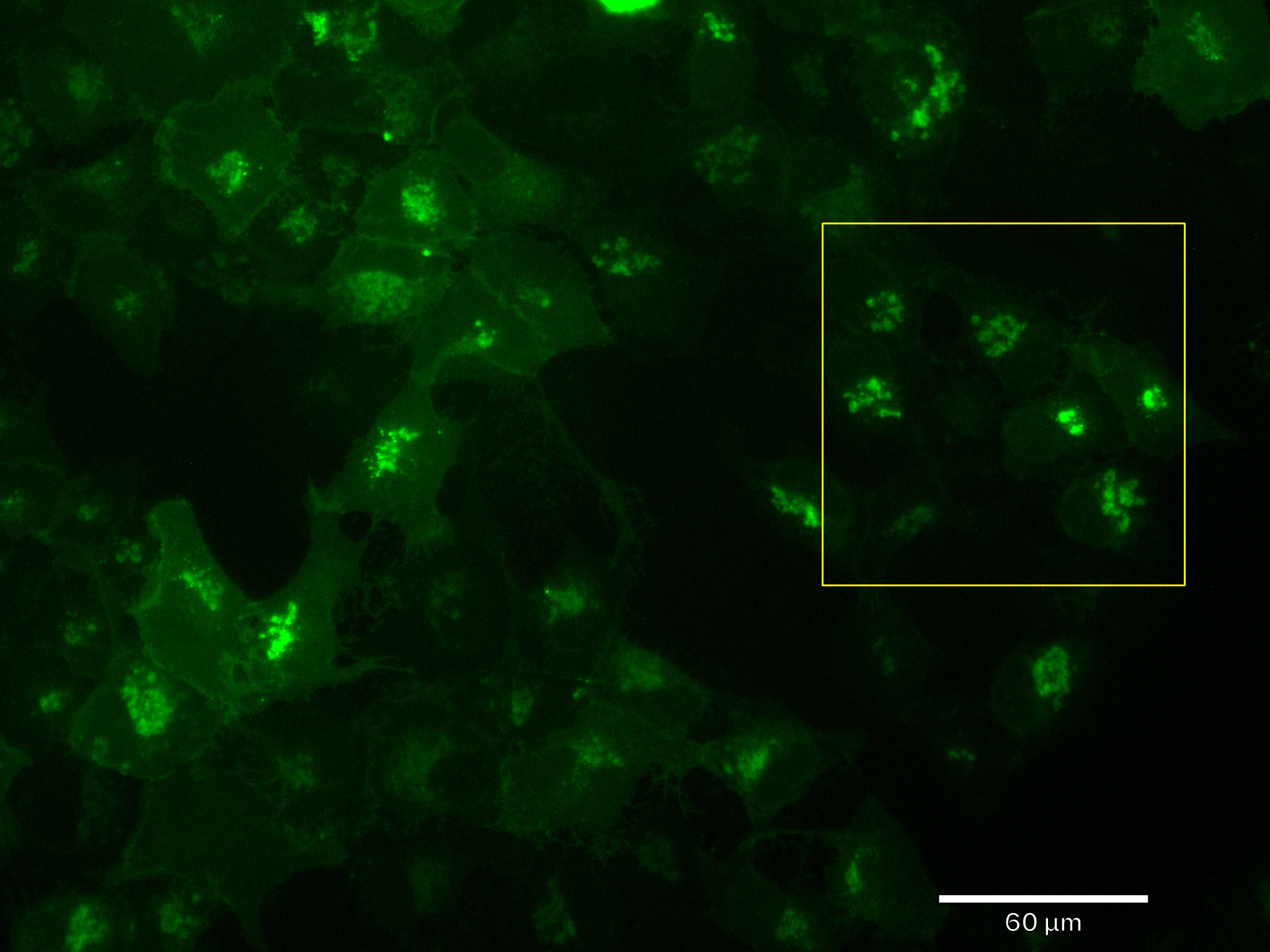

Supplement: Supplementary file 7 — Source Data Fig. 7 [file 44319_2023_45_MOESM7_ESM.zip › Fig 7/Fig 7A/F7A4_matched.png]

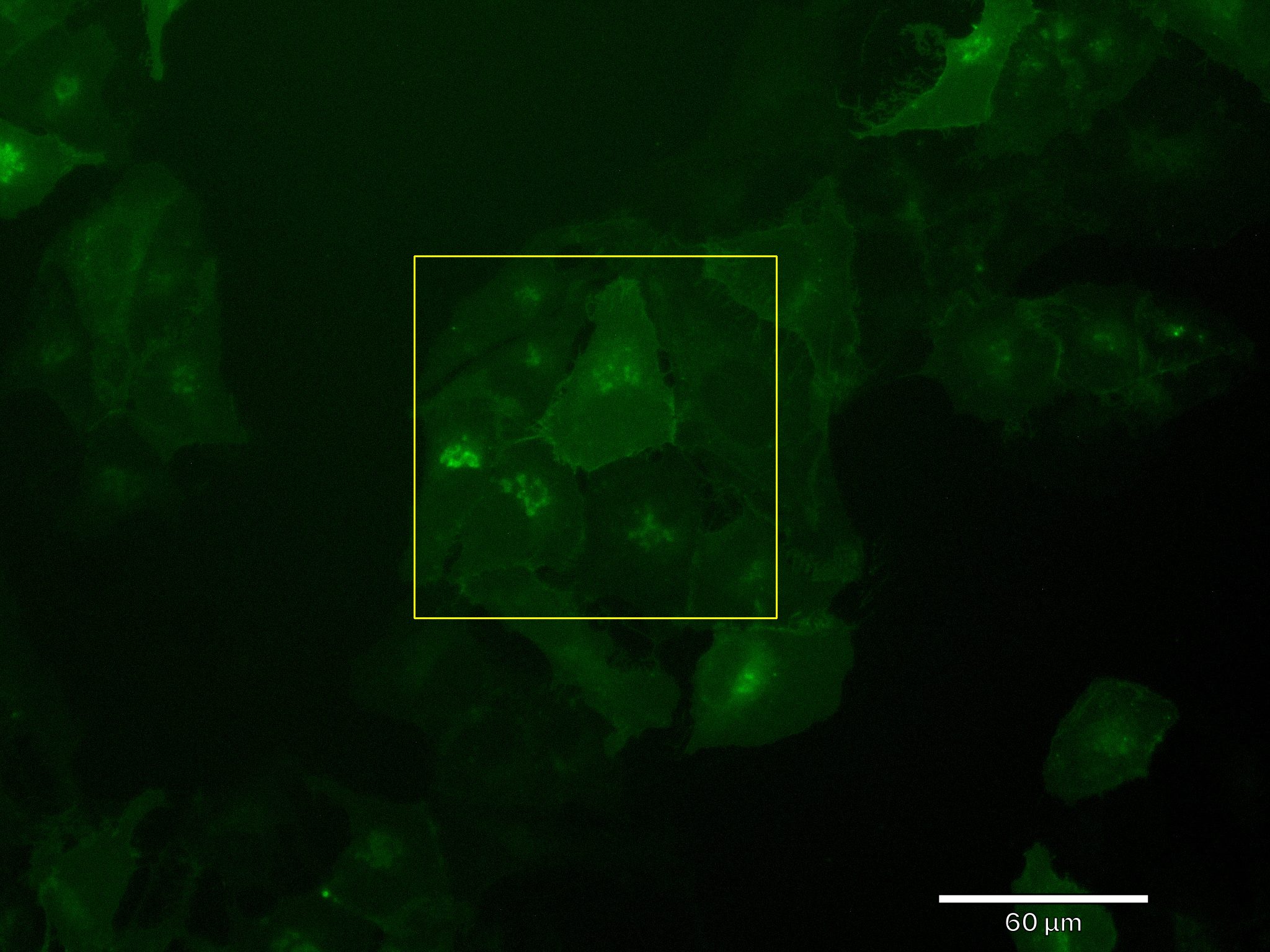

Supplement: Supplementary file 7 — Source Data Fig. 7 [file 44319_2023_45_MOESM7_ESM.zip › Fig 7/Fig 7A/F7A5_matched.png]

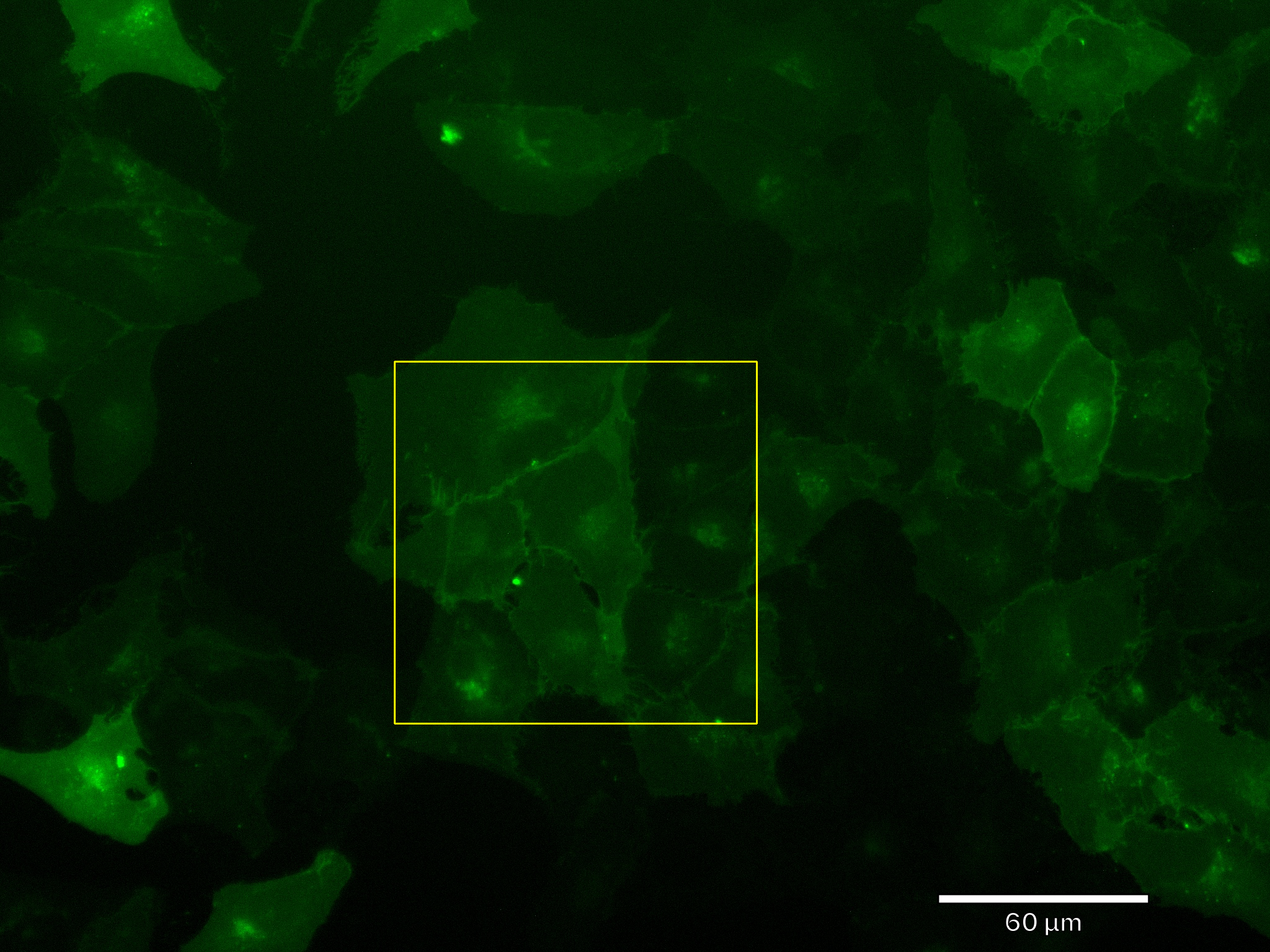

Supplement: Supplementary file 7 — Source Data Fig. 7 [file 44319_2023_45_MOESM7_ESM.zip › Fig 7/Fig 7A/F7A6_matched.png]

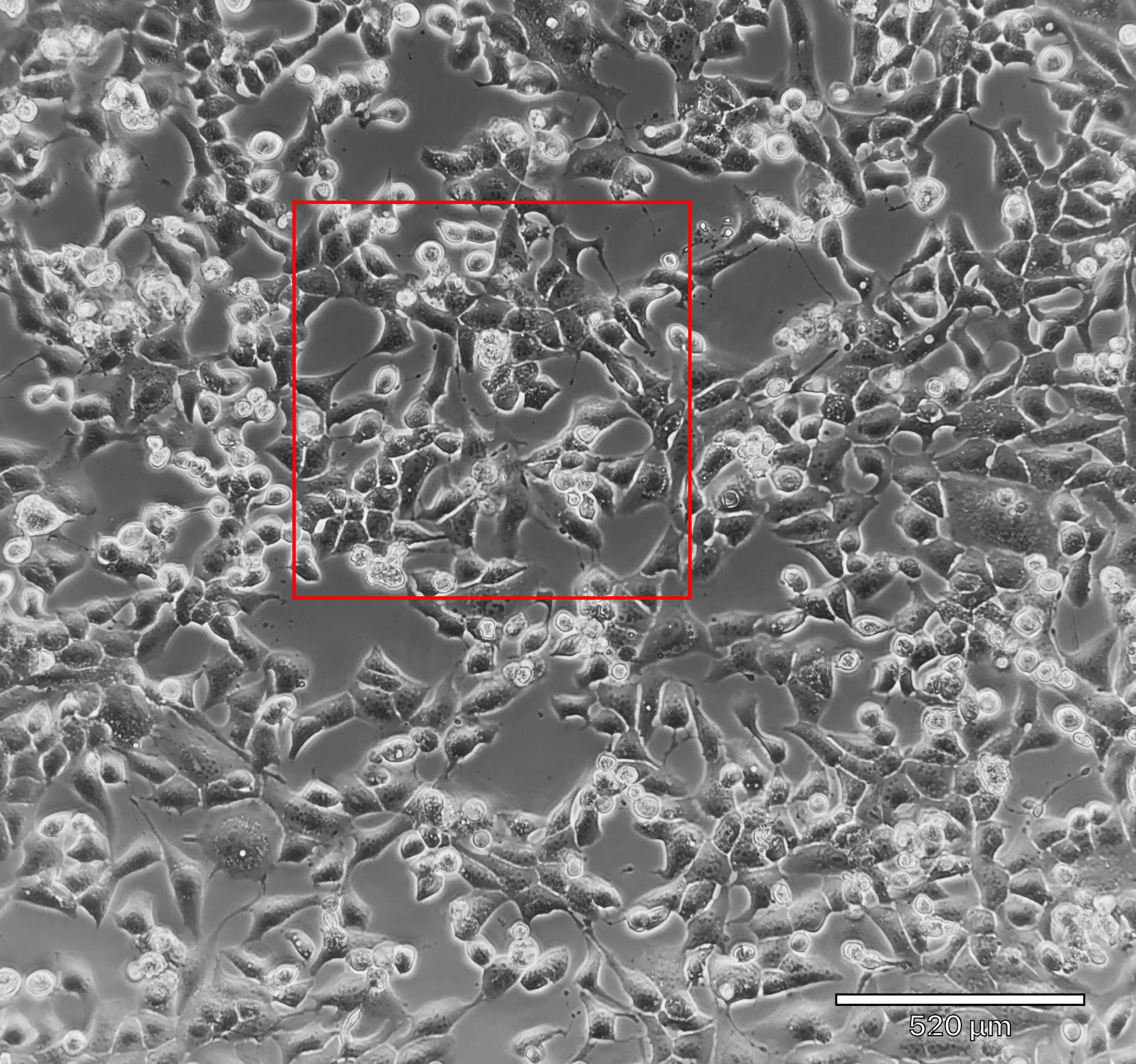

Supplement: Supplementary file 7 — Source Data Fig. 7 [file 44319_2023_45_MOESM7_ESM.zip › Fig 7/Fig 7D/F7D1 + DMSO.tif]

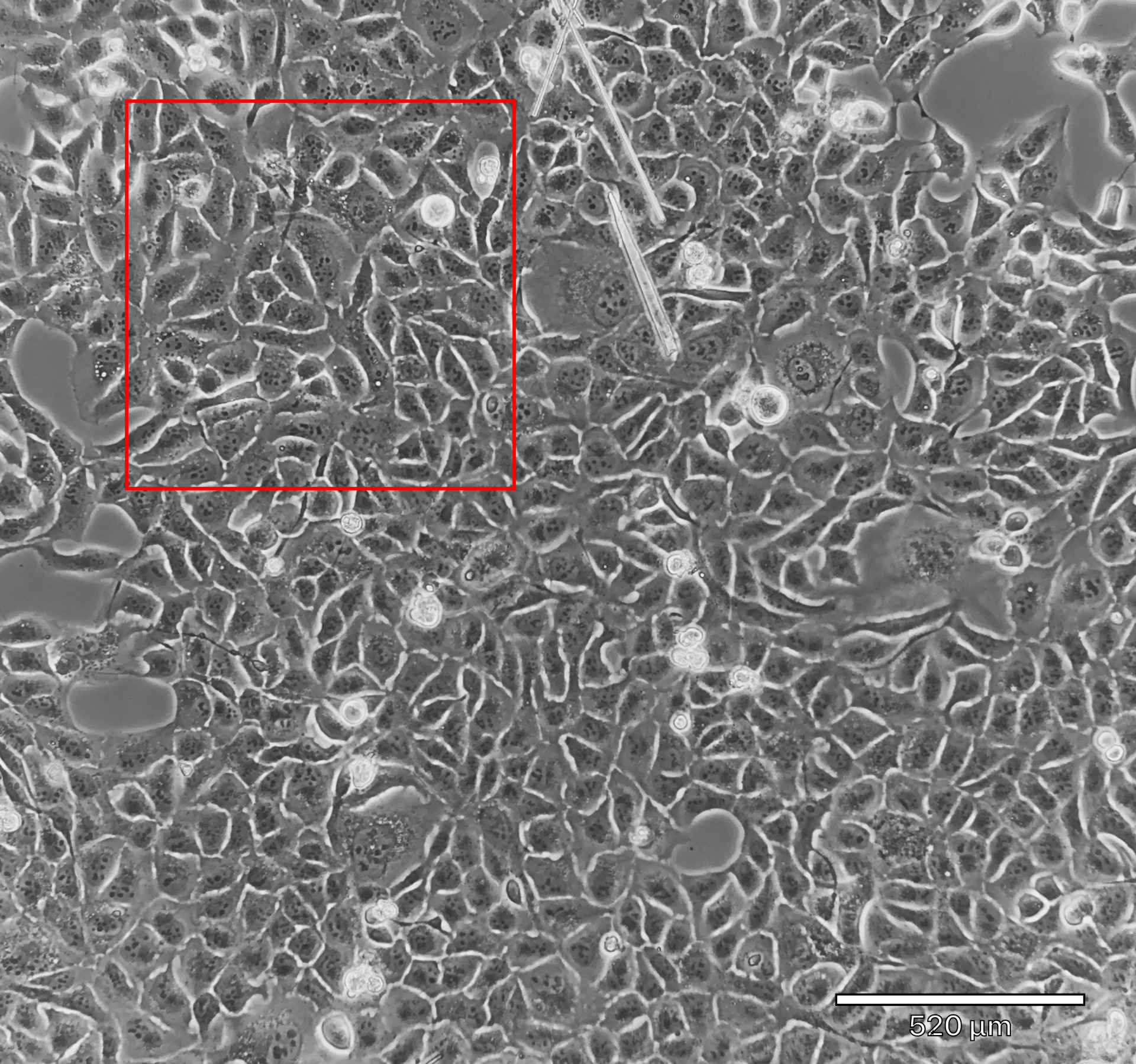

Supplement: Supplementary file 7 — Source Data Fig. 7 [file 44319_2023_45_MOESM7_ESM.zip › Fig 7/Fig 7D/F7D2 + C53.tif]

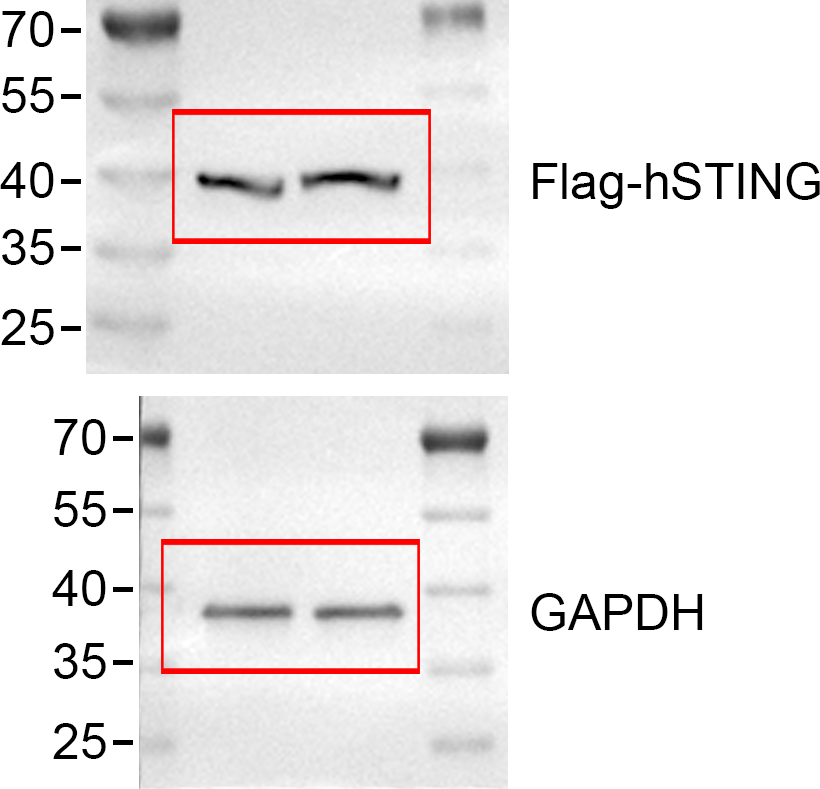

Supplement: Supplementary file 7 — Source Data Fig. 7 [file 44319_2023_45_MOESM7_ESM.zip › Fig 7/Fig 7G/Fig 7G.tif]

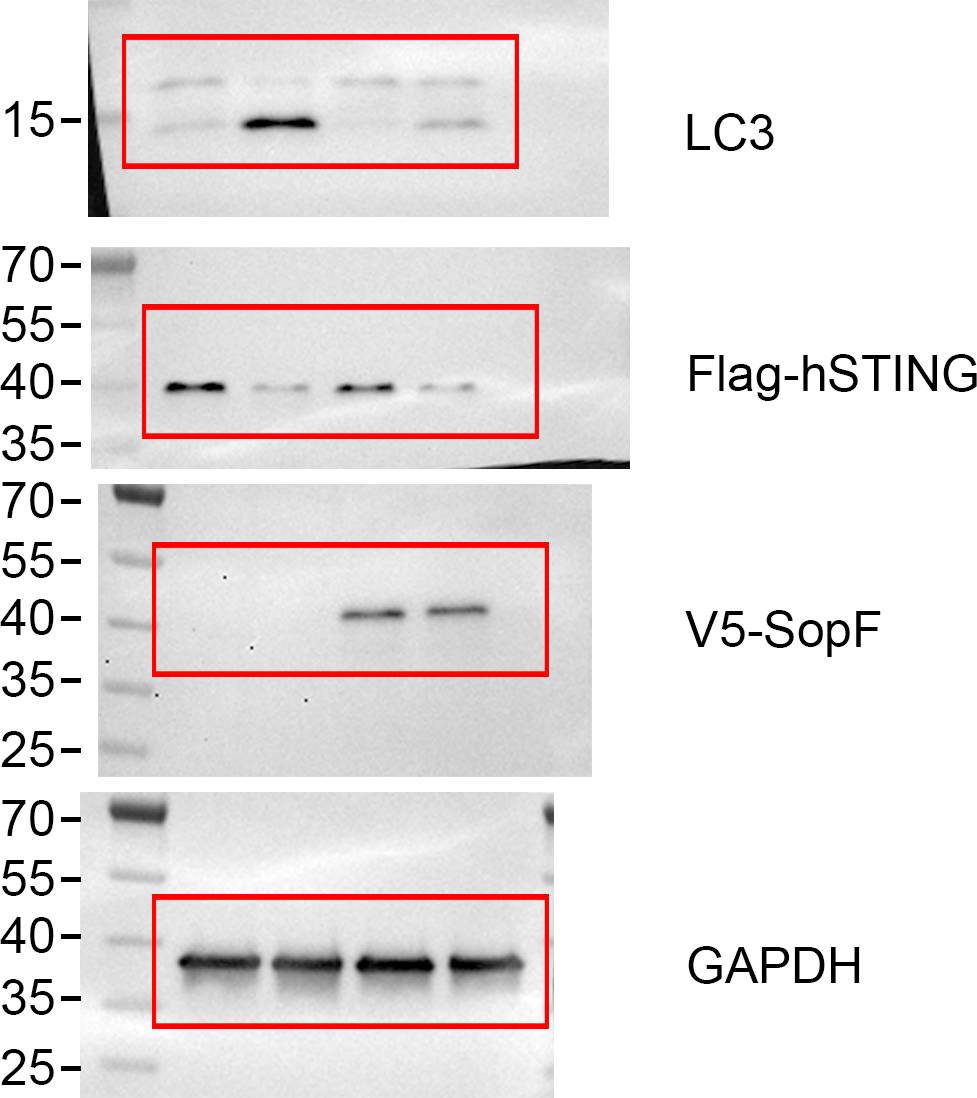

Supplement: Supplementary file 7 — Source Data Fig. 7 [file 44319_2023_45_MOESM7_ESM.zip › Fig 7/Fig 7H/Fig 7H.tif]

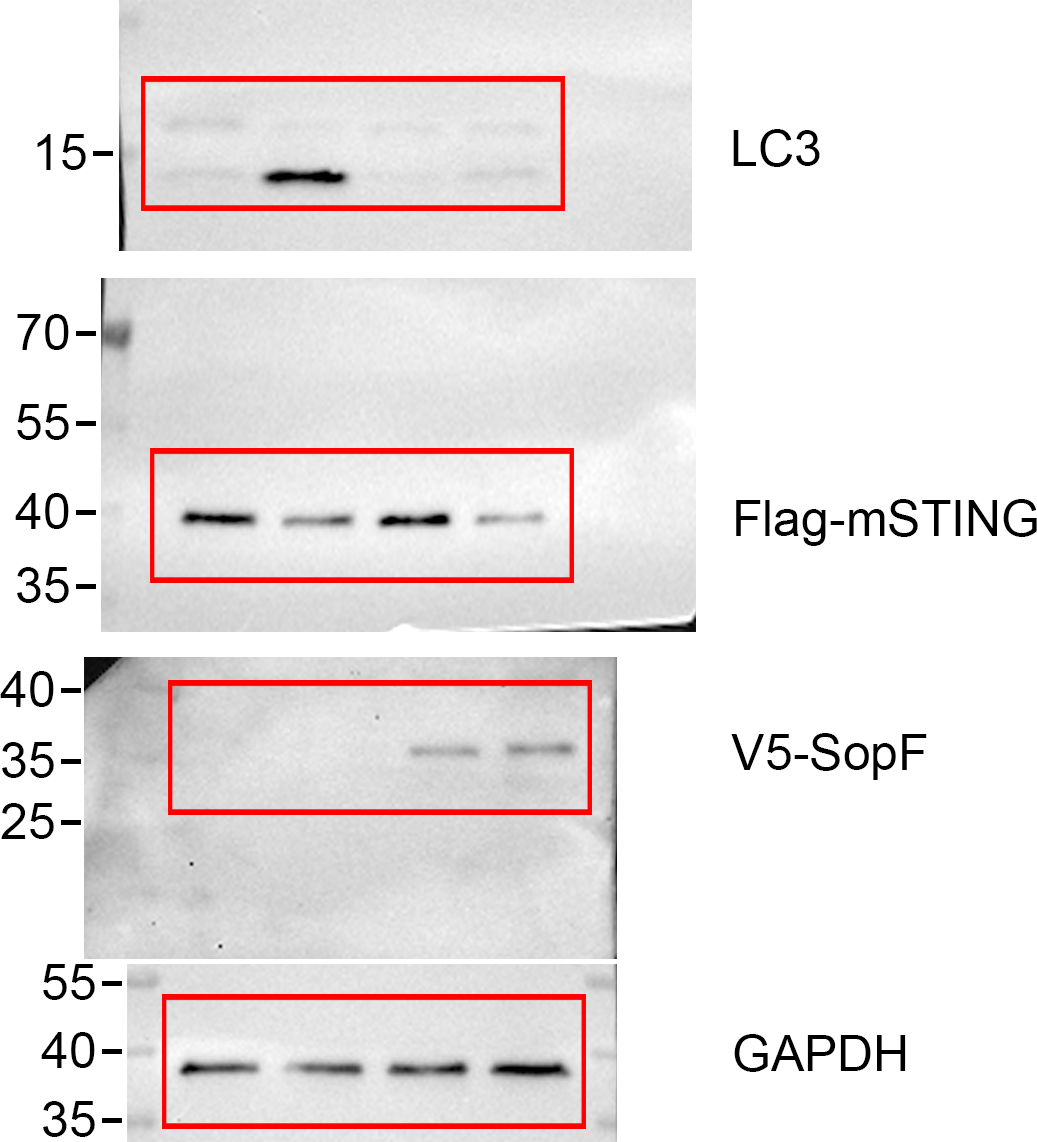

Supplement: Supplementary file 7 — Source Data Fig. 7 [file 44319_2023_45_MOESM7_ESM.zip › Fig 7/Fig 7I/Fig 7I.tif]

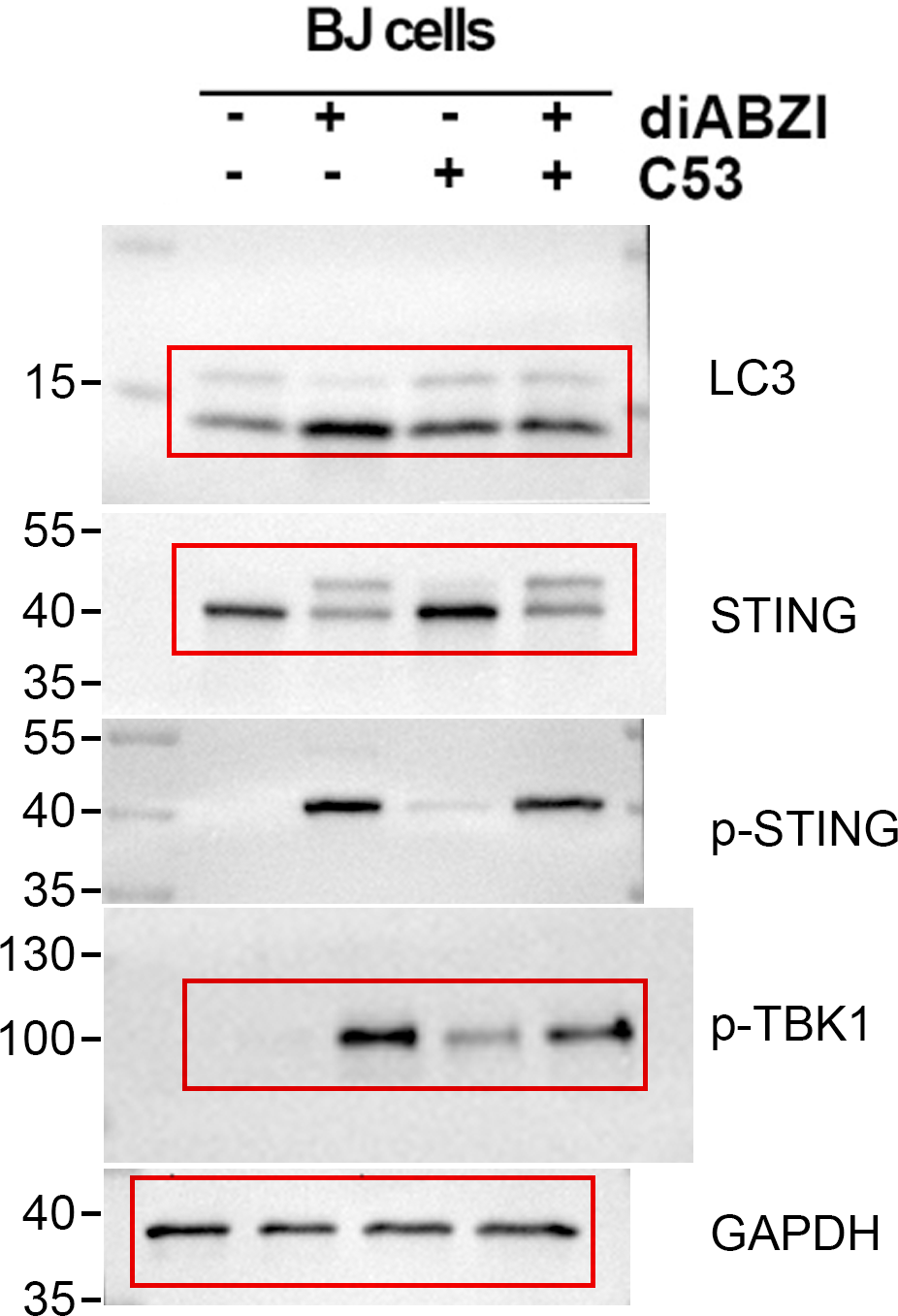

Supplement: Supplementary file 8 — EV Figures Source Data [file 44319_2023_45_MOESM8_ESM.zip › Fig EV1/Fig EV1A/western.tif]

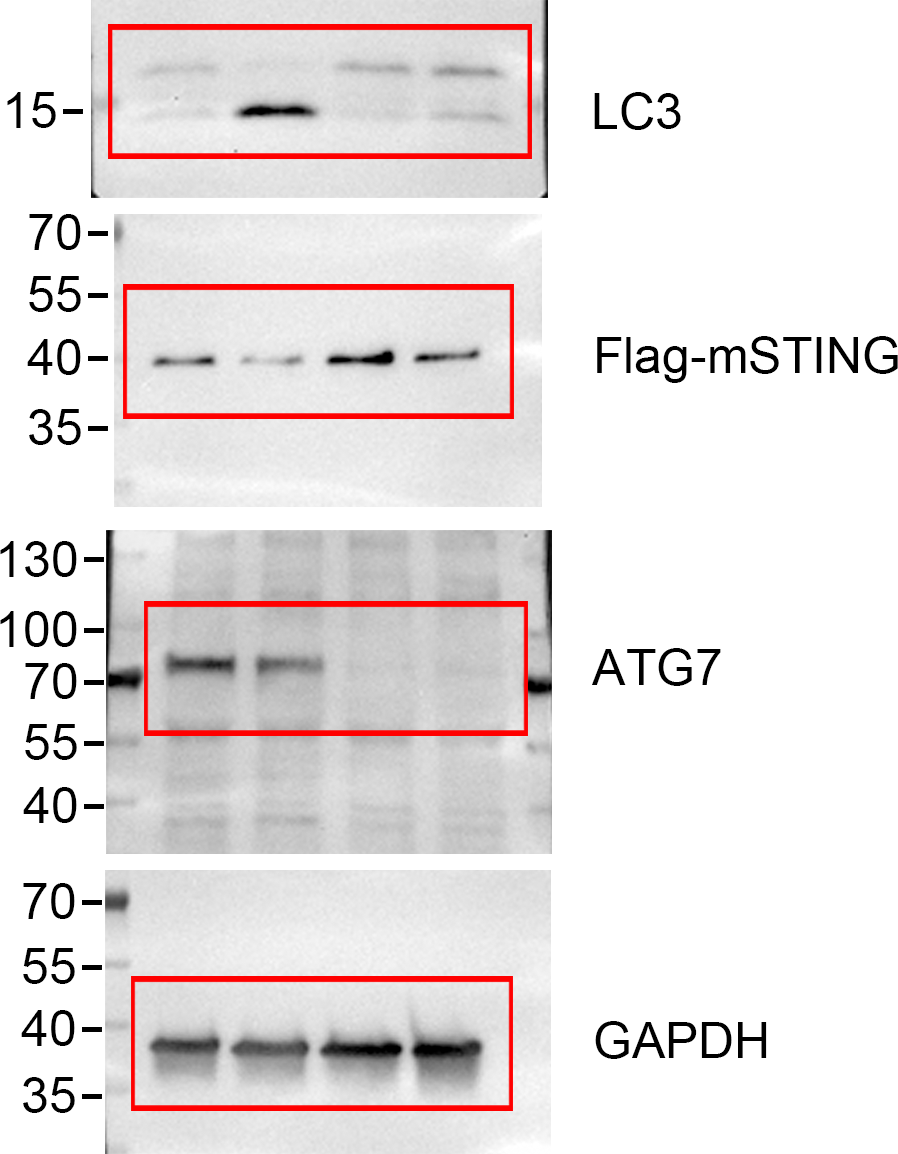

Supplement: Supplementary file 8 — EV Figures Source Data [file 44319_2023_45_MOESM8_ESM.zip › Fig EV5/EV5D/EV5D.tif]

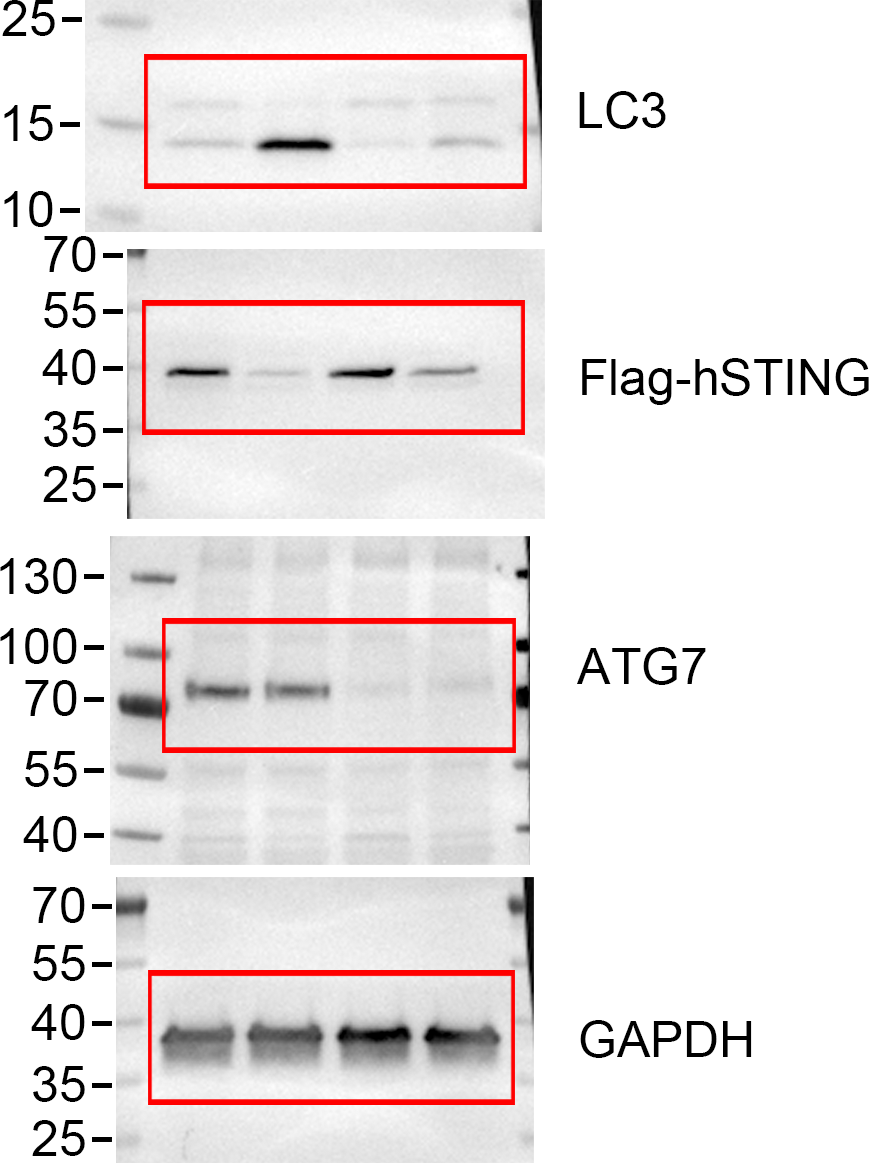

Supplement: Supplementary file 8 — EV Figures Source Data [file 44319_2023_45_MOESM8_ESM.zip › Fig EV5/EV5C/EV5C.tif]
