# Supplementary material for: A conserved ion channel function of STING mediates noncanonical autophagy and cell death
Source: EMBO Rep. 2024 Jan 2;25(2):10. doi: 10.1038/s44319-023-00045-x (PMC10897221; doi:10.1038/s44319-023-00045-x)
Supplement: Supplementary file 10 — Expanded View Figures [file 44319_2023_45_MOESM10_ESM.pdf]

Expanded View Figures

Figure EV1. STING traffics through the Golgi, causing morphological changes of the Golgi stacks.

(**A**) Left, compound C53 inhibits diABZI-induced LC3 lipidation in BJ cells without blocking TBK1 signaling and STING phosphorylation. BJ cells treated as indicated were harvested 2 h after treatment for western blot. Right, quantification of the relative levels of LC3-II normalized to GAPDH in (**A**). Mean ± SEM; $n = 3$. (**B**) STING trafficking triggers morphological changes of the Golgi stacks 30 min after cGAMP stimulation in U2OS cells. Note brighter and swelled TGN marker Golgin97 at 30 min. Monoclonal U2OS Flag-hSTING cells were stimulated with 1 μM cGAMP and fixed at indicated time points for the co-staining of endogenous *cis*- and *trans*-Golgi markers GM130 and Golgin97, respectively. (**C**, **D**) Quantification of the colocalization between GM130 and Golgin97 in (**B**). Mean ± SEM; $n = 68$, 96, 50, and 86 random cells for 0′, 30′, 60′, and 90′, respectively. (**E**) STING trafficking triggers morphological changes of the Golgi stacks 30 min after cGAMP stimulation in HT1080 cells expressing endogenous STING. Note brighter and swelled TGN marker Golgin97 as well as increased overlap between GM130 and Golgin97 at 30 min. HT1080 cells were stimulated with 1 μM cGAMP and fixed at indicated time points for the co-staining of endogenous cis- and *trans*-Golgi markers GM130 and Golgin97, respectively. (**F**) STING trafficking causes TGN46 budding onto Golgi-derived vesicles which partially colocalize with post-Golgi STING vesicles. Monoclonal U2OS Flag-hSTING cells were stimulated with 1 μM cGAMP and fixed at indicated time points for the co-staining of STING and TGN46. Note, TGN46 was found out of the Golgi stacks at 30 min before STING budded out from TGN. (**G**) diABZI stimulates STING trafficking through the Golgi body. Monoclonal U2OS Flag-hSTING cells were stimulated with 1 μM diABZI and fixed at indicated time points for the co-staining of STING and Golgin97. (**H**) Quantification of the colocalization between STING and Golgin97 in (**G**). Mean ± SEM; $n = 57$, 65, 76, 74, and 66 random cells for 0′, 30′, 60′, 120′, and 180′, respectively. Data Information: Bar, 10 μm for all cell imaging panels. Statistical significance was determined by unpaired, two-tailed t tests for all quantifications. Source data are available online for this figure.

© The Author(s)

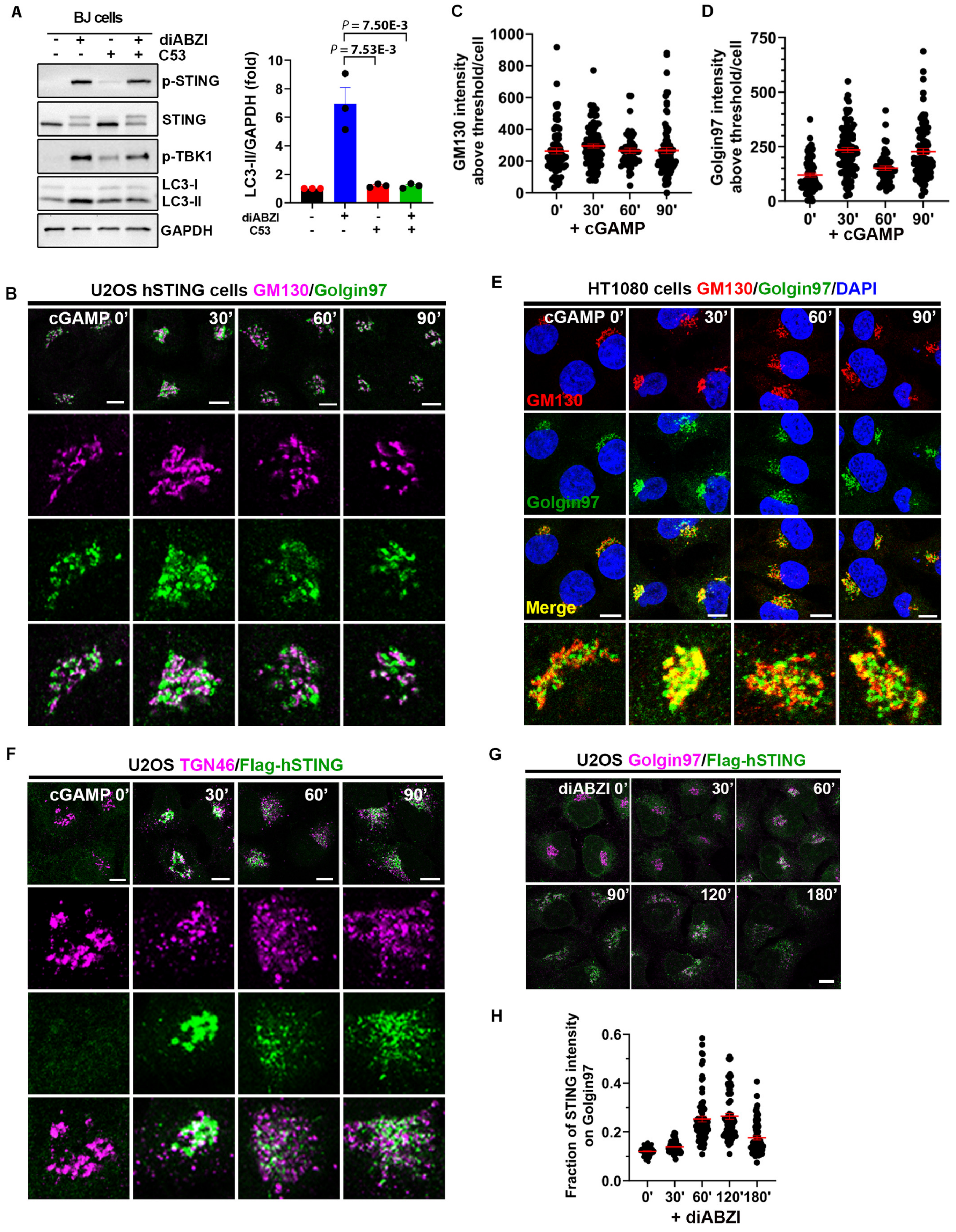
A
BJ cells
diABZI
C53
p-STING
STING
p-TBK1
LC3-I
LC3-II
GAPDH
P = 7.50E-3
P = 7.53E-3
LC3-II/GAPDH (fold)
diABZI
C53
C
GM130 intensity above threshold/cell
0' 30' 60' 90'
+ cGAMP
D
Golgin97 intensity above threshold/cell
0' 30' 60' 90'
+ cGAMP
B
U2OS hSTING cells GM130/Golgin97
cGAMP 0'
30'
60'
90'
E
HT1080 cells GM130/Golgin97/DAPI
cGAMP 0'
30'
60'
90'
GM130
Golgin97
Merge
F
U2OS TGN46/Flag-hSTING
cGAMP 0'
30'
60'
90'
G
U2OS Golgin97/Flag-hSTING
diABZI 0'
30'
60'
90'
120'
180'
H
Fraction of STING intensity on Golgin97
0' 30' 60' 120' 180'
+ diABZI

 © The Author(s)

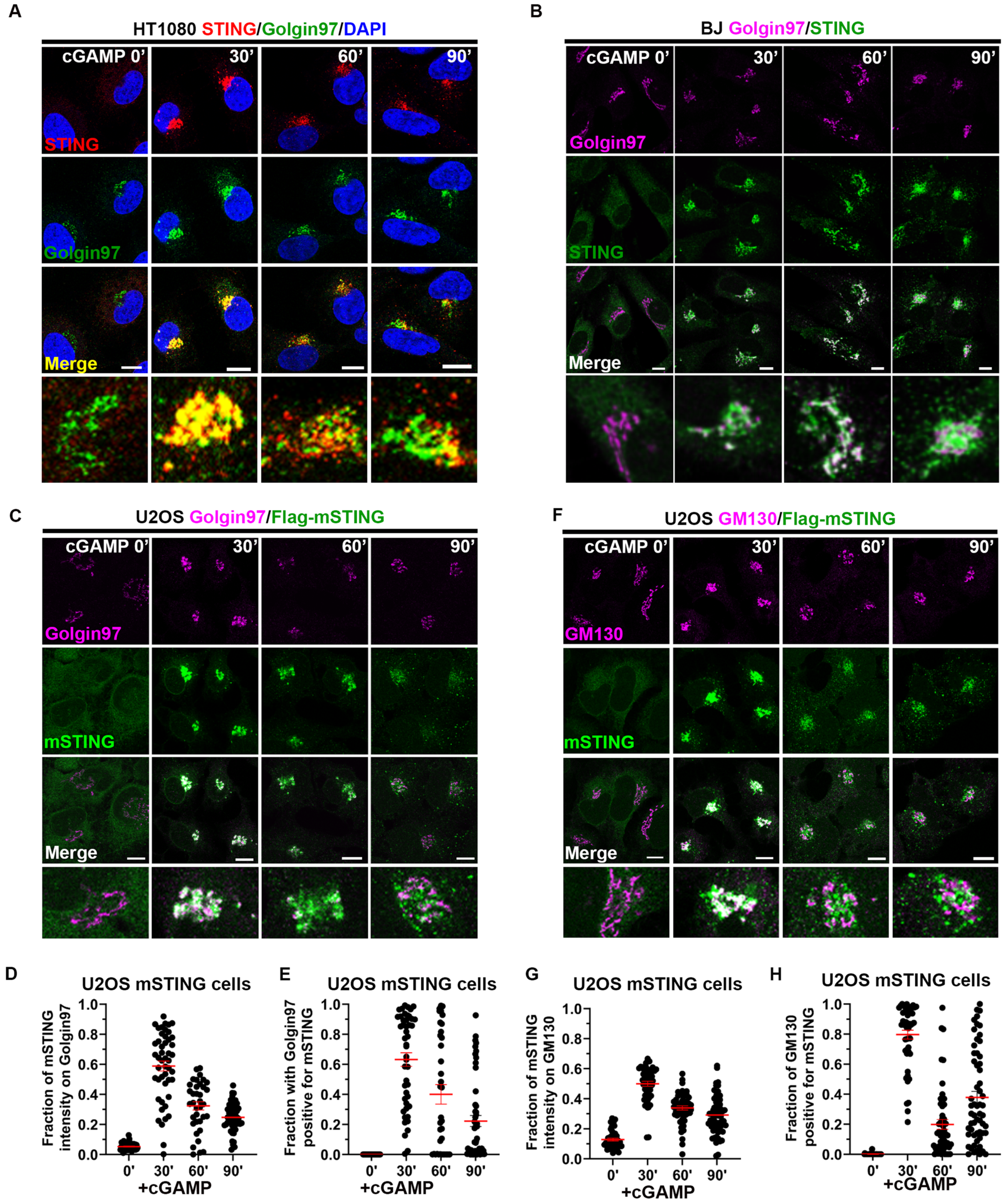
A
HT1080 STING/Golgin97/DAPI
cGAMP 0'
30'
60'
90'
STING
Golgin97
Merge
B
BJ Golgin97/STING
Golgin97
STING
Merge
C
U2OS Golgin97/Flag-mSTING
mSTING
F
U2OS GM130/Flag-mSTING
GM130
D
U2OS mSTING cells
Fraction of mSTING intensity on Golgin97
+cGAMP
E
Fraction with Golgin97 positive for mSTING
G
Fraction of mSTING intensity on GM130
H
Fraction of GM130 positive for mSTING

Figure EV2. Human and mouse STING similarly traffic through the Golgi complex.

(**A**, **B**) STING traffics through the Golgi after cGAMP stimulation in HT1080 (**A**) and BJ (**B**) cells, both expressing endogenous human STING. Cells were stimulated with 1 μM cGAMP and fixed at indicated time points for the co-staining of endogenous STING and the *trans*-Golgi marker Golgin97, respectively. Note, brighter and swelled TGN marker Golgin97 at 30 min in HT1080 cells, whereas STING accumulation at the Golgi in BJ cells appeared to peak between 30 and 60 min. (**C**) Mouse STING traffics through the *trans*-Golgi upon cGAMP binding. U2OS cells stably expressing low levels of Flag-mSTING were stimulated with 1 μM cGAMP and fixed at indicated time points for the co-staining of STING and the *trans*-Golgi marker Golgin97. (**D**, **E**) Quantification of the colocalization between STING and Golgin97 in (**C**). Mean ± SEM; $n = 49$, 48, 36, and 52 random cells for 0′, 30′, 60′, and 90′, respectively. (**F**) STING traffics through the *cis*-Golgi upon cGAMP binding. U2OS cells stably expressing low levels of Flag-mSTING were stimulated with 1 μM cGAMP and fixed at indicated time points for the co-staining of STING and the cis-Golgi marker GM130. (**G**, **H**) Quantification of the colocalization between STING and GM130 in (**F**). Mean ± SEM; $n = 41$, 50, 51, and 59 random cells for 0′, 30′, 60′, and 90′, respectively. Data Information: Bar, 10 μm for all cell imaging panels. Source data are available online for this figure.

© The Author(s)

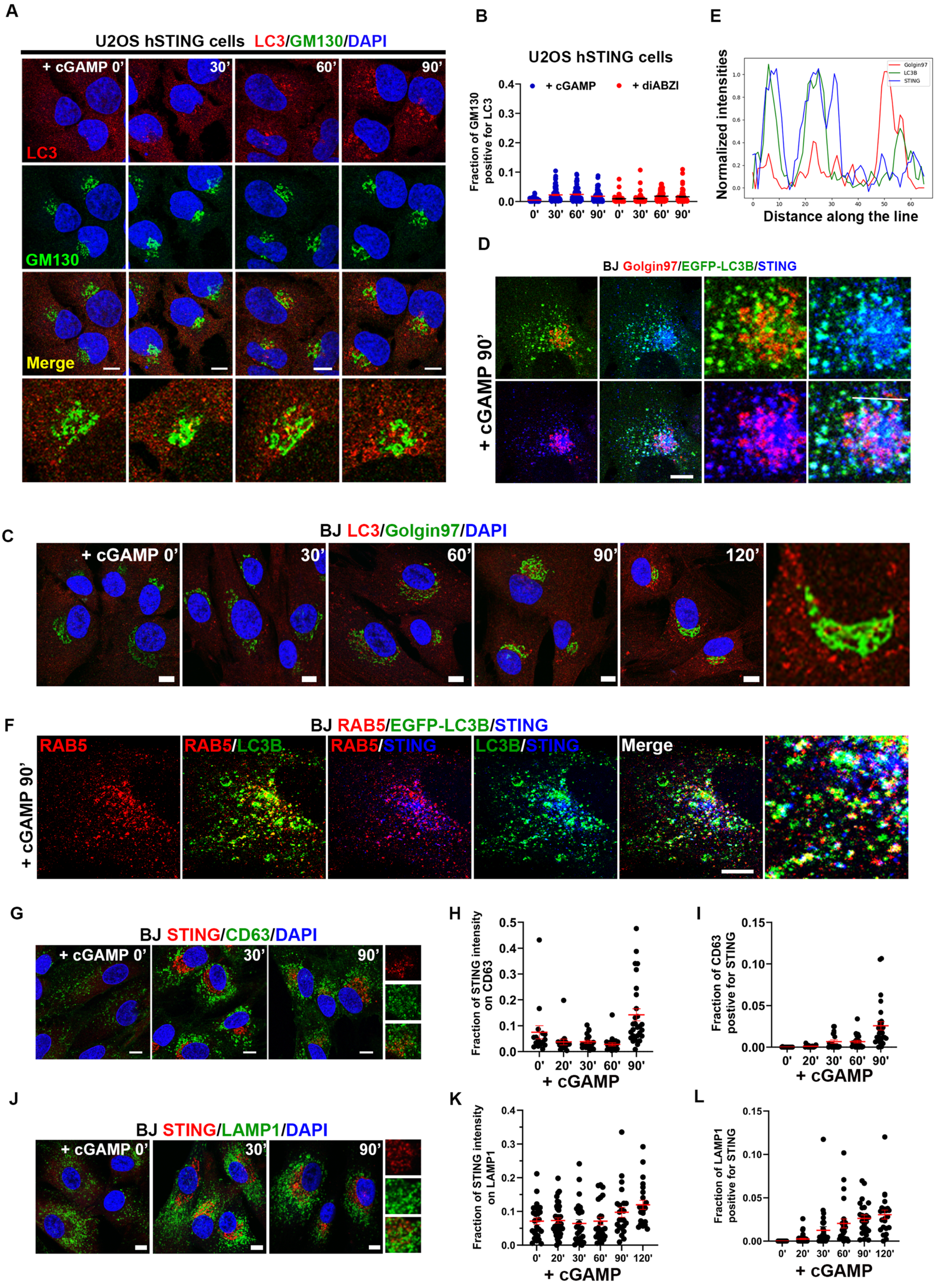
A
U2OS hSTING cells LC3/GM130/DAPI
+ cGAMP 0'
30'
60'
90'
LC3
GM130
Merge
B
U2OS hSTING cells
+ cGAMP
+ diABZI
Fraction of GM130 positive for LC3
E
Normalized intensities
Golgin97
LC3B
STING
Distance along the line
D
BJ Golgin97/EGFP-LC3B/STING
+ cGAMP 90'
C
BJ LC3/Golgin97/DAPI
+ cGAMP 0'
30'
60'
90'
120'
F
BJ RAB5/EGFP-LC3B/STING
RAB5
RAB5/LC3B
RAB5/STING
LC3B/STING
Merge
+ cGAMP 90'
G
BJ STING/CD63/DAPI
+ cGAMP 0'
30'
90'
H
Fraction of STING intensity on CD63
+ cGAMP
I
Fraction of CD63 postive for STING
+ cGAMP
J
BJ STING/LAMP1/DAPI
+ cGAMP 0'
30'
90'
K
Fraction of STING intensity on LAMP1
+ cGAMP
L
Fraction of LAMP1 positive for STING
+ cGAMP

Figure EV3. Post-Golgi STING vesicles develop endosome-like properties accompanied by LC3 lipidation.

(**A**) STING induces LC3 puncta outside of the Golgi. Monoclonal U2OS Flag-hSTING cells were stimulated with 1 μM cGAMP and fixed at indicated time points for the co-staining of endogenous LC3 and the *cis*-Golgi marker GM130. (**B**) Quantification of the colocalization between LC3 and GM130. Mean ± SEM; $n = 73, 79, 75, 86, 40, 55, 87$, and 71 random cells from left to right. (**C**) STING induces LC3 puncta outside of the Golgi in BJ cells. Cells were stimulated with 1 μM cGAMP and fixed at indicated time points for the co-staining of endogenous LC3 and the *trans*-Golgi marker Golgin97. (**D**) LC3 puncta were found on STING vesicles leaving the perinuclear vesicle clusters and were not found on Golgin97. BJ cells stably expressing EGFP-LC3B were fixed 90 min after cGAMP stimulation for immunostaining of STING and Golgin97. (**E**) EGFP-LC3B colocalizes with STING but not Golgin97. Normalized fluorescence intensities of Golgin97, EGFP-LC3B, and STING along the white line in the right bottom image of (**D**). (**F**) LC3 puncta were found on STING vesicles positive for RAB5, near the periphery of the post-Golgi vesicle clusters. BJ cells stably expressing EGFP-LC3B were fixed 90 min after cGAMP stimulation for immunostaining of STING and RAB5. (**G**) STING puncta develop a relatively low level of colocalization with the late endosome/lysosome marker CD63. BJ Cells were stimulated with 1 μM cGAMP and fixed at indicated time points for the co-staining of endogenous STING and CD63. (**H**, **I**) Quantification of the colocalization between STING and CD63 in (**G**). Mean ± SEM; $n = 16, 16, 20, 23$, and 28 random cells from left to right. (**J**) STING puncta develop a relatively low level of colocalization with the late endosome/lysosome marker LAMP1. BJ Cells were stimulated with 1 μM cGAMP and fixed at indicated time points for the co-staining of endogenous STING and CD63. (**K**, **L**) Quantification of the colocalization between STING and LAMP1 in (**J**). Mean ± SEM; $n = 27, 23, 37, 29, 24$, and 24 random cells from left to right. Data Information: Bar, 10 μm for all cell imaging panels. Source data are available online for this figure.

 © The Author(s)

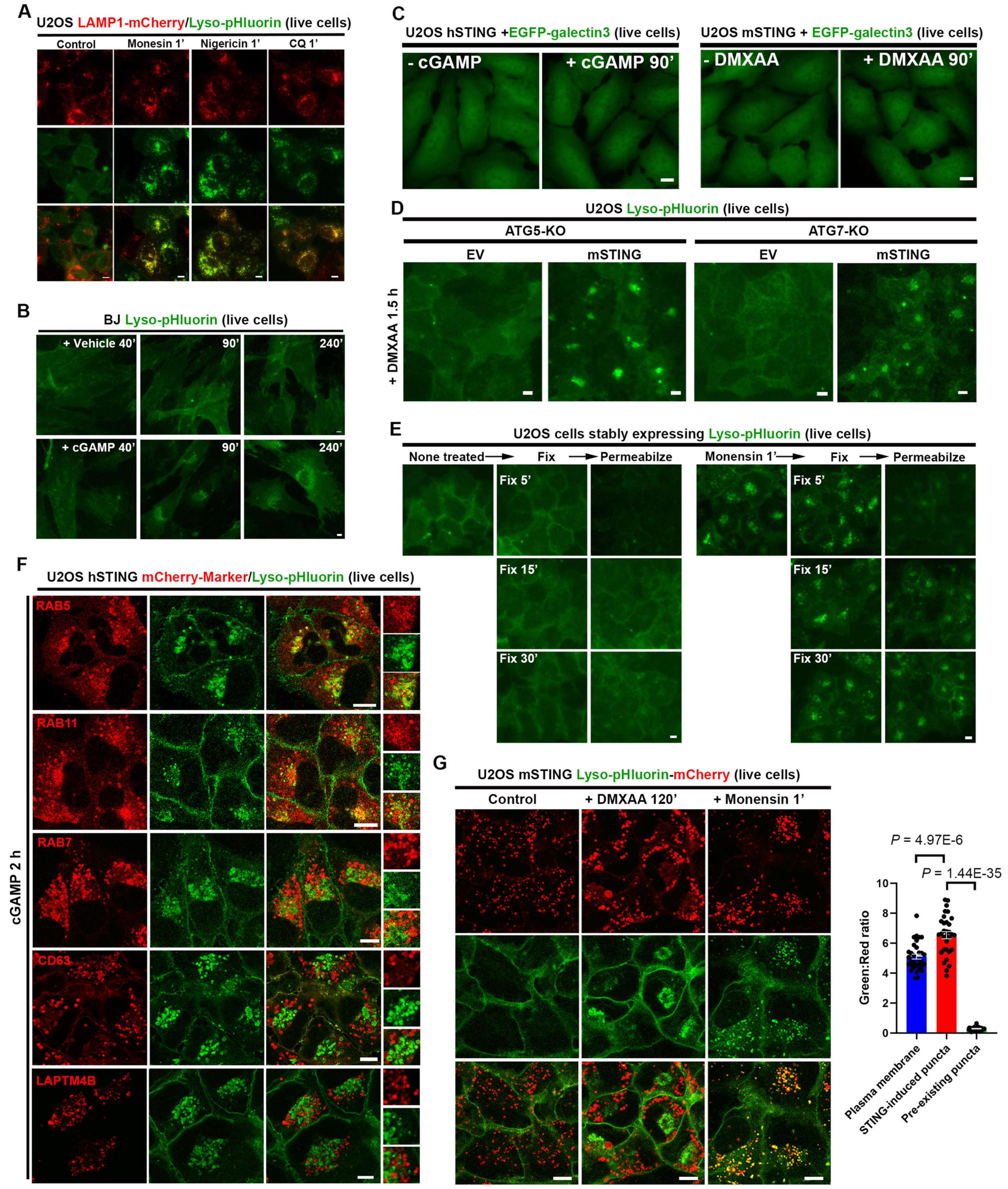
A
U2OS LAMP1-mCherry/Lyso-pHluorin (live cells)
Control
Monesin 1’
Nigericin 1’
CQ 1’
B
BJ Lyso-pHluorin (live cells)
+ Vehicle 40’
90’
240’
+ cGAMP 40’
90’
240’
C
U2OS hSTING +EGFP-galectin3 (live cells)
U2OS mSTING + EGFP-galectin3 (live cells)
- cGAMP
+ cGAMP 90’
- DMXAA
+ DMXAA 90’
D
U2OS Lyso-pHluorin (live cells)
ATG5-KO
ATG7-KO
EV
mSTING
EV
mSTING
+ DMXAA 1.5 h
E
U2OS cells stably expressing Lyso-pHluorin (live cells)
None treated
Fix
Permeabilze
Monensin 1’
Fix
Permeabilze
Fix 5’
Fix 15’
Fix 30’
Fix 5’
Fix 15’
Fix 30’
F
U2OS hSTING mCherry-Marker/Lyso-pHluorin (live cells)
cGAMP 2 h
RAB5
RAB11
RAB7
CD63
LAPTM4B
G
U2OS mSTING Lyso-pHluorin-mCherry (live cells)
Control
+ DMXAA 120’
+ Monensin 1’
P = 4.97E-6
P = 1.44E-35
Green:Red ratio
10
8
6
4
2
0
Plasma membrane
STING-induced puncta
Pre-existing puncta

◀ **Figure EV4. STING neutralizes the pH of post-Golgi vesicles, which is captured by lyso-pHluorin, an endolysosomal pH sensor.**

(**A**) Validation of Lyso-pHluorin as a pH probe by proton ionophores (monensin and nigericin) and weak base chloroquine (CQ). U2OS cells stably expressing lyso-pHluorin and LAMP1-mCherry were treated with monensin, nigericin, or CQ for 1 min, and the Lyso-pHluorin puncta were monitored by wide-field live-cell imaging. (**B**) cGAMP stimulates lyso-pHluorin puncta in wild-type BJ cells. BJ cells stably expressing lyso-pHluorin were treated with digitonin buffer alone (Vehicle) or with cGAMP for 10 min, changed back to original media, and chased for indicated time periods. Lyso-pHluorin puncta were monitored by wide-field live-cell imaging. (**C**) STING activation does not induce EGFP-galectin3 puncta. U2OS cells stably expressing EGFP-galectin3 and human or mouse STING (hSTING/mSTING) were stimulated with indicated STING agonists. The fluorescence of EGFP-galectin3 were monitored by wide-field live-cell imaging. (**D**) Normal Lyso-pHluorin puncta formation upon mSTING activation by DMXAA in ATG5-KO (left) and ATG7-KO (right) U2OS cells stably expressing mSTING and lyso-pHluorin. Images taken using wide-field live-cell imaging. (**E**) Setting up a protocol to fix lyso-pHluorin cells for the immunostaining of other proteins without triggering new lyso-pHluorin puncta by fixation or permeabilization. Cells were seeded three days before treatment. Monensin was used to induce pre-existing puncta before fixation. 30 min of fixation in 4% electron microscopy grade polyformaldehyde (PFA) followed by 2 min of permeabilization in 0.1% Triton X-100 in PBS were used as a standard protocol to examine the colocalization of lyso-pHluorin puncta with other proteins by immunostaining. Images in this panel were taken using wide-field microscopy. (**F**) Live-cell images showing STING-induced lyso-pHluorin puncta together with mCherry-tagged organelle markers. U2OS cells stably expressing hSTING, lyso-pHluorin, and mCherry-tagged organelle markers were stimulated with cGAMP and subjected to confocal live-cell imaging. LAPTM4B, lysosomal-associated protein transmembrane 4 beta. (**G**) Detecting STING-induced vesicle deacidification by Lyso-pHluorin-mCherry. Left, U2OS cells stably expressing lyso-pHluorin-mCherry were stimulated as indicated and subjected to confocal live-cell imaging. Right, quantification of the green: red signal ratios at different subcellular localizations. Mean ± SEM; $n = 32$ random measurements for each subcellular localization. Data Information: Bar, 10 μm for all cell imaging panels. Statistical significance was determined by unpaired, two-tailed t tests for all quantifications. Source data are available online for this figure.

 © The Author(s)

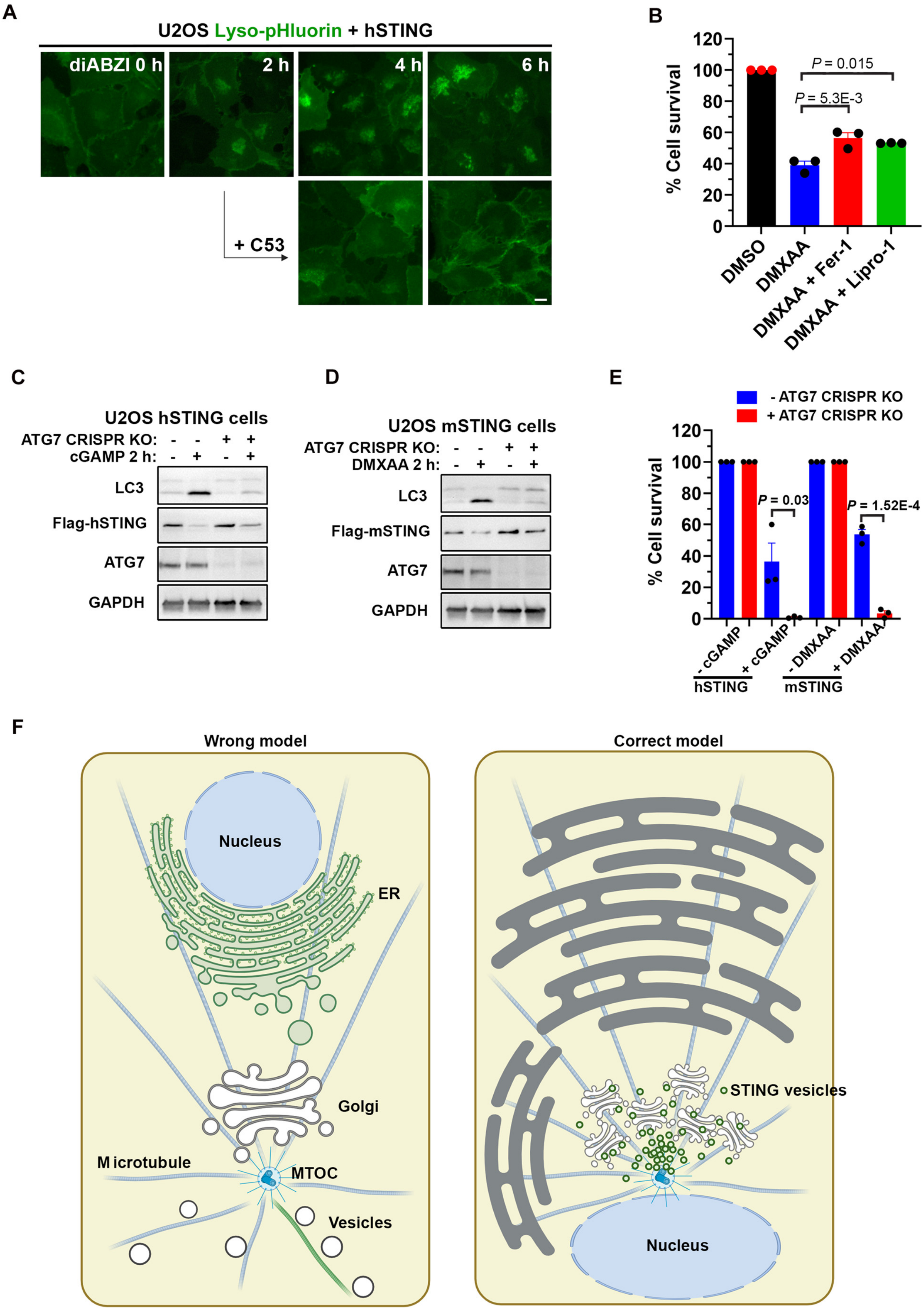
A
U2OS Lyso-pHluorin + hSTING
diABZI 0 h
2 h
4 h
6 h
+ C53
B
% Cell survival
120
100
80
60
40
20
0
P = 0.015
P = 5.3E-3
DMSO
DMXAA
DMXAA + Fer-1
DMXAA + Lipro-1
C
U2OS hSTING cells
ATG7 CRISPR KO: - - + +
cGAMP 2 h: - + - +
LC3
Flag-hSTING
ATG7
GAPDH
D
U2OS mSTING cells
ATG7 CRISPR KO: - - + +
DMXAA 2 h: - + - +
LC3
Flag-mSTING
ATG7
GAPDH
E
- ATG7 CRISPR KO
+ ATG7 CRISPR KO
% Cell survival
P = 0.03
P = 1.52E-4
- cGAMP
+ cGAMP
- DMXAA
+ DMXAA
hSTING
mSTING
F
Wrong model
Nucleus
ER
Golgi
Microtubule
MTOC
Vesicles
Correct model
STING vesicles
Nucleus

Figure EV5. Characterizing noncanonical functions of STING mediated through its transmembrane ion channel.

(**A**) C53 addition 2 h after diABZI treatment strongly suppresses and reverses STING-induced lyso-pHluorin puncta. U2OS cells stably expressing hSTING and lyso-pHluorin were stimulated as indicated and the lyso-pHluorin puncta were monitored by live-cell imaging. Quantified in Fig. 7C. (**B**) Ferroptosis inhibitors ferrostatin-1 (Fer-1), liprostatin-1 (Lipro-1) partially block STING-dependent cell death. U2OS cells stably expressing mSTING were treated as indicated and cell death was analyzed 24 h after treatment. Mean ± SEM; $n = 3$. (**C**, **D**) ATG7-KO blocks STING-induced LC3 lipidation. U2OS cells stably expressing human (**C**) or mouse (**D**) STING were treated with or without ATG7 CRISPR KO lentiviruses for more than 7 days, and the KO pools were further treated as indicated to activate STING. Cells were harvested 2 h after treatment for western blot analysis of LC3 lipidation. (**E**) ATG7-KO accelerates STING-dependent cell death. The same cells from (**C**, **D**) were treated as indicated and cell death was analyzed 24 h after treatment. Mean ± SEM; $n = 3$. (**F**) Schematic illustration of the relative localization of the key organelles involved in STING trafficking, from the ER to the Golgi to post-Golgi endosome-like vesicles, as well as microtubules that may facilitate STING trafficking. Left, a typical wrong online carton model of the same set of organelles, which limits ER to the perinuclear region. Right, the correct model showing ER extension throughout the cytoplasm with the Golgi complex assembled as a cluster of Golgi stacks around the microtubule-organization center (MTOC). Note that Golgi itself is also considered an MTOC. Activated STING traffics through the Golgi to form post-Golgi vesicle clusters, after which STING gradually traffics out of this cluster for lysosomal degradation. The post-Golgi STING vesicles are packed close to the Golgi complex and caution is needed when interpreting the localization of these perinuclear STING vesicles. Data Information: Bar, 10 μm for all cell imaging panels. Statistical significance was determined by unpaired, two-tailed t tests for all quantifications. Source data are available online for this figure.

 © The Author(s)
